# Diabetes mellitus, metformin’s target gene AMPK, and inflammatory bowel disease: A Mendelian randomization study

**DOI:** 10.1097/MD.0000000000041532

**Published:** 2025-02-14

**Authors:** Wei Wu, Huomu Tong, Yunsheng Li, Jia Cui

**Affiliations:** a Department of Geriatrics, Chun’an First People’s Hospital (Chun’an Branch of Zhejiang Provincial People’s Hospital), Hangzhou, Zhejiang Province, China; b Department of Endocrinology, Chun’an First People’s Hospital (Chun’an Branch of Zhejiang Provincial People’s Hospital), Hangzhou, Zhejiang Province, China.

**Keywords:** AMPK, Crohn disease, diabetes mellitus, inflammatory bowel disease, Mendelian randomization, metformin, ulcerative colitis

## Abstract

The causal relationship between inflammatory bowel disease (IBD) and diabetes mellitus remains unclear. The aim of this study was to delve into this association and investigate the correlation between AMP-activated protein kinase (AMPK), a target gene of metformin, and the risk of developing IBD. Researchers conducted a bidirectional two-sample Mendelian randomization analysis to examine causal relationships between IBD, including ulcerative colitis and Crohn disease (CD), and diabetes mellitus, encompassing both type 1 diabetes mellitus (T1DM) and type 2 diabetes mellitus (T2DM). Additionally, this study utilized AMPK-related variants associated with HbA1c (%) as instrumental variables for the metformin target gene AMPK to further investigate their association with the risk of IBD. The inverse variance weighted method was used as the primary analytical approach. Mendelian randomization analysis revealed a suggestive association between IBD and T1DM (*P* = .024). CD was associated with an increased risk of T1DM (*P* = .011). In the reverse analysis, T1DM also increased the risk of IBD (*P* = .043). No causal relationship was found between IBD and T2DM in either the forward or reverse analyses. In addition, this study did not find any significant effect of AMPK on IBD. In conclusion, this study suggests a bidirectional association between IBD and T1DM, in which CD may increase the risk of T1DM. However, no causal relationship was found between IBD and T2DM. Furthermore, our findings revealed that the metformin’s target gene AMPK had no significant effect on the onset of IBD.

## 1. Introduction

Inflammatory bowel disease (IBD) is a multisystemic illness that primarily involves Crohn disease (CD) and ulcerative colitis (UC) and typically manifests with diarrhea and abdominal pain as cardinal symptoms.^[1,2]^ In UC, the inflammatory response is strictly confined to the mucosal layers of the colon and the rectum. In contrast, the inflammatory characteristics of CD exhibit a more widespread distribution, potentially affecting any part of the digestive tract, from the mouth to the anus, and invading the full thickness of the intestinal wall. In addition, patients with IBD frequently exhibit a range of extraintestinal symptoms, including ocular inflammation, aphthous stomatitis, arthritis, skin lesions, liver disease, and complex neurological syndromes, all of which contribute to the overall complexity of the condition and lead to further therapeutic difficulties.^[3–6]^ Consequently, the escalating prevalence of IBD imposes a substantial economic and healthcare burden on society.^[7]^ Current research suggests that the etiology of IBD is multifactorial and encompasses genetic predisposition, environmental triggers, immune disorder and gut microbiota dysregulation.^[6,8,9]^

Diabetes mellitus (DM) is a chronic and systemic disease characterized by endocrine and metabolic disorders and is broadly classified into 4 distinct types: type 1 diabetes mellitus (T1DM), type 2 diabetes mellitus (T2DM), gestational diabetes, and other special types. The primary focus of this study was to investigate T1DM and T2DM patients. T1DM is caused by autoimmune inflammation that destroys the pancreatic β-cells, resulting in absolute insulin deficiency. T2DM is characterized by insulin resistance and the deterioration or loss of pancreatic β-cell function. Notably, the number of individuals with DM is increasing at an alarming rate each year. Given the sizable patient population with IBD and DM, the coexistence of these 2 conditions in clinical settings has become an inevitable phenomenon. However, the potential relationship between IBD and DM remains controversial in the academic community. Early genetic studies suggested a significant association between IBD and T1DM.^[10]^ However, another large-scale database study in the United States failed to find a correlation between IBD and T1DM.^[11]^ Furthermore, a study involving an average follow-up period of 5.1 years indicated a significant upward trend in the incidence of DM among IBD patients in South Korea, particularly among those with CD.^[12]^ Moreover, a large Danish cohort study encompassing 6,028,844 individuals revealed a marked increase in the risk of developing T2DM in patients with CD or UC.^[13]^

During the treatment of DM, the effect of antidiabetic agents on the occurrence and development of IBD is a key point that requires attention. Metformin, a widely used oral antidiabetic agent, has effects other than lowering the blood sugar levels. Studies have shown that metformin can also exhibit anti-inflammatory effects in a variety of diseases by activating the AMP-activated protein kinase (AMPK) pathway. Therefore, it is particularly important to investigate the potential causal relationship between IBD and DM, as well as the possible impact of the metformin target gene AMPK on IBD.

Mendelian randomization (MR) analysis relies on Mendelian inheritance laws and utilizes genetic variants as instrumental variables (IVs) to infer the causal effects of exposure factors on outcomes effectively.^[14]^ Since genetic variations are determined at birth and remain independent of disease progression, MR analysis can significantly reduce potential confounding factors, thus avoiding the interference of reverse causality bias and minimizing its impact on the analysis results.^[15]^ As an extension of traditional MR analysis, bidirectional MR further enhances the functionality of this approach by simultaneously exploring causality from both the exposure and outcome directions, providing a more powerful and precise means of determining the direction of causality.^[16]^ This study used a two-sample Mendelian randomization (MR) approach to assess the causal relationship between IBD and DM. Additionally, we investigated the potential effects of metformin’s target gene AMPK on IBD. The aim of this study was to provide new insights and evidence for related research.

## 2. Methods

### 2.1. Study design

**Figure 1.**
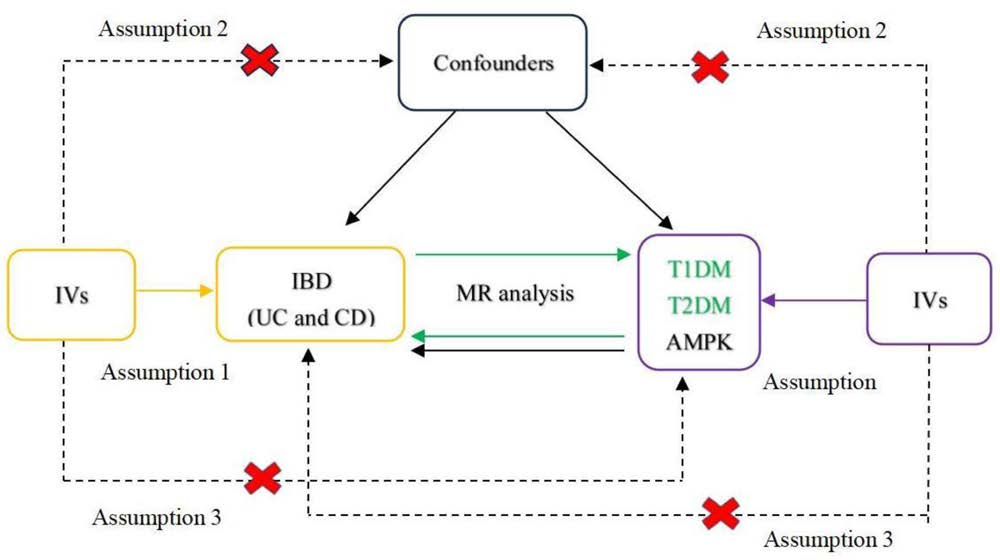
The flowchart of the MR analysis. AMPK = AMP-activated protein kinase, CD = Crohn disease, IBD = inflammatory bowel disease, IVs = instrumental variables, MR = Mendelian randomization, T1DM = type 1 diabetes mellitus, T2DM = type 2 diabetes mellitus, UC = ulcerative colitis.

This study adopted a bidirectional two-sample MR method to explore the potential causal relationship between IBD (including CD and UC) and DM (T1DM and T2DM). This study also employed the MR method to explore the potential role of the metformin target gene AMPK in IBD. The research process was logically and rigorously divided into 3 steps. First, we systematically investigated the genetic correlation between IBD as an exposure factor and DM as an outcome factor. Second, we conducted reverse analysis to further quantify the causal effects of DM on IBD, with the aim of comprehensively revealing the inherent link between the 2 diseases. Finally, using AMPK exposure and IBD as outcomes, we analyzed the potential impact of AMPK on IBD from a genetic perspective. A flowchart of MR analysis is shown in Figure 1. When conducting the MR analysis, we strictly adhered to the 3 core assumptions. First, we ensured that a strong correlation existed between the selected IVs and the exposure factor. Second, we guaranteed independence of the IVs from potential confounding factors. Finally, we confirmed that IVs influence the outcome solely through exposure factors, without involving other pathways. The data used in this study were obtained from genome-wide association studies (GWAS) and were officially authorized and approved by the corresponding ethics committee. Therefore, no additional approval from the ethics committee was required for this study.

### 2.2. Sources and subtypes of IBD

The data involved in this study, which were related to IBD (including UC and CD), were obtained exclusively from the IEU OpenGWAS project (https://gwas.mrcieu.ac.uk/). After careful screening, we deliberately selected 3 key GWAS datasets: ieu-a-31, which was specifically tailored for IBD; ieu-a-32, which was specifically tailored for UC; and ieu-a-30, which was specifically tailored for CD.^[17]^ These datasets were derived from in-depth research conducted by the International Inflammatory Bowel Disease Genetics Consortium on European populations. These datasets provided detailed and comprehensive summary information. Specifically, the dataset encompassed GWAS data from 12,882 individuals with IBD and 21,770 controls, 6968 patients with UC and 20,464 controls, and 5956 patients with CD and 14,927 controls.

### 2.3. Date sources of DM and its subtypes

In our study, the data for T1DM were obtained from IEU OpenGWAS; the GWAS ID was ebi-a-GCST90014023.^[18]^ The sample included 520,580 participants, including 18,942 T1DM patients and 501,638 controls, encompassing 59,999,551 single-nucleotide polymorphisms (SNPs). The T2DM data were derived from 122 GWAS conducted by the DIAbetes Meta-Analysis of Trans-Ethnic Association Studies consortium.^[19]^ These studies encompassed 5 distinct ancestry groups, with 51.1% of participants of European descent. The dataset included a total of 180,834 T2DM patients and 1159,055 controls. Data were publicly accessible through the official DIAMANTE website (http://diagram-consortium.org/). To ensure consistency in our research and to minimize potential bias arising from population diversity, we deliberately selected participants of European ancestry as our study subjects. For a detailed overview of IBD and DM data in this study, please refer to Table 1.

**Table 1:** Baseline characteristics of the inflammatory bowel disease and diabetes mellitus database.

Phenotype	Year	Dataset	Data source	Population	Sex	Ncase	Ncontrol	Number of SNPs	Reference
IBD	2015	ieu-a-31	IIBDGC	European	F/M	12,882	21,770	12,716,084	^[17]^
UC	2015	ieu-a-32	IIBDGC	European	F/M	6968	20,464	12,255,197	^[17]^
CD	2015	ieu-a-30	IIBDGC	European	F/M	5956	14,927	12,276,506	^[17]^
T1DM	2021	ebi-a-GCST90014023	N/A	European	F/M	18,942	501,638	59,999,551	^[18]^
T2DM	2022	N/A	DIAMANTE	European	F/M	180,834	1,159,055	10,454,875	^[19]^

CD = Crohn disease, DIAMANTE = DIAbetes Meta-ANalysis of Trans-Ethnic association studies, F/M = female/male, IBD = inflammatory bowel disease, IIBDGC = International Inflammatory Bowel Disease Genetics Consortium, T1DM = type 1 diabetes mellitus, T2DM = type 2 diabetes mellitus, UC = ulcerative colitis.

### 2.4. SNP selection

To ensure that the selected SNPs strictly adhered to the 3 fundamental assumptions of IVs in MR, we performed a series of meticulous quality control measures. Initially, we endeavored to identify SNPs that were closely associated with exposure factors and exhibited genome-wide significance (*P* < 5e‐8). Second, to minimize the potential bias arising from linkage disequilibrium and ensure the independence of the chosen SNPs, we established rigorous parameter thresholds in our clustering analysis: *r*^2^ = 0.001 and kb = 10,000. Subsequently, we calculated the F-statistic for each SNP using the formula *F* = [beta/SE]^2^ to focus on the strength of the exposure. We then set a threshold of *F* > 10 to ensure that the selected SNPs exerted a sufficient influence.

During the data processing phase, we leveraged the extensive resources of the PhenoScanner phenotype database (http://www.phenoscanner.medschl.cam.ac.uk/) to carefully screen and manually remove SNPs that may be associated with confounding factors or outcomes. To further enhance data quality, we implemented additional screening steps, including manual removal of palindromic SNPs and utilization of the MR Pleiotropy RESidual Sum and Outlier (MR-PRESSO) method, to identify and exclude outlier SNPs. Additionally, we employed the leave-one-out method to individually assess the potential impact of each SNP on the results, and cautiously excluded those that might introduce significant bias. During the screening process, we strictly adhered to the established standards, thereby ensuring the accuracy and reliability of the data. The specific details of the removed SNPs and selected SNPs are provided in Tables S1–S15, Supplemental Digital Content, http://links.lww.com/MD/O378.

### 2.5. The SNPs source of AMPK

We adopted the genetic tool constructed by Luo et al based on the target gene, AMPK.^[20]^ They first identified genetic variations within 1 Mb upstream and downstream of the AMPK subunit genes. Using the Meta-Analyses of Glucose and Insulin-Related Traits Consortium European population database and the UK Biobank, they screened for 44 SNPs that were significantly associated with glycated hemoglobin (*P* ≤ .05) and in a state of low linkage disequilibrium (*r*^2^ < 0.3). These SNPs were initially identified as the genetic tools for AMPK activation (Table S16, Supplemental Digital Content, http://links.lww.com/MD/O378). To ensure a strong correlation between the SNPs and exposure factors, SNPs with *P* > 5e‐8 were excluded from the study. Multiple websites, including the IEU Open Gwas and GWAS catalog (https://www.ebi.ac.uk/gwas/), were searched to identify SNPs related to confounding factors. Finally, 20 SNPs were selected as the genetic tools for AMPK.

### 2.6. Mendelian randomization analysis

We adopted inverse variance weighting (IVW) to avoid the interference of confounding variables and eliminate the impact of horizontal pleiotropy, thereby ensuring objectivity and unbiasedness of the research results.^[21]^ In addition to the IVW method, we used 4 other analysis methods: MR-Egger, weighted median, simple mode, and weighted mode. These methods allow for the consideration of the existence of horizontal pleiotropy in the analysis process, although they are slightly less effective than IVW in terms of statistical power. We used Bonferroni correction to address multiple testing issues with the aim of reducing Type I errors and bolster result reliability. Given our study parameters (0.05/6), the Bonferroni-adjusted significance threshold was set at 0.0083. *P*-values below this threshold indicate strong evidence of association, whereas values between 0.0083 and 0.05 suggest a potential association.

We applied Cochran Q test to evaluate data heterogeneity. When the test results indicated significant data heterogeneity (*P* < .05), we chose to use the random-effect IVW for analysis; otherwise, we used the fixed-effect IVW model. The MR-PRESSO test can effectively identify and correct outliers caused by horizontal pleiotropy, thereby ensuring the accuracy of MR analysis. The leave-one-out method was used to further investigate the potential impact of a single SNP. Specifically, we aimed to determine whether its presence might introduce bias that significantly affects the robustness of our research conclusions. All statistical analyses were performed using the R software (version 4.3.2) with “TwoSampleMR” (v.0.5.8) and “MR-PRESSO” (v.1.0).

## 3. Results

### 3.1. Causality between IBD and DM

**Figure 2.**
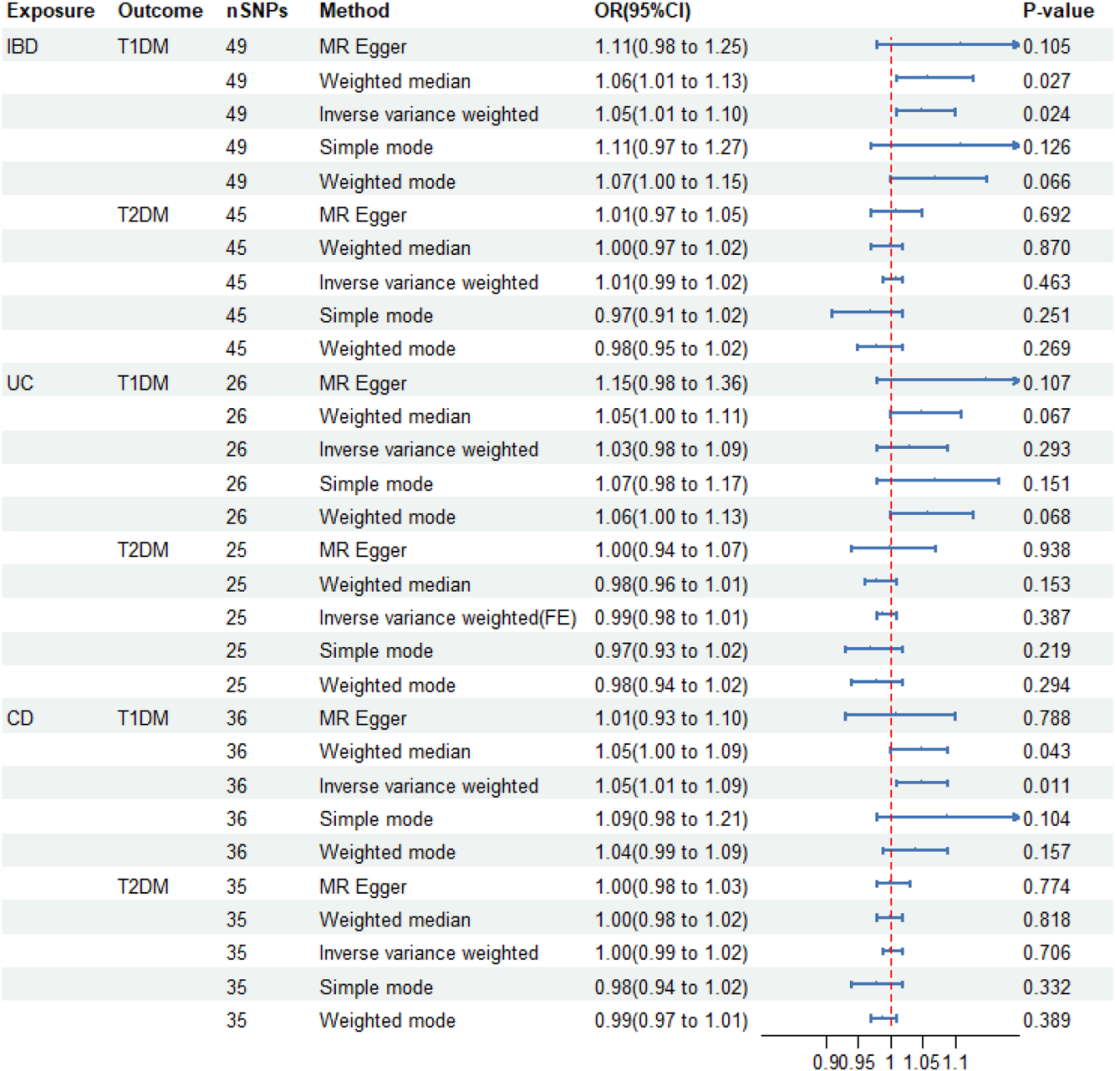
MR estimates the causal effect of IBD on DM. CI = confidence interval, CD = Crohn disease, DM = diabetes mellitus, FE = fixed effect, IBD = inflammatory bowel disease, MR = Mendelian randomization, OR = odds ratio, SNPs = single nucleotide-polymorphisms, T1DM = type 1 diabetes mellitus, T2DM = type 2 diabetes mellitus, UC = ulcerative colitis.

**Figure 3.**
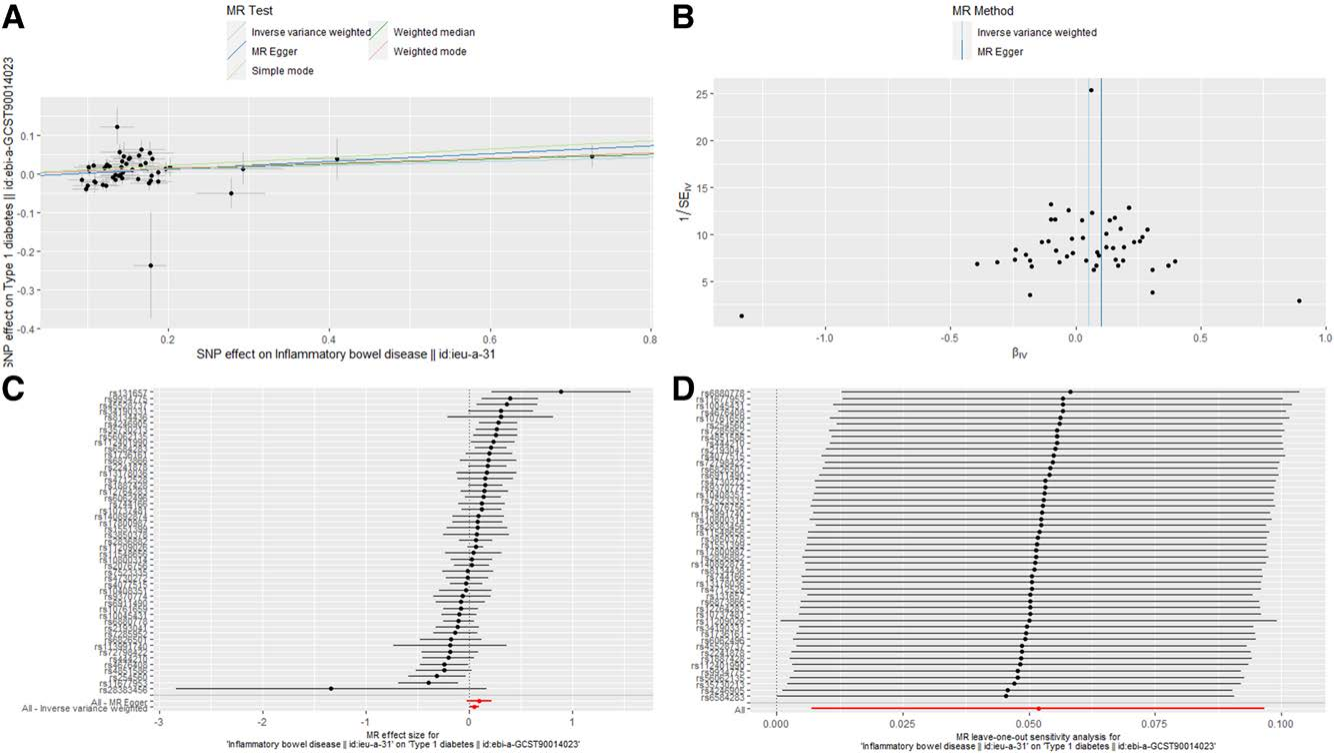
Scatter plot (A), funnel plot (B), forest plot (C), and leave-one-out analysis (D) of the causal effect of inflammatory bowel disease on type 1 diabetes mellitus.

In this study, 49 SNPs were screened and used as IVs to uncover the association between IBD and T1DM. The F-statistics of these IVs were significantly >10, ruling out the possibility of weak instrument bias. Given the observed data heterogeneity, we employed the IVW method with a random effects model for analysis. This IVW analysis revealed a suggestive causal effect of IBD on T1DM (OR = 1.05, 95% CI = 1.01–1.10, *P* = .024 [0.0083–0.05]) (Figs. 2 and 3). Furthermore, the results obtained from the weighted median approach were highly consistent with those obtained using the IVW method, providing additional support for the hypothesis that IBD may be a risk factor for the development of T1DM (Figs. 2 and 3).

Within the framework of the MR-Egger method, we found that the intercept for the effect of IBD on T1DM was ‐0.008 (*P* = .39), indicating that the selected IVs were not influenced by horizontal pleiotropy. Leave-one-out analysis confirmed that no individual SNP exhibited a potential outlier influence (Fig. 3). Moreover, the funnel plot did not display any evident outliers, which enhances the robustness of our findings (Fig. 3). When conducting a more detailed categorical analysis of IBD, we observed a similar suggestive causal effect of CD on T1DM (OR = 1.05, 95% CI = 1.01–1.09, *P* = .011 [0.0083–0.05]) (Fig. 2 and Fig. S1, Supplemental Digital Content, http://links.lww.com/MD/O378). However, no significant effect was observed for UC (OR = 1.03, 95% CI = 0.98–1.09, *P* = .293) (Fig. 2 and Fig. S2, Supplemental Digital Content, http://links.lww.com/MD/O378). These findings provide valuable insights for further exploration of the complex relationships between different types of IBD and T1DM.

However, the association between IBD and T2DM was not significant (OR = 1.01, 95% CI = 0.99–1.02, *P* = .463) (Fig. 2 and Fig. S3, Supplemental Digital Content, http://links.lww.com/MD/O378). Similarly, we did not find a causal association between CD (OR = 1.00, 95% CI = 0.99–1.02, *P* = .706), UC (OR = 0.99, 95% CI = 0.98–1.01, *P* = .387), and T2D (Fig. 2 and Figs. S4, Supplemental Digital Content, http://links.lww.com/MD/O378 and S5, Supplemental Digital Content, http://links.lww.com/MD/O378).

### 3.2. Reverse MR analysis of DM and IBD

**Figure 4.**
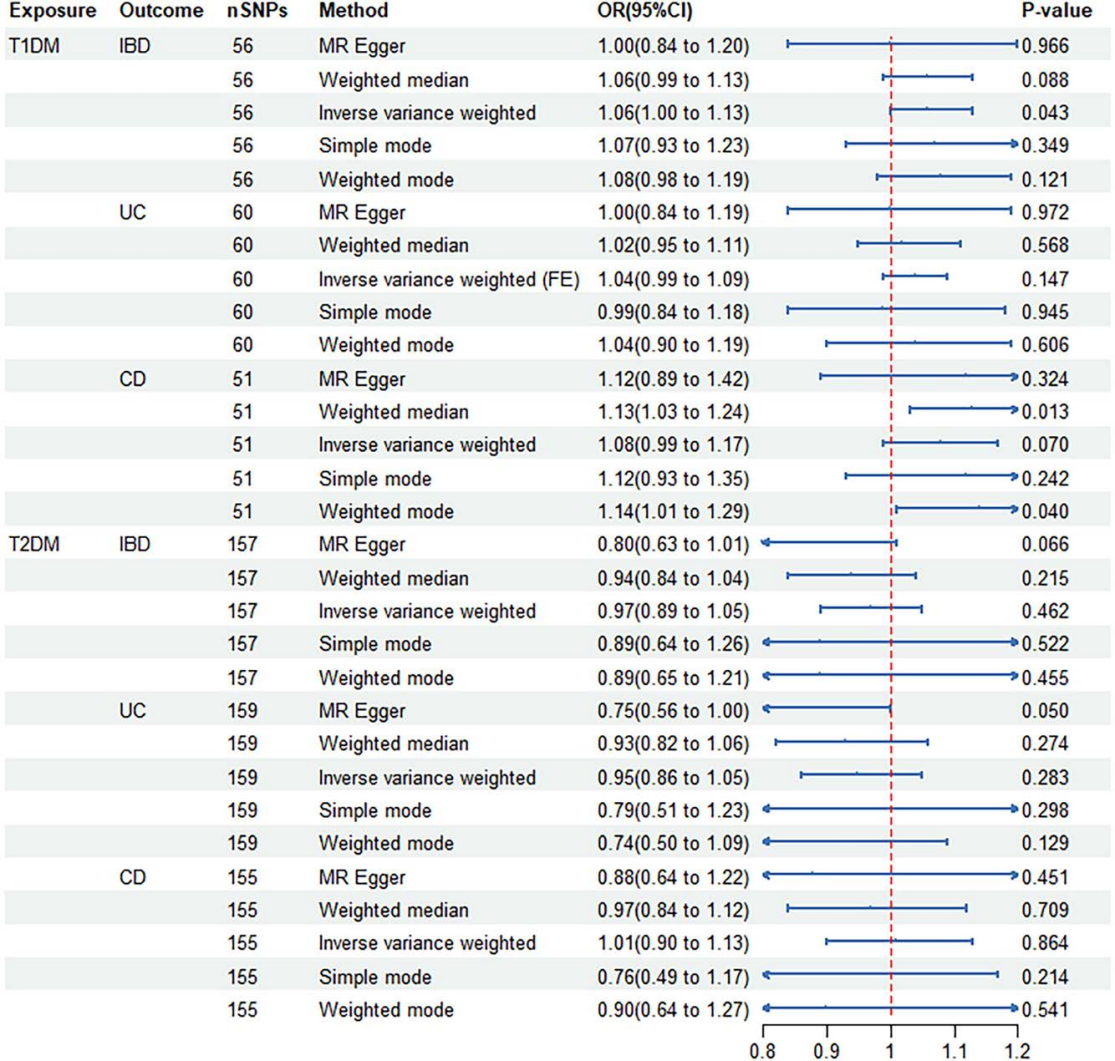
MR estimates the causal effect of DM on IBD. CD = Crohn disease, CI = confidence interval, DM = diabetes mellitus, FE = fixed effect, IBD = inflammatory bowel disease, MR = Mendelian randomization, OR = odds ratio, SNPs = single nucleotide polymorphisms, T1DM = type 1 diabetes mellitus, T2DM = type 2 diabetes mellitus, UC = ulcerative colitis.

**Figure 5.**
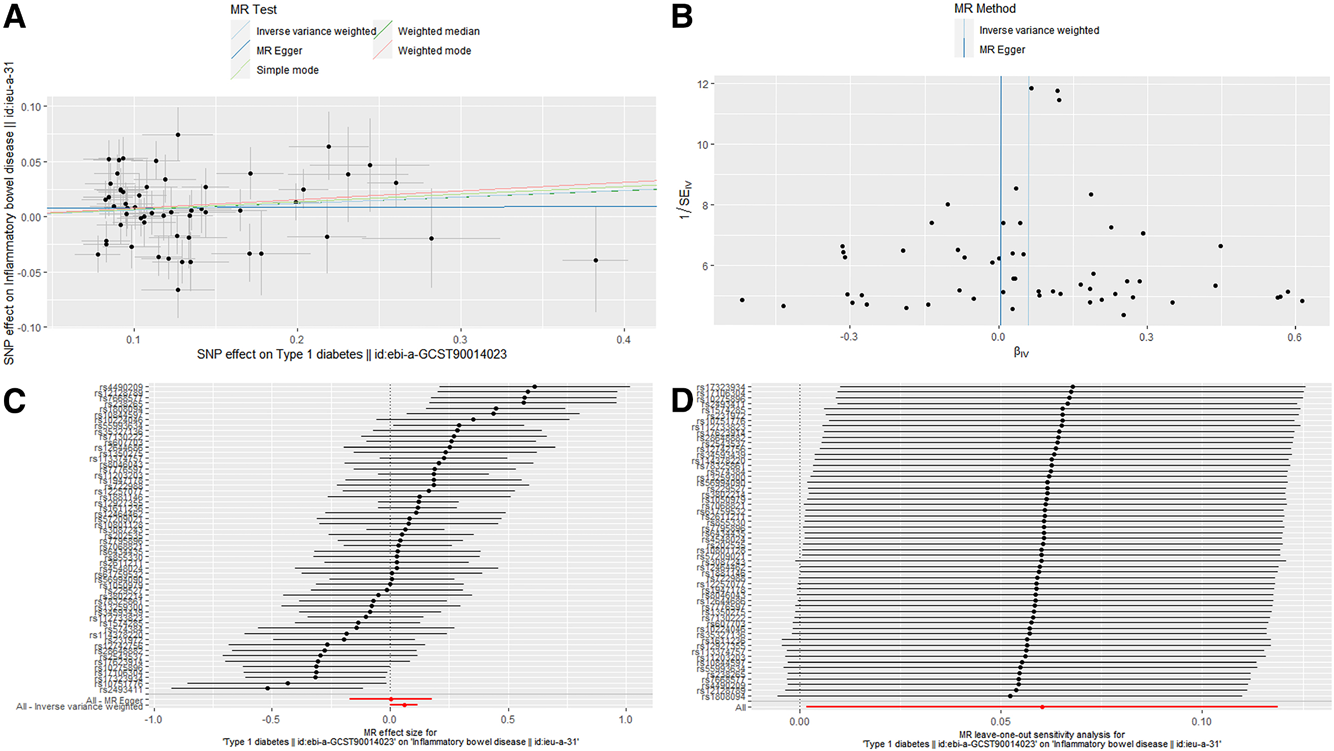
Scatter plot (A), funnel plot (B), forest plot (C), and leave-one-out analysis (D) of the causal effect of type 1 diabetes mellitus on inflammatory bowel disease.

In this study, we employed a reverse MR analysis approach with diabetes as the exposure factor and IBD as the outcome variable to investigate the potential causal effects of T1DM or T2DM on IBD. Through IVW analysis, we detected a suggestive causal association between T1DM and IBD only (OR = 1.06, 95% CI = 1.00–1.13, *P* = .043 [0.0083–0.05]) (Figs. 4 and 5). However, in further analysis, we did not observe significant causal associations between T1DM and CD (OR = 1.08, 95% CI = 0.99–1.17, *P* = .070) or UC (OR = 1.04, 95% CI = 0.99–1.09, *P* = .147) (Fig. 4 and Figs. S6, Supplemental Digital Content, http://links.lww.com/MD/O378 and S7, Supplemental Digital Content, http://links.lww.com/MD/O378). Similarly, T2DM did not significantly affect IBD (OR = 0.97, 95% CI = 0.89–1.05, *P* = .462), CD (OR = 1.01, 95% CI = 0.90–1.13, *P* = .864), or UC (OR = 0.95, 95% CI = 0.86–1.05, *P* = .283) (Fig. 4 and Figs. S8–S10, Supplemental Digital Content, http://links.lww.com/MD/O378). Furthermore, the results of sensitivity analysis indicated that the study was not substantially affected by pleiotropy (Table 2).

**Table 2:** Heterogeneity and pleiotropic test results.

Exposure	Outcome	Heterogeneity						Pleiotropy			MR-PRESSO (global test)	
		MR-Egger			IVW			MR-Egger				
		Q	Q_df	Q_pval	Q	Q_df	Q_pval	Intercept	SE	*P*	*RSSOBs*	*P*-value
IBD	T1DM	106.8793	47	1.47E‐06	108.5994	48	1.37E‐06	‐0.008475597	0.00974509	.3888682	112.4741	<.001
	T2DM	75.32585	43	0.001666865	75.34307	44	0.002266904	‐0.000349353	0.003524176	.9214955	78.68881	.004
UC	T1DM	55.61386667	24	0.000258592	60.181531	25	9.86982E‐05	‐0.021417127	0.015254572	.173133151	64.15367	<.001
	T2DM	35.39936331	23	0.047452743	35.54553385	24	0.060711305	‐0.00180331	0.005851595	.760723588	38.37417	.058
CD	T1DM	77.64154706	35	4.52037E‐05	80.54524726	36	2.95275E‐05	0.010339267	0.009037055	.26034799	84.48316	<.001
	T2DM	49.47028781	33	0.03268896	49.4943168	34	0.041837433	‐0.000410864	0.003245233	.900020922	52.26002	.046
T1DM	IBD	106.7446409	54	2.52428E‐05	107.6315619	55	2.87717E‐05	0.007591855	0.011333958	.505818804	110.9581	<.001
	UC	74.58763987	58	0.070189308	74.80253963	59	0.080432655	0.004605302	0.011265736	.684200484	77.26826	.073
	CD	89.73378192	49	0.000345118	90.01774723	50	0.000447304	‐0.005916284	0.015024371	.695452503	93.00617	<.001
T2DM	IBD	225.6160997	155	0.000184099	229.8321847	156	0.000110049	0.010789576	0.006339703	.090777915	232.7619	<.001
	UC	210.9101265	157	0.00265769	214.838771	158	0.001777707	0.013045979	0.007628764	.089222112	217.6442	.001
	CD	214.7769072	153	0.000733733	215.8333116	154	0.000748307	0.007417577	0.00855057	.387029336	218.5171	<.001
AMPK	IBD	29.21713395	18	0.045789098	29.3553525	19	0.060597256	‐0.004403795	0.015091293	.773768111	31.68212	.074
	UC	28.30793531	18	0.057521086	28.31602124	19	0.077532694	0.001339553	0.018681539	.9436277	30.33633	.101
	CD	22.22995227	18	0.221935838	22.26051409	19	0.271483371	‐0.002822475	0.01794211	.876751625	24.47422	.292

AMPK = AMP-activated protein kinase, CD = Crohn disease, IBD = inflammatory bowel disease, IVW = inverse variance weighting, MR-PRESSO = MR Pleiotropy RESidual Sum and Outlier, SE = standard error, T1DM = type 1 diabetes mellitus, T2DM = type 2 diabetes mellitus, UC = ulcerative colitis.

### 3.3. Causality between AMPK and IBD

**Figure 6.**
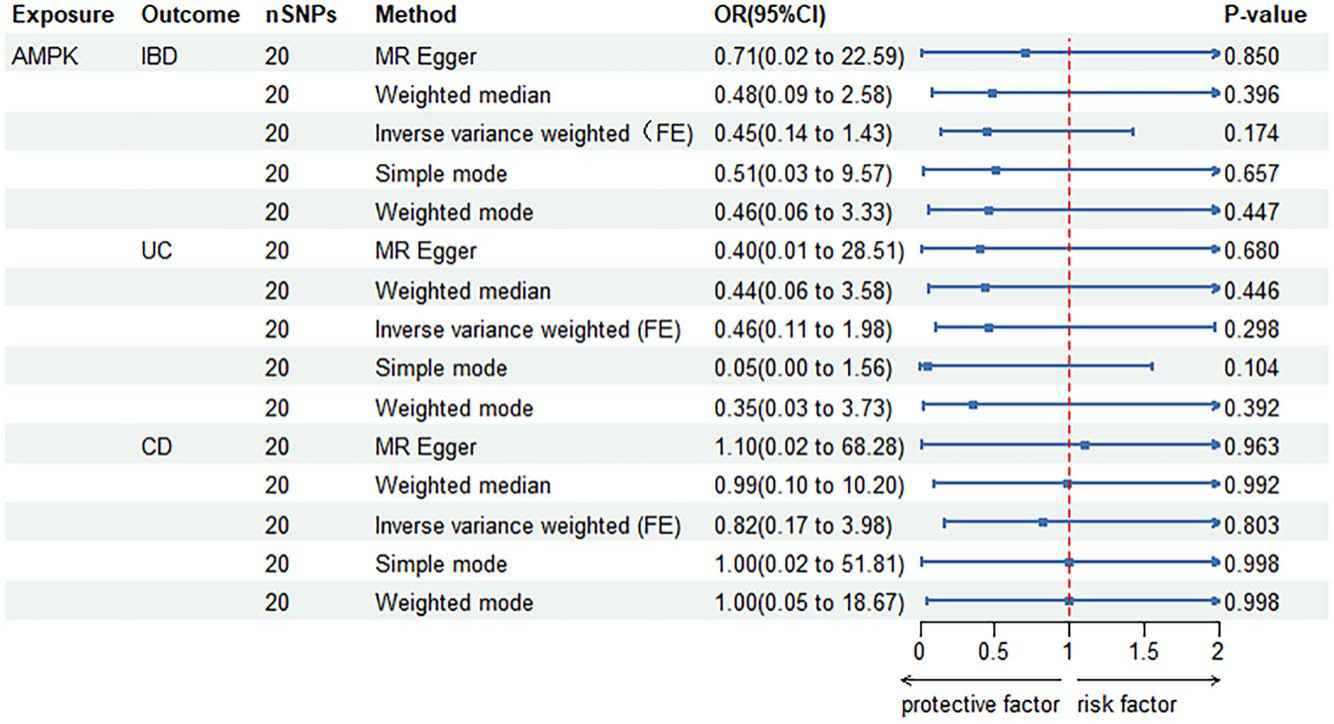
MR estimates the causal effect of AMPK on IBD. AMPK = AMP-activated protein kinase, CD = Crohn disease, CI = confidence interval, FE = fixed effect, IBD = inflammatory bowel disease, MR = Mendelian randomization, OR = odds ratio, SNPs = single-nucleotide polymorphisms, UC = ulcerative colitis.

Considering the heterogeneity between AMPK and IBD data, this study employed the IVW method based on a fixed-effects model for analysis. The IVW analysis revealed no significant causal association between AMPK and IBD (OR = 0.45, 95% CI = 0.14–1.43, *P* = .174) (Fig. 6). In addition, we explored the potential relationship between AMPK, CD, and UC. However, the analysis results did not show any significant causal association between AMPK and CD (OR = 0.82, 95% CI = 0.17–3.98, *P* = .803) or UC (OR = 0.46, 95% CI = 0.11–1.98, *P* = .298) (Fig. 6).

## 4. Discussion

Previous studies have often been limited to separately examining the unidirectional association between IBD and T1DM and between IBD and T2DM. However, this study utilized GWAS databases from European populations and adopted a two-sample bidirectional MR approach to comprehensively investigate the causal relationship between IBD (including CD and UC) and the risk of T1DM and T2DM. This method aimed to uncover the potentially complex interplay between these conditions. More importantly, the study also employed MR analysis to explore the impact of metformin’s target gene AMPK on IBD.

These findings suggest a potential bidirectional causal relationship between IBD and T1DM, particularly CD and T1DM, which may increase the risk of T1DM. However, no significant evidence has been found regarding the association between UC and T1DM. Neither the forward causal analysis of IBD to T2DM nor the reverse causal analysis of T2DM to IBD revealed a clear causal relationship. This conclusion also holds true when examining the relationships between UC, CD, and T2DM. Additionally, using AMPK as IVs for metformin, no significant effect of metformin on IBD risk was observed.

A Danish cross-sectional study found a significantly higher incidence of T1DM among patients with CD and UC.^[22]^ Jasser-Nitsche et al analyzed data from 65,147 children and adolescents with T1DM across 379 medical centers in Austria and Germany.^[23]^ Their findings are consistent with those of this study, showing a significantly increased risk of IBD among T1DM patients.^[23]^ However, a meta-analysis by Lu et al suggested no direct association between IBD and T1DM.^[24]^ Nonetheless, subgroup analysis indicated that individuals with UC or CD in certain regions were more likely to develop T1DM than those without IBD, suggesting that geographical factors may play a role in the comorbidity of IBD and T1DM.^[24]^

The potential link between IBD and T1DM stems from multiple immune mechanisms, dysbiosis of gut microbiota and environmental and genetic factors. However, these factors do not directly establish evidence of a necessary connection between the 2 conditions.^[25]^ In IBD, chronic inflammation caused by the immune system attacking the normal gut microbiota may be associated with dysfunctional regulatory T cells (Tregs).^[26]^ Similarly, Tregs dysfunction also exacerbates the development of T1DM.^[27,28]^ In T1DM, abnormal immune activation leads to the production of autoantibodies against pancreatic β-cells, such as glutamic acid decarboxylase antibodies, insulin autoantibodies, and islet cell antibodies.^[29]^ These antibodies mistakenly attack β-cells, resulting in absolute insulin deficiency. Notably, patients with IBD exhibit various autoantibodies and abnormal immune cell activation, displaying immunological characteristics similar to those of T1DM.

Inflammatory cytokines, such as IL-1β, IL-6, and TNF-α, along with chemokines, play key roles in promoting inflammatory responses and exacerbating local inflammation, which are crucial for the onset and progression of both IBD and T1DM. Specifically, IL-1β directly damages pancreatic β-cells, IL-6 affects glucose metabolism, and TNF-α plays a central role in peripheral insulin resistance. Notably, these cytokines are often targeted for IBD therapy.^[30]^ Din et al observed significantly increased frequencies of inflammatory markers associated with IBD exacerbation in patients with concurrent IBD and DM, indicating high levels of inflammatory activity in these patients.^[30]^ Santoso et al revealed a shared biological mechanism mediated by the major histocompatibility complex, between latent autoimmune diabetes in adults and CD and UC.^[31]^ Interestingly, certain major histocompatibility complex alleles, closely linked to T1DM risk, also show protective effects against UC and CD.^[32]^ Protein tyrosine phosphatase non-receptor type 22 appears to downregulate proinflammatory responses in CD, whereas it may have protective effects in T1DM.^[33,34]^

Gut microbiota dysbiosis may be a crucial link between the pathogenesis of IBD and T1DM. The gut, a multifunctional organ responsible for nutrient absorption, immune regulation, neural transmission, and hormone secretion, plays a pivotal role in maintaining glucose homeostasis. Trillions of microorganisms coexist in the gut and, maintain a symbiotic balance with the host to preserve health.^[35]^ However, factors such as gut infections, antibiotic overuse, and dietary changes can lead to dysbiosis, potentially activating autoimmune mechanisms and triggering diseases such as IBD and T1DM. Gut microbiota influences immunity by modulating Toll-like receptors and free fatty acid receptors, and their overactivation may drive the progression of DM and IBD.^[36]^ Additionally, gut dysbiosis can activate T-helper 17 lymphocytes, inducing systemic inflammation that may underlie the pathogenesis of both conditions.^[9]^ Reduced microbial diversity and increased harmful bacteria in the gut microbiota of IBD patients may be critical for the development of inflammation. Similarly, T1DM patients may experience gut microbial imbalances that affect the normal immune function.^[37]^ By correcting dysbiosis, probiotics have the potential to promote immune tolerance and prevent the initiation of autoimmune responses, thus offering a promising avenue for autoimmune disease prevention.^[38]^ In genetically predisposed individuals, specific viral and bacterial infections can activate immune responses, leading to IBD or T1DM.

The gut–brain axis is a bidirectional communication pathway comprising the nervous system, microbial metabolites, and immune system.^[39]^ It connects the gut microbiota with the brain, influences metabolic regulation, appetite control, and overall health, and has become a focus of research on obesity and insulin resistance in T2DM. The brain perceives and regulates glucose homeostasis by receiving feedback signals from the gastrointestinal tract.^[40]^ Enteroendocrine cells in the gut epithelium act as key sensors, establishing the gut as the largest endocrine organ in the body. These cells detect changes in the gut environment and release gut peptides, such as glucagon-like peptide-1 (GLP-1), glucose-dependent insulinotropic polypeptide (GIP), and cholecystokinin, which regulate metabolism via the gut–brain axis mechanisms.^[41]^

The human gut microbiota is primarily classified into 5 categories, with Firmicutes and Bacteroidetes being the dominant ones.^[39]^ Studies suggest that obese individuals exhibit an increased proportion of Firmicutes and a relative reduction in Bacteroidetes, whereas a vegetarian diet promotes the growth of Bacteroidetes.^[37]^ Short-chain fatty acids (SCFAs), such as acetate, propionate, and butyrate, are fermentation byproducts of carbohydrates produced by the gut bacteria. These SCFAs, predominantly generated in the distal gastrointestinal tract, exhibit multiple beneficial effects, including anti-inflammatory effects, glucose homeostasis enhancement, insulin sensitivity regulation, and brain integrity maintenance.^[24,42]^ In insulin-resistant individuals, these beneficial SCFAs are reduced, potentially exacerbating inflammation and metabolic dysfunctions. Dysbiosis may increase intestinal permeability, leading to “leaky gut,” systemic inflammation, and impaired insulin signaling. The gut microbiota also produces neurotransmitters, such as dopamine, gamma-aminobutyric acid, and serotonin, which influence appetite and food preferences. Disruptions in neurotransmitter levels can interfere with satiety signals, leading to overeating and weight gain. Additionally, they regulate the secretion of hunger and satiety hormones, in which imbalances may promote obesity and insulin resistance. Probiotics have demonstrated effective anti-obesity properties by enhancing satiety, improving insulin resistance, and optimizing gut microbiota composition.^[43]^ Hence, the gut–brain axis plays a pivotal role in obesity and insulin-resistant T2DM.

A large-scale nationwide cohort study in Denmark revealed that individuals with IBD had a 50% higher risk of T2DM than the general population.^[13]^ However, this study did not establish a direct association between IBD and T2DM. Research by Kang et al based on nationwide Korean population data, indicated a significantly increased prevalence of DM among patients with IBD, particularly those with CD, consistent with the findings of the present study.^[12]^ Notably, this risk is more pronounced in younger patients.^[12]^

Dysregulation of the immune system is a common pathological characteristic of both DM and IBD. The prolonged presence of DM may exacerbate the immune system imbalance and increase the risk of developing IBD. Chronic hyperglycemia and metabolic disturbances may intensify immune dysfunction. Hyperglycemia triggers inflammatory cascades and increases susceptibility to autoimmune diseases such as IBD. Furthermore, hyperglycemia compromises the integrity of the intestinal barrier, increasing its permeability and rendering the gut more susceptible to disruption.^[35]^ The cumulative duration of diabetes is directly correlated with prolonged exposure to adverse metabolic environments, which significantly increases the risk of IBD.

T1DM typically manifests during childhood or adolescence, coinciding with the peak incidence of autoimmune diseases.^[44]^ As individuals diagnosed with T1DM at an early age progress into adulthood, their extended disease duration may result in imbalanced inflammatory responses in the immune system, thus promoting the development of IBD. During critical periods of immune system development in youth, environmental triggers such as infections or dietary factors may precipitate both conditions. Significant alterations and disruptions in gut microbiota diversity during childhood and adolescence not only correlate with insulin resistance but may also trigger inflammatory conditions such as IBD. Among young individuals with preexisting DM, changes in the gut microbiota may further increase the risk of developing IBD.

Incretins are hormones released by the gut upon food intake, particularly carbohydrates, and include GLP-1 and GIP. GLP-1, secreted by ileal L-cells, promotes insulin release, but its action is notably diminished in patients.^[45]^ Changes in the gut environment of patients with IBD, including inflammation, altered secretion of gut hormones, and impaired absorption, collectively influence the effects of incretin. Gut inflammation disrupts GLP-1 and GIP secretion, adversely affecting insulin release and glucose metabolism. The inflammatory environment associated with IBD may reduce GLP-1 levels and contribute to glucose regulatory disorders.

GLP-1 receptor agonists (GLP-1RAs) mimic endogenous GLP-1 effects, enhance glucose-dependent insulin secretion, inhibit glucagon release, slow gastric emptying, and increase satiety, thereby aiding in weight management and reducing cardiovascular risk.^[39]^ GLP-1RAs also exhibit anti-inflammatory properties, potentially alleviating intestinal inflammation in IBD patients by modulating immune function and inhibiting proinflammatory cytokine release, although the exact anti-inflammatory mechanism remains unclear. Metabolic syndromes, particularly insulin resistance, are often associated with IBD. GLP-1RAs can mitigate metabolic complications arising from IBD by improving insulin sensitivity and glycemic control. However, changes in the effects of incretin owing to IBD add complexity to diabetes management. Although GLP-1RAs offer glycemic control improvements for T2DM patients and may benefit IBD management, further research is needed to elucidate their precise impact on IBD and their suitability as therapeutic options.

The interaction between IBD and DM is influenced by primary therapeutic agents. Recent studies have shown that commonly used IBD medications such as adalimumab and infliximab significantly improve insulin sensitivity, reduce insulin resistance, and effectively lower blood glucose levels.^[46]^ Additionally, sulfasalazine and 5-aminosalicylic acid have been shown to have glucose-lowering effects.^[46]^ However, corticosteroids may induce hyperglycemia through various mechanisms, including upregulation of gluconeogenesis, enhancement of insulin resistance, and inhibition of glucose uptake by adipose tissue.^[12]^ These effects can lead to steroid-induced DM.^[47]^

Among various antidiabetic drugs, metformin is widely used as a first-line therapy owing to its significant glucose-lowering effects, excellent safety profile, superior cost-effectiveness, and broad applicability across populations. Studies have suggested that metformin activates the AMPK pathway to inhibit c-Jun N-terminal kinase, thereby enhancing intestinal barrier integrity and reducing the risk of gut infections.^[48]^ Moreover, metformin modulates autophagy in IBD, reduces proinflammatory cytokine release and downregulates inflammatory process.^[49]^ However, we did not identify a significant effect of AMPK, target gene of metformin, on IBD risk. Additionally, a meta-analysis by Li et al revealed that dipeptidyl peptidase-4 inhibitors (DPP4i) might increase the risk of UC but decrease the risk of CD in T2DM patients. Notably, no significant changes in overall IBD risk were observed with DPP4i use.^[50]^ Another study by Abrahami et al indicated that DPP4i might increase IBD risk in T2DM patients.^[51]^ Furthermore, studies exploring other therapeutic agents suggest that GLP-1 analogues positively reduce intestinal inflammation, whereas pioglitazone has an insignificant effect on IBD.^[52,53]^

Clinical research on disease associations faces numerous challenges including patient variability, disease complexity, drug diversity, design limitations, measurement errors, and laboratory inconsistencies. To address these challenges, we used MR to explore the causal relationship between IBD and DM from a genetic perspective. Additionally, we investigated the potential impact of metformin on IBD. To ensure the robustness of the study, we used multiple methods to assess pleiotropy and heterogeneity, striving to minimize confounding variables and potential reverse causality.

## 5. Conclusion

In conclusion, the results of this bidirectional MR study suggest the existence of a bidirectional causal relationship between IBD and T1DM. However, no such association was found between IBD and T2DM. Assessment of the effect of metformin on IBD risk using target-gene proxies showed no impact. This study contributes to the understanding of the complex interplay between IBD and DM while providing valuable insights for future research and clinical applications.

## Acknowledgments

We are grateful to all GWAS researchers and volunteers for their contribution to the statistical data used in this study, and we appreciate the investigators for sharing the data.

## Author contributions

**Conceptualization:** Wei Wu, Jia Cui.

**Data curation:** Wei Wu.

**Investigation:** Wei Wu.

**Methodology:** Wei Wu, Jia Cui.

**Project administration:** Jia Cui.

**Software:** Wei Wu.

**Writing – original draft:** Huomu Tong, Yunsheng Li.

**Writing – review & editing:** Jia Cui.

## Supplementary Material


