# Supplementary material for: Diabetes mellitus, metformin’s target gene AMPK, and inflammatory bowel disease: A Mendelian randomization study
Source: Medicine (Baltimore). 2025 Feb 14;104(7):e41532. doi: 10.1097/MD.0000000000041532 (PMC11835072; doi:10.1097/MD.0000000000041532)
Supplement: Supplementary file 1 [file medi-104-e41532-s001.pdf]

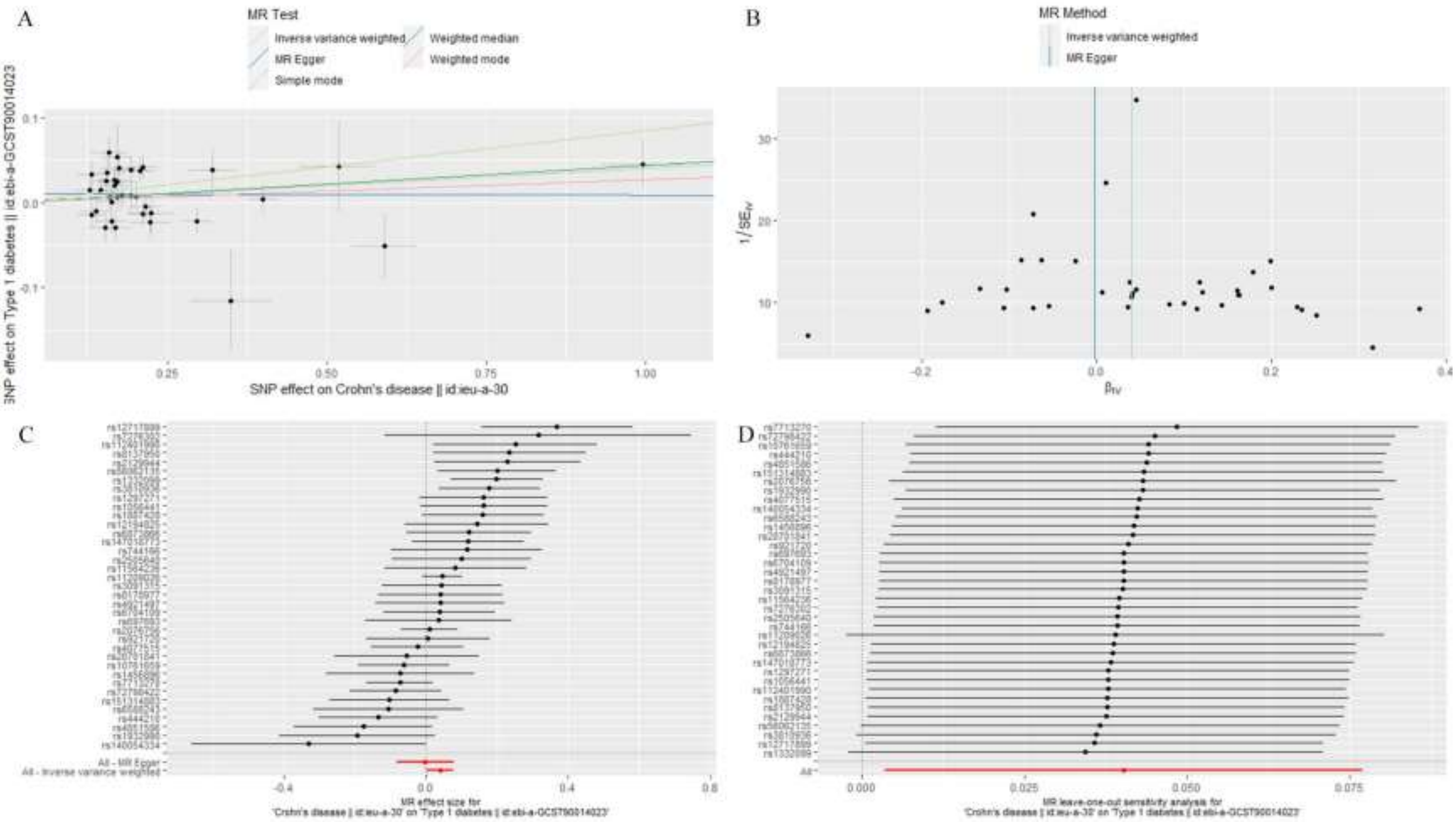

Fig. S1 Scatter plot (A), funnel plot (B), forest plot (C) and leave-one-out analysis (D) of the causal effect of Crohn's disease on type 1 diabetes mellitus.

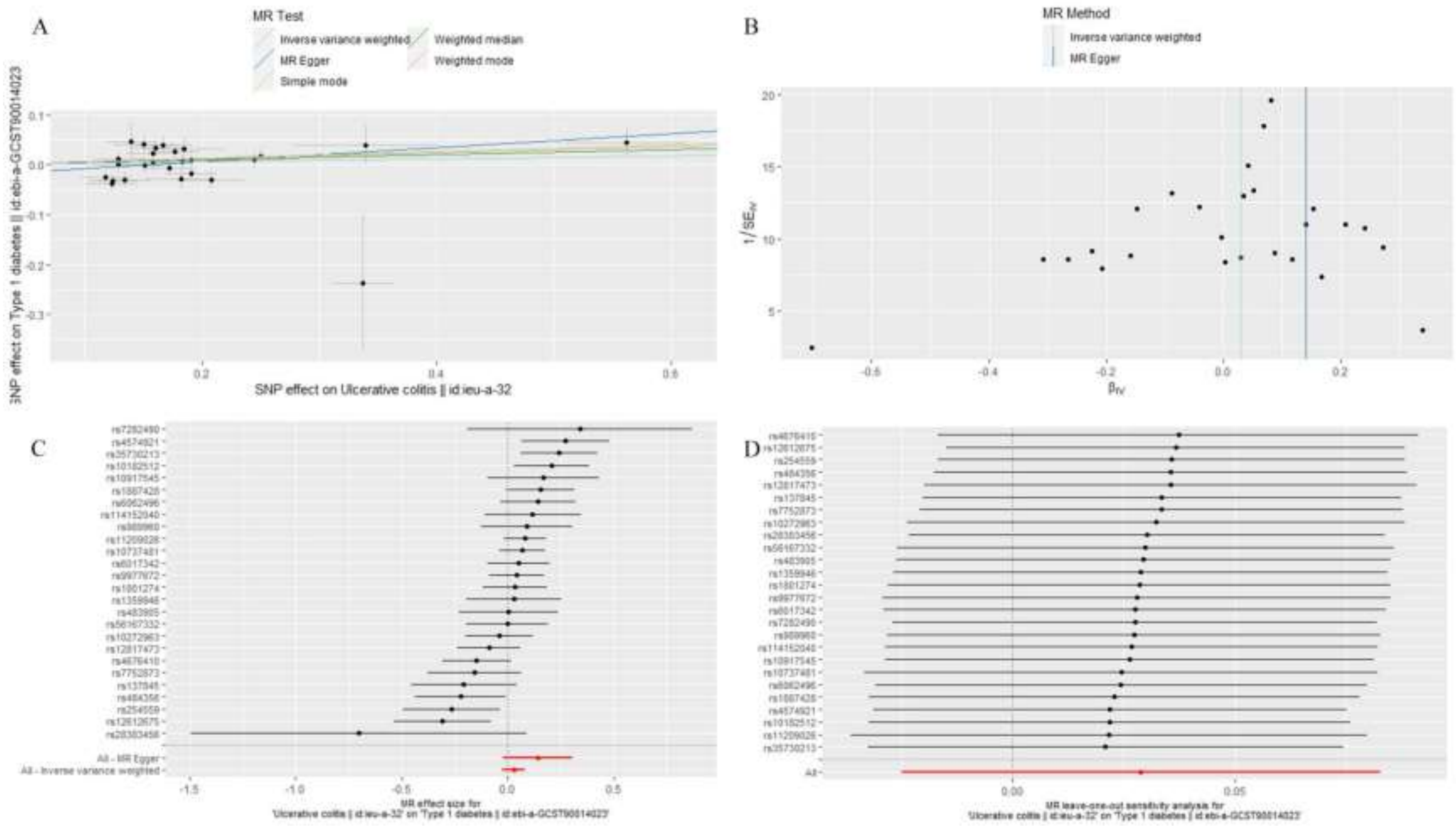

Fig. S2 Scatter plot (A), funnel plot (B), forest plot (C) and leave-one-out analysis (D) of the causal effect of ulcerative colitis on type 1 diabetes mellitus.

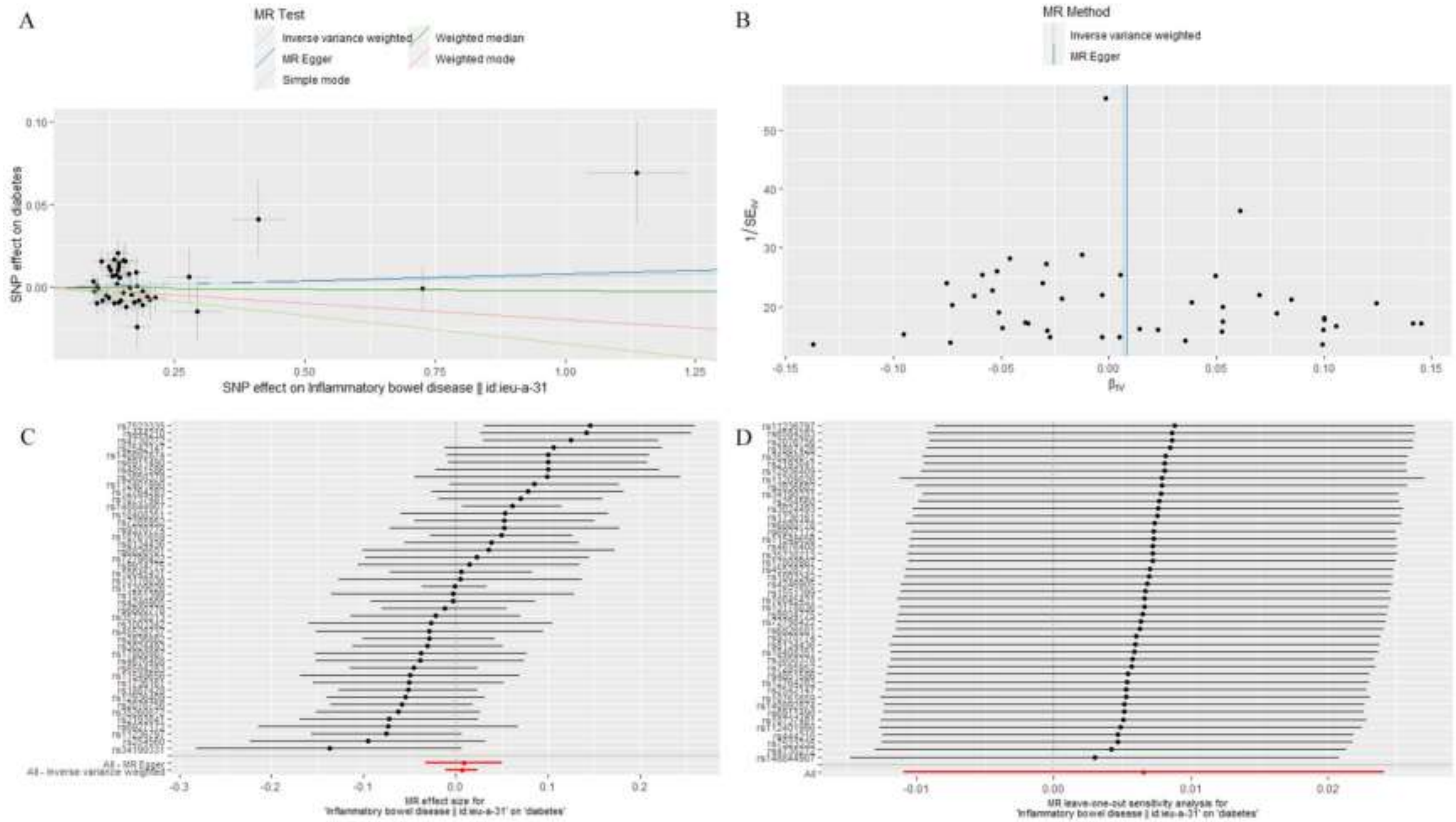

Fig. S3 Scatter plot (A), funnel plot (B), forest plot (C) and leave-one-out analysis (D) of the causal effect of inflammatory bowel disease on type 2 diabetes mellitus.

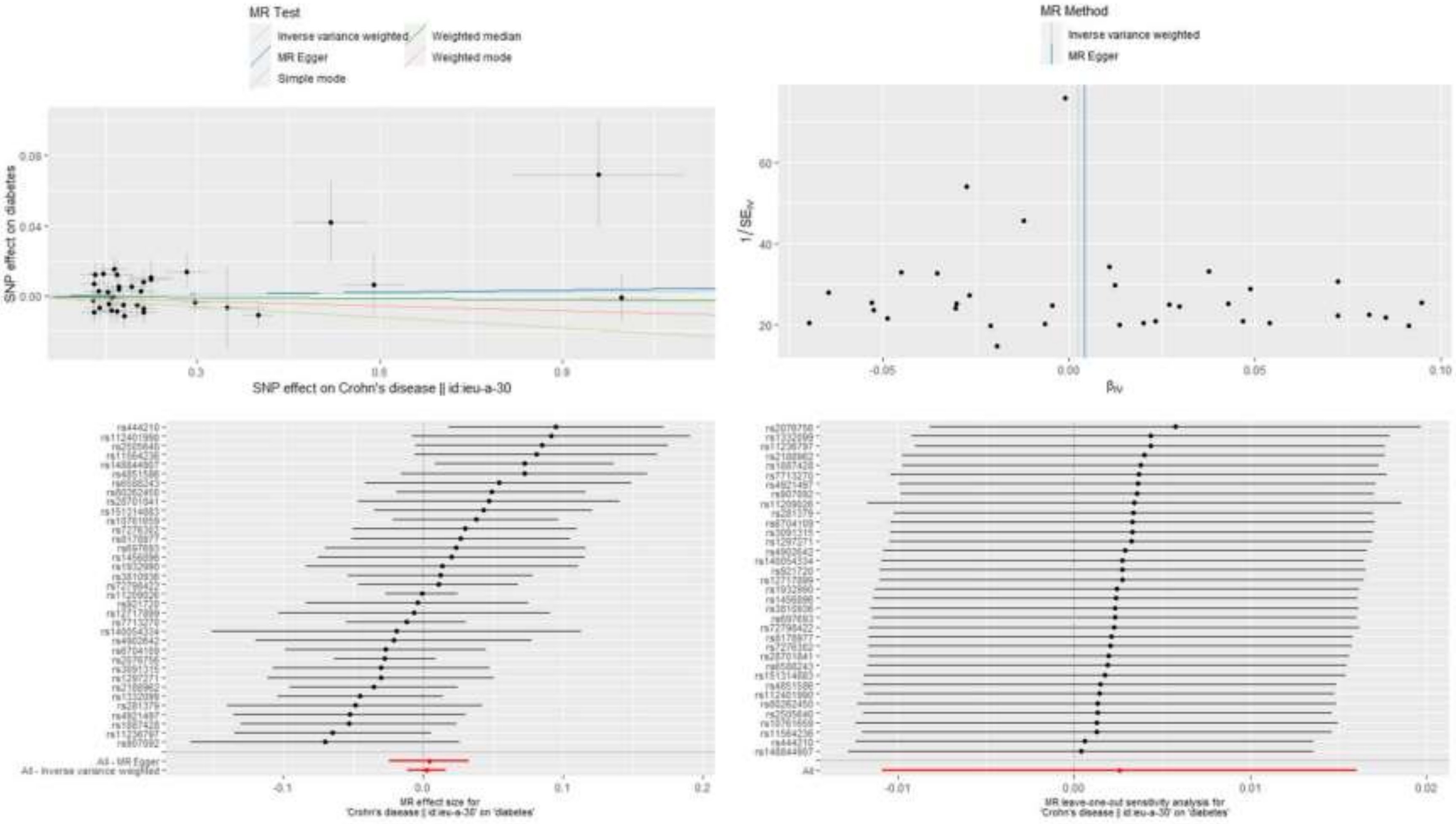

Fig. S4 Scatter plot (A), funnel plot (B), forest plot (C) and leave-one-out analysis (D) of the causal effect of Crohn's disease on type 2 diabetes mellitus.

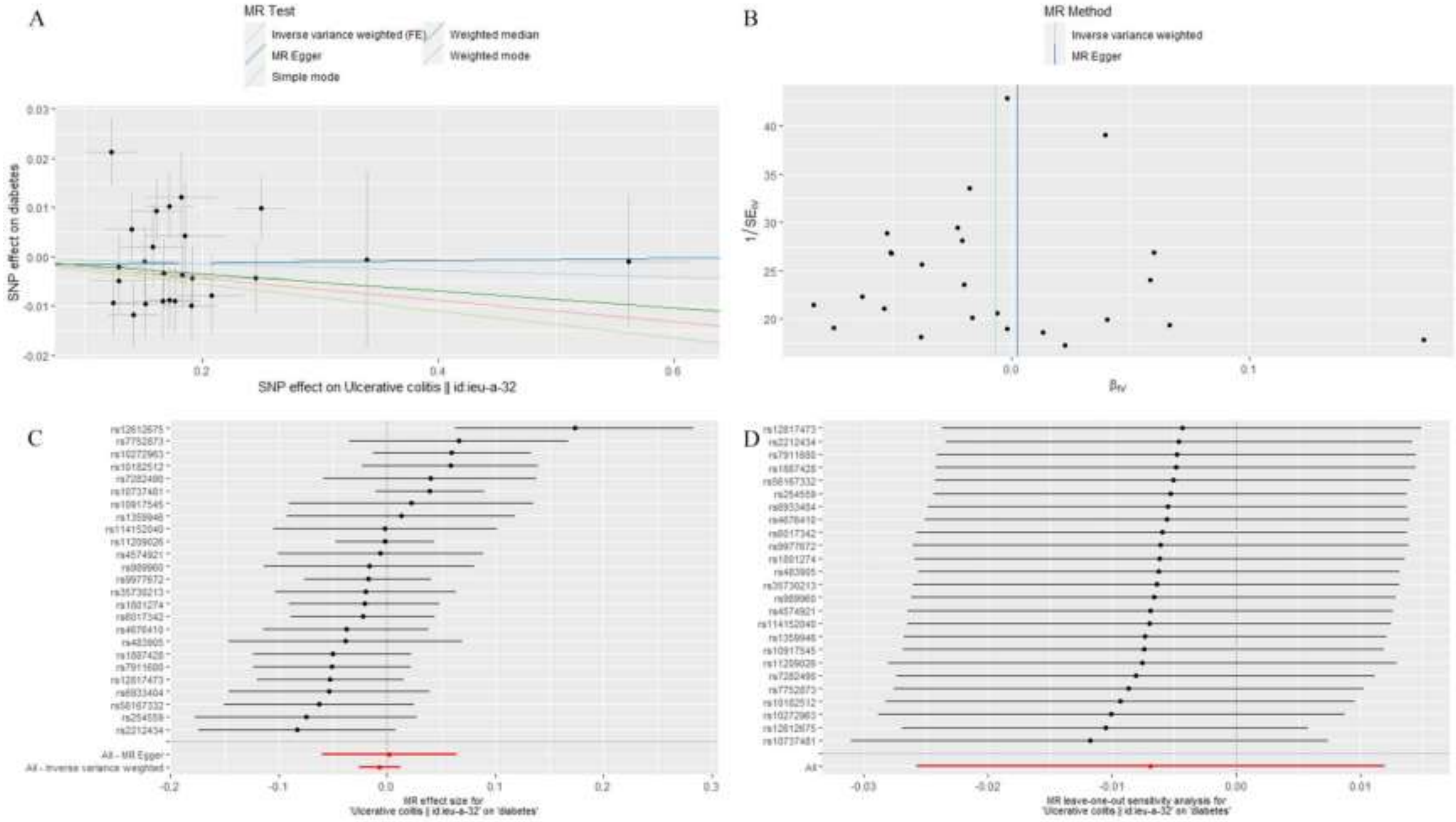

Fig. S5 Scatter plot (A), funnel plot (B), forest plot (C) and leave-one-out analysis (D) of the causal effect of ulcerative colitis on type 2 diabetes mellitus.

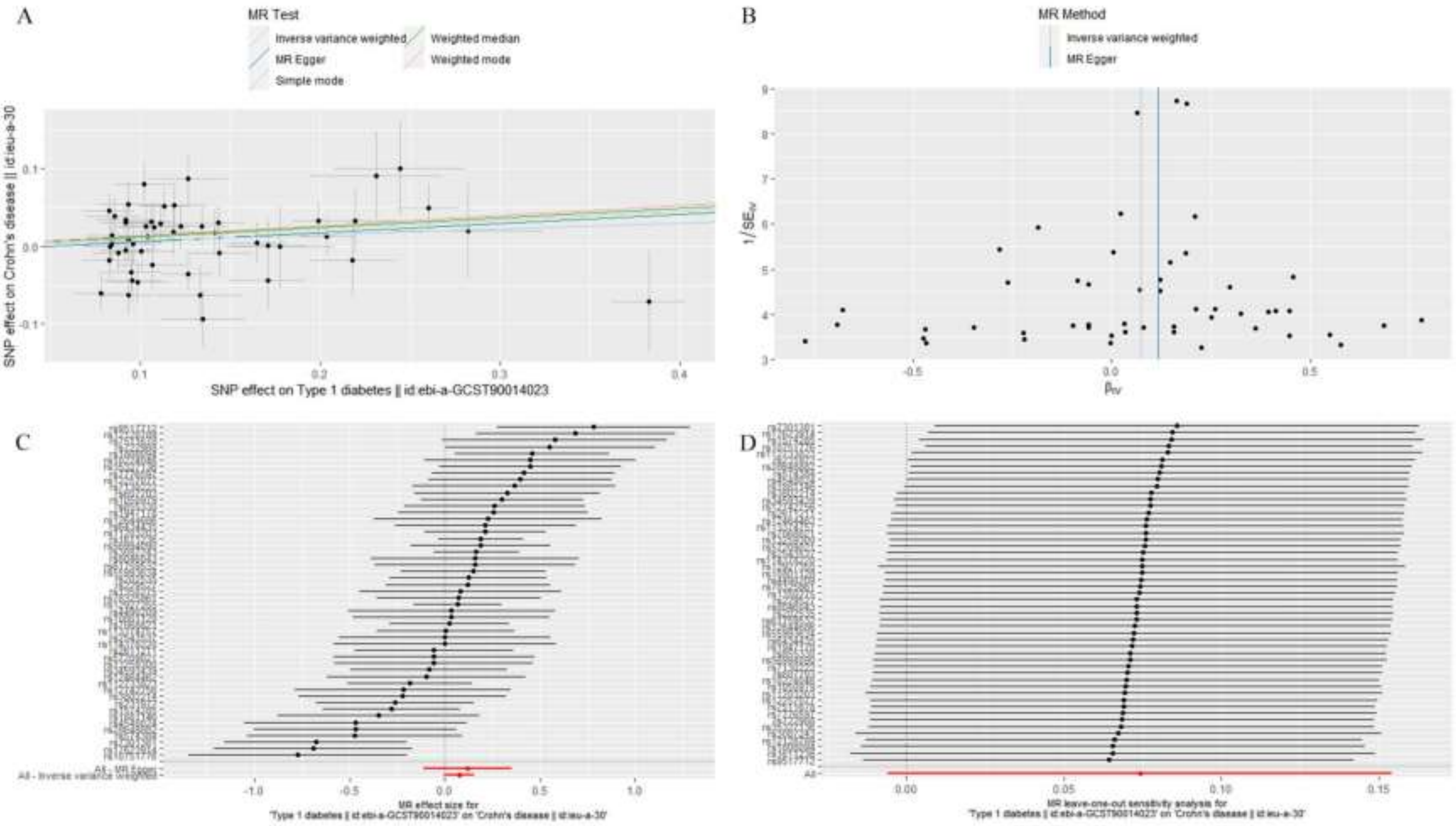

Fig. S6 Scatter plot (A), funnel plot (B), forest plot (C) and leave-one-out analysis (D) of the causal effect of type 1 diabetes mellitus on Crohn's disease.

T

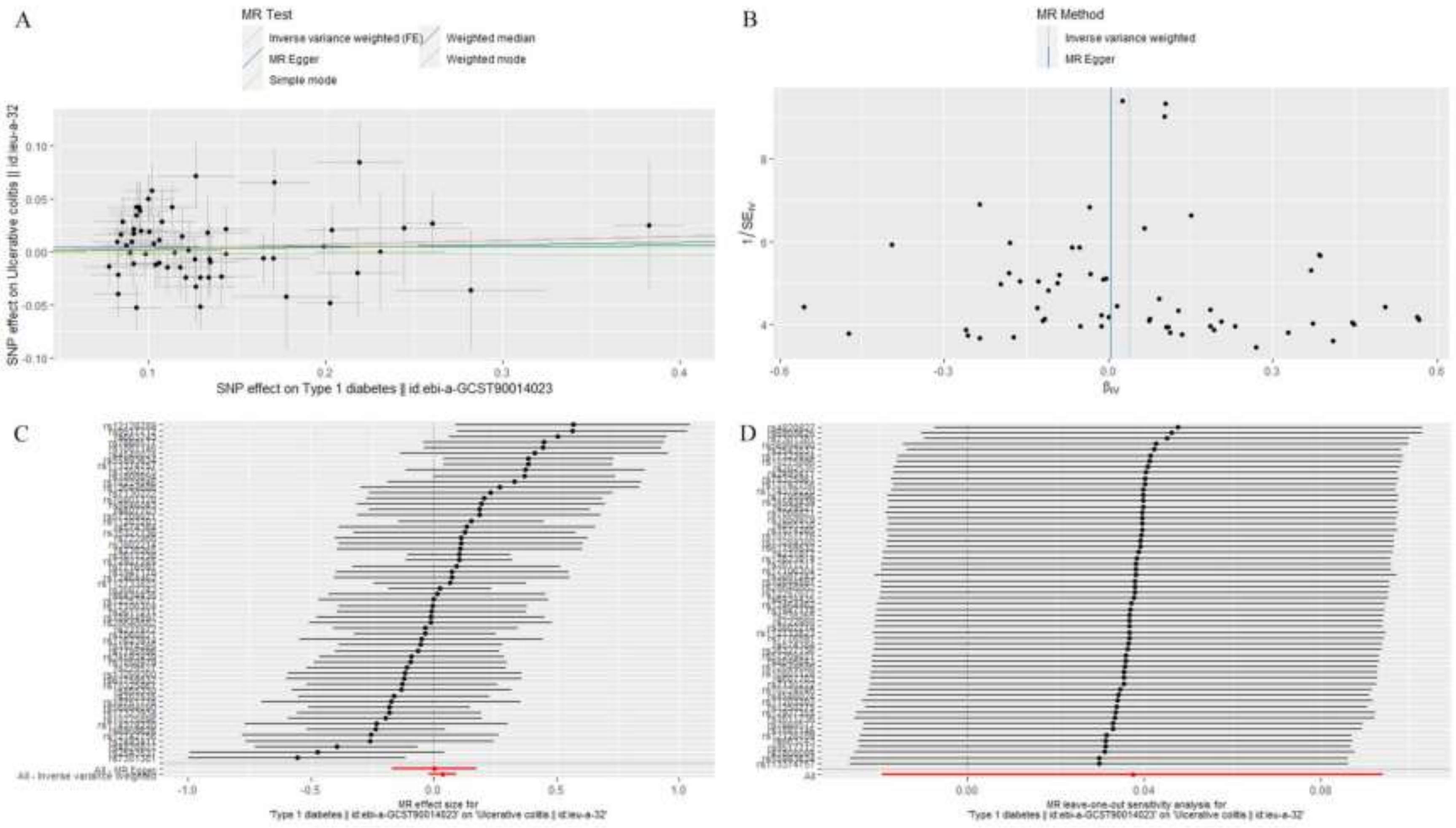

Fig. S7 Scatter plot (A), funnel plot (B), forest plot (C) and leave-one-out analysis (D) of the causal effect of type 1 diabetes mellitus on ulcerative colitis.

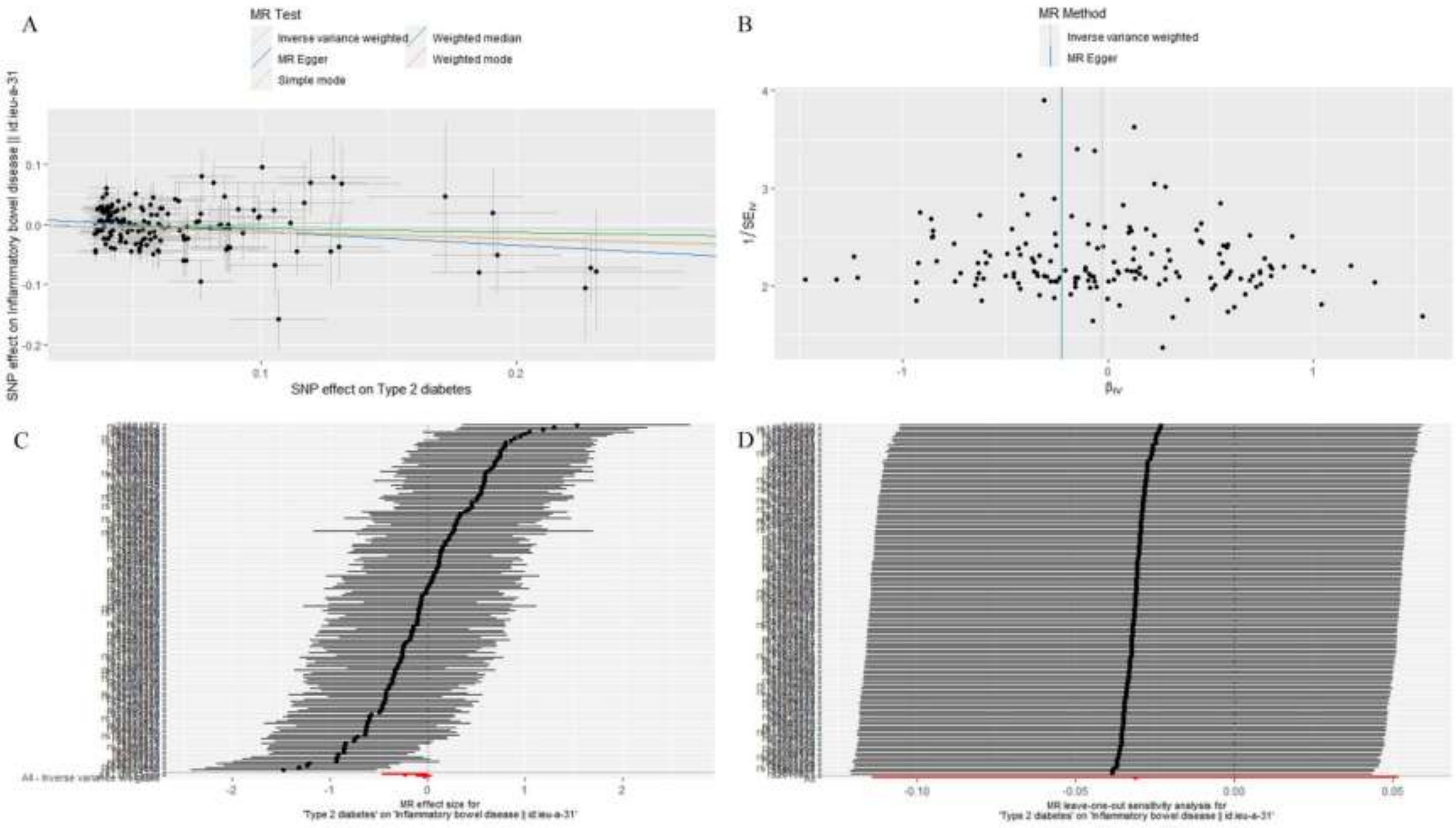

Fig. S8 Scatter plot (A), funnel plot (B), forest plot (C) and leave-one-out analysis (D) of the causal effect of type 2 diabetes mellitus on inflammatory bowel disease.

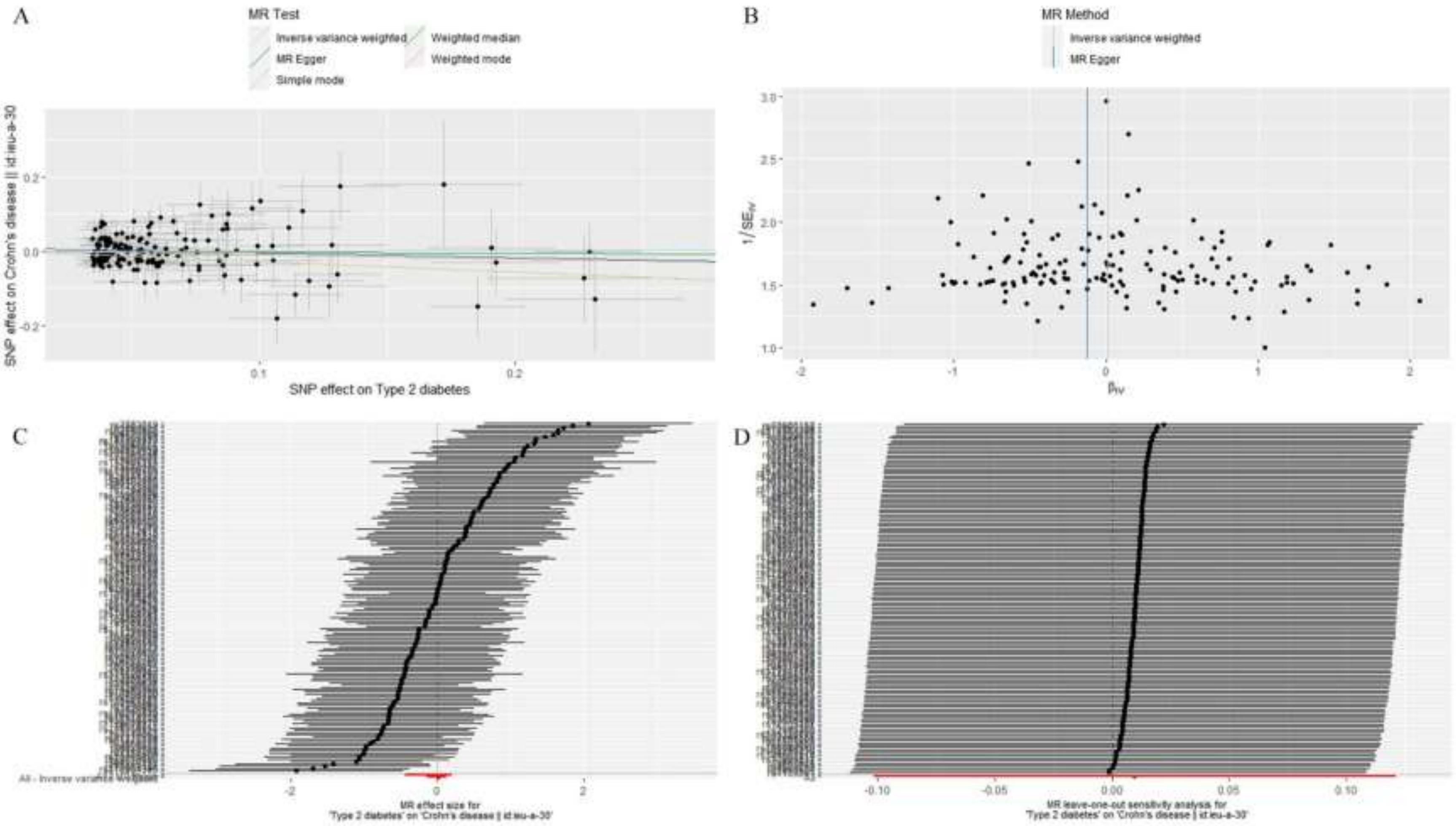

Fig. S9 Scatter plot (A), funnel plot (B), forest plot (C) and leave-one-out analysis (D) of the causal effect of type 2 diabetes mellitus on Crohn's disease.

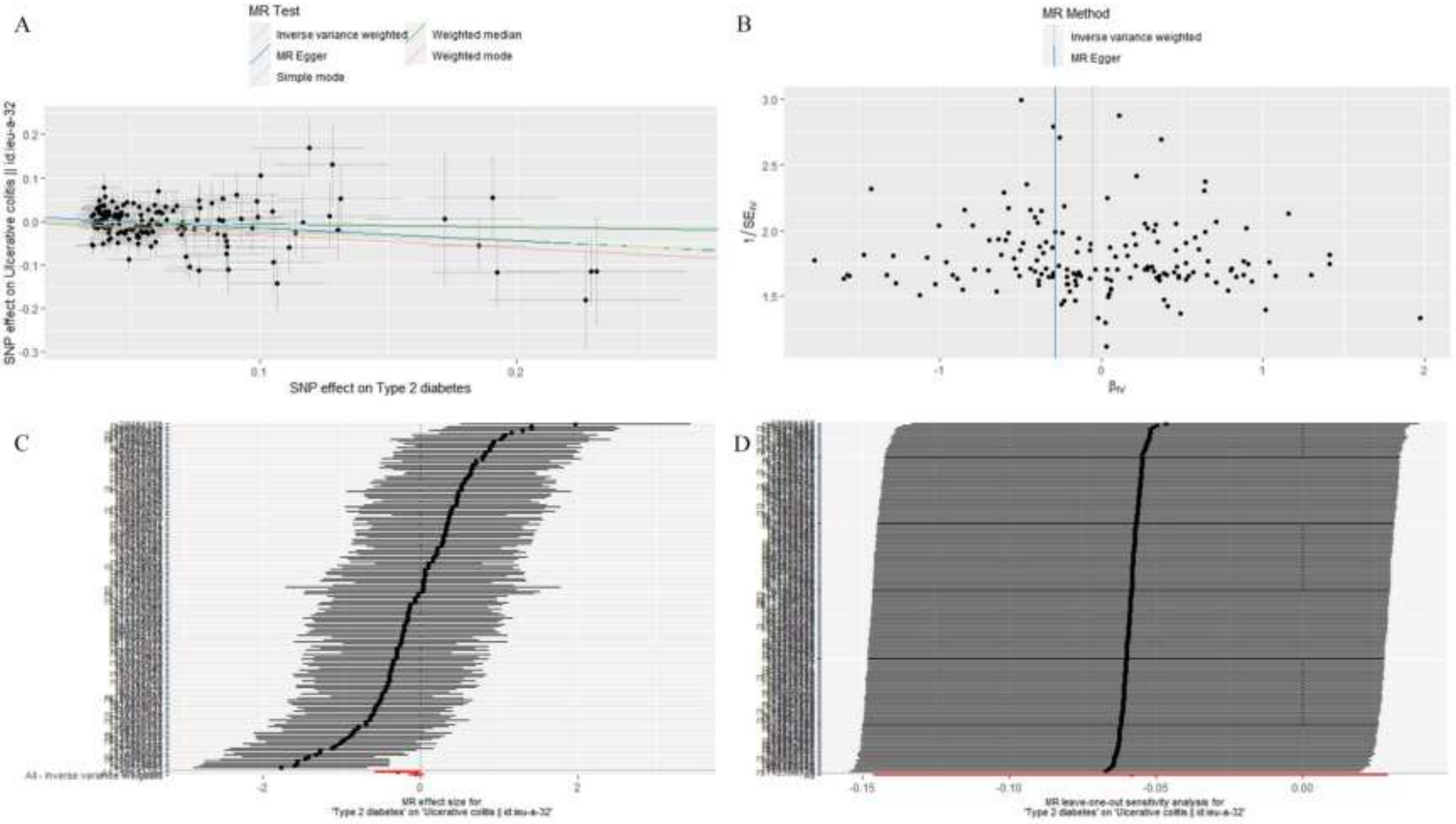

Fig. S10 Scatter plot (A), funnel plot (B), forest plot (C) and leave-one-out analysis (D) of the causal effect of type 2 diabetes mellitus on ulcerative colitis.

Table S1 Removed pleiotropic SNPs associated with IBD, UC, CD and T1DM, T2DM

Exposure	Outcome	F statistic < 10	PhenoScanner database	MR-PRESSO outlier test	Palindromic SNP	leave-one-out analysis
IBD	T1DM	NA	rs112694524, rs1886731, rs1873625, rs12446550	rs1003342, rs11236797, rs1250573, rs12936409, rs142770866, rs148844907, rs2542147, rs3024493, rs35260072, rs6927172, rs9272514	NA	NA
	T2DM	NA	rs112694524, rs1886731, rs1873625, rs12446550	rs1250573, rs4077515, rs4712528, rs6062496, rs11677953, rs142770866, rs56062135, rs2241878, rs744166	NA	NA
UC	T1DM	NA	rs3024493, rs1886731, rs9823546, rs7523335, rs798502, rs3829111, rs148844907	rs7911680, rs2212434, rs6933404, rs9272514	rs9891174	NA

	T2DM	NA	rs3024493, rs1886731, rs9823546, rs7523335, rs798502, rs3829111, rs148844907	rs6062496, rs137845	rs9891174	NA
CD	T1DM	NA	rs3024505, rs78487399, rs13135092, rs114607072, rs7543234, rs1873625, rs147684209	rs11236797, rs1250573, rs148844907, rs2188962, rs281379, rs4902642, rs80262450, rs907092	rs12692254	NA
	T2DM	NA	rs3024505, rs78487399, rs13135092, rs114607072, rs7543234, rs1873625, rs147684209	rs1056441, rs12194825, rs1250573, rs4077515, rs56062135, rs744166	rs12692254	NA

Abbreviations: SNPs= single-nucleotide polymorphisms, MR-PRESSO= MR Pleiotropy RESidual Sum and Outlier, NA= not available, IBD= inflammatory bowel disease, UC= ulcerative colitis, CD= Crohn’s disease, T1DM= type 1 diabetes mellitus, T2DM= type 2 diabetes mellitus.

Table S2 Removed pleiotropic SNPs associated with T1DM and IBD, UC and CD.

Exposure	Outcome	F statistic < 10	PhenoScanner database	MR-PRESSO outlier test	Palindromic SNP	leave-one-out analysis
T1DM	IBD	NA	rs6679677, rs34536443, rs601338, rs61839660, rs3024493, rs2111485, rs2188962, rs7936434, rs3184504, rs7237497, rs13147049, rs55893453, rs1701704, rs9385401, rs9468618, rs6908236, rs73432769, rs41295159	rs4820827, rs663743, rs689, rs6908626, rs7301381, rs7511678, rs9517712	rs2303137	NA
	UC	NA	rs6679677, rs34536443, rs601338, rs61839660, rs3024493, rs2111485, rs2188962, rs7936434, rs3184504, rs7237497, rs13147049, rs55893453, rs1701704, rs9385401, rs9468618, rs6908236,	rs7511678, rs4490209	rs2303137	rs689

		rs73432769, rs41295159			
CD	NA	rs6679677, rs34536443, rs601338, rs61839660, rs3024493, rs2111485, rs2188962, rs7936434, rs3184504, rs7237497, rs13147049, rs55893453, rs1701704, rs9385401, rs9468618, rs6908236, rs73432769, rs41295159	rs10275896, rs10844597, rs17106304, rs17323934, rs238265, rs2493411, rs663743, rs7668577, rs10275896, rs6908626, rs4820827	rs2303137	rs689

Abbreviations: SNPs= single-nucleotide polymorphisms, MR-PRESSO= MR Pleiotropy RESidual Sum and Outlier, NA= not available, IBD= inflammatory bowel disease, UC= ulcerative colitis, CD= Crohn’s disease, T1DM= type 1 diabetes mellitus.

Table S3 Removed pleiotropic SNPs associated with T2DM and IBD, UC and CD.

Exposure	Outcome	F statistic < 10	PhenoScanner database	MR-PRESSO outlier test	Palindromic SNP	leave-one-out analysis
T2DM	IBD	NA	rs1728918, rs1516971, rs6544646, rs329118, rs12577418, rs62271373, rs2851447, rs7546735, rs12140153, rs34632838, rs62106252, rs2867105, rs2862874, rs10181181, rs6446298, rs6792607, rs114649518, rs4488965, rs4324525, rs1544755, rs34298980, rs2744475, rs2980423, rs67763258, rs1483582, rs12001437, rs35521277, rs12803915, rs12583517, rs2143975, rs17597295, rs3825566, rs4776970, rs8054556, rs7206010, rs12596054, rs12924872, rs869492, rs4260092, rs9957145, rs12454712, rs12972156, rs4911403, rs1632870, rs9267954, rs12920022	rs13267392, rs60980157	rs10963924, rs12143910, rs12617964, rs12900395, rs1999536, rs4083914, rs4237150	NA
	UC	NA	rs1728918, rs1516971, rs6544646, rs329118, rs12577418, rs62271373, rs2851447, rs7546735, rs12140153, rs34632838, rs62106252, rs2867105, rs2862874, rs10181181,	NA	rs10963924, rs12143910,	NA

		rs6446298, rs6792607, rs114649518, rs4488965, rs4324525, rs1544755, rs34298980, rs2744475, rs2980423, rs67763258, rs1483582, rs12001437, rs35521277, rs12803915, rs12583517, rs2143975, rs17597295, rs3825566, rs4776970, rs8054556, rs7206010, rs12596054, rs12924872, rs869492, rs4260092, rs9957145, rs12454712, rs12972156, rs4911403, rs1632870, rs9267954, rs12920022		rs12617964, rs12900395, rs1999536, rs4083914, rs4237150	
CD	NA	rs1728918, rs1516971, rs6544646, rs329118, rs12577418, rs62271373, rs2851447, rs7546735, rs1214015, rs34632838, rs62106252, rs2867105, rs2862874, rs10181181, rs6446298, rs6792607, rs114649518, rs4488965, rs4324525, rs1544755, rs34298980, rs2744475, rs2980423, rs67763258, rs1483582, rs12001437, rs35521277, rs12803915, rs12583517, rs2143975, rs17597295, rs3825566, rs4776970, rs8054556, rs7206010, rs12596054, rs12924872, rs869492, rs4260092, rs9957145, rs12454712, rs12972156, rs4911403, rs1632870, rs9267954, rs12920022	rs2793823, rs4422335, rs8032939, rs13267392	rs10963924, rs12143910, rs12617964, rs12900395, rs1999536, rs4083914, rs4237150	NA

Abbreviations: SNPs= single-nucleotide polymorphisms, MR-PRESSO= MR Pleiotropy RESidual Sum and Outlier, NA= not available, T2DM= type 2 diabetes mellitus, IBD= inflammatory bowel disease, UC= ulcerative colitis, CD= Crohn's disease.

Table S4. Characteristics of the SNPs related to IBD and T1DM

SNP	Chr	Position	EA	OA	Exposure: IBD				Outcome: T1DM				F statistic
					EAF	β	SE	*P*	EAF	β	SE	*P*	
rs10045431	5	158814533	C	A	0.717524	0.177406	0.0189	6.58567E-21	0.717	-0.01751	0.015332	0.253	88.10752453
rs10408351	19	33754044	A	G	0.238435	0.137804	0.0221	4.23204E-10	0.213	-0.00477	0.018063	0.792	38.88114989
rs10737481	1	20171514	G	T	0.554896	0.141103	0.017	1.18713E-16	0.531	0.017146	0.014016	0.221	68.89292944
rs10761659	10	64445564	G	A	0.554011	0.161896	0.0172	4.07193E-21	0.537	-0.01331	0.013942	0.34	88.59625073
rs10800314	1	161472789	A	C	0.649219	-0.143097	0.0179	1.17409E-15	0.657	-0.003811	0.014855	0.798	63.90796607
rs11209026	1	67705958	A	G	0.0541886	-0.72629	0.0422	1.76319E-66	0.0626	-0.045409	0.02865	0.113	296.2069384
rs112401990	2	61199327	A	G	0.375383	0.142202	0.0174	2.83923E-16	0.341	0.033237	0.015573	0.0327997	66.79022593
rs113991740	17	32625383	A	T	0.271565	-0.107697	0.0189	1.31999E-08	0.293	0.019582	0.030274	0.518	32.47009829
rs11548656	16	81916912	G	A	0.034026	-0.292796	0.0507	7.71792E-09	0.0384	-0.012046	0.040899	0.768	33.35142234
rs11677953	2	219121663	A	G	0.415063	0.0975985	0.0171	1.053E-08	0.382	-0.038423	0.014355	0.00743995	32.57572314

rs12764283	10	35530460	A	G	0.337409	0.126597	0.0179	1.5729E-12	0.337	0.018796	0.014781	0.203	50.01966358
rs131657	22	21917550	A	T	0.201666	0.136496	0.0212	1.237E-10	0.191	0.121786	0.047055	0.00965006	41.45416077
rs13178036	5	40218529	C	G	0.341807	0.100904	0.0184	4.12895E-08	0.33	0.01715	0.015062	0.255	30.07330227
rs140892874	12	40824798	C	T	0.027461	0.409594	0.0512	1.2841E-15	0.0185	0.037779	0.052776	0.474	63.99812501
rs1551399	8	126539965	C	A	0.615807	0.101302	0.0173	5.01199E-09	0.618	0.00816	0.015198	0.590999	34.28813259
rs1736161	21	16833222	A	G	0.428732	-0.123298	0.0174	1.3369E-12	0.434	-0.023853	0.014313	0.0956005	50.21269918
rs17800987	5	150323428	G	A	0.089179	0.2017	0.0305	3.70681E-11	0.085	0.016901	0.024997	0.499001	43.73328675
rs1887428	9	4984530	C	G	0.61936	-0.171595	0.0178	6.64814E-22	0.623	-0.026983	0.014613	0.0648007	92.93284947
rs2076756	16	50756881	G	A	0.27064	0.187595	0.0186	5.59114E-24	0.23	0.004466	0.016275	0.784	101.7224073
rs2193041	12	68502110	G	A	0.388781	0.133703	0.0172	6.90876E-15	0.376	-0.01469	0.014438	0.309	60.42621758
rs2241878	2	234183718	C	T	0.538498	0.148001	0.0169	1.74502E-18	0.505	0.026369	0.014016	0.0599004	76.69302896
rs254560	5	134443606	A	G	0.400063	0.0995014	0.0171	6.159E-09	0.407	-0.031062	0.014166	0.0283002	33.858379
rs2836882	21	40466570	A	G	0.257617	-0.196295	0.0201	1.49005E-22	0.263	-0.012977	0.01601	0.418	95.37320122

rs28383456	6	32609453	T	C	0.345334	-0.177501	0.02	5.76899E-19	0.0196	0.236976	0.136187	0.0817994	78.7665125
rs34190331	6	111840820	A	G	0.0853045	0.176899	0.0303	5.39399E-09	0.0626	0.054217	0.028307	0.0554996	34.0851727
rs35730213	1	200874229	C	G	0.262591	-0.151404	0.0194	6.90717E-15	0.277	-0.040134	0.015557	0.00989008	60.90756514
rs3850378	14	88417517	C	T	0.096461	0.155099	0.0282	3.80102E-08	0.0969	0.011335	0.025158	0.652001	30.24960993
rs4077515	9	139266496	T	C	0.424242	0.179401	0.0172	1.50003E-25	0.427	-0.005208	0.014299	0.716	108.790964
rs4246905	9	117553249	C	T	0.729896	0.163001	0.0197	1.41514E-16	0.729	0.046456	0.015567	0.00284001	68.46176403
rs444210	6	167390242	G	A	0.545781	0.109502	0.0168	7.39095E-11	0.531	-0.021844	0.013969	0.118	42.48401362
rs45528737	4	123275555	T	C	0.111833	0.166802	0.03	2.65901E-08	0.0999	0.061574	0.025003	0.0138	30.91434134
rs4676408	2	241574401	A	G	0.515063	0.118103	0.0181	6.61912E-11	0.545	-0.02842	0.014186	0.0451004	42.5759855
rs4712528	6	20678430	C	G	0.787692	0.122598	0.0207	3.07397E-09	0.771	0.019368	0.016774	0.248	35.07729376
rs4730272	7	107478227	G	A	0.512591	-0.134102	0.0178	4.50194E-14	0.52	0.002063	0.014128	0.884	56.75844718
rs4851586	2	103064264	C	T	0.75745	-0.122403	0.0193	2.32498E-10	0.783	0.029787	0.016815	0.0764997	40.2225413
rs56062135	15	67455630	T	C	0.236922	0.1509	0.0198	2.63998E-14	0.243	0.038671	0.016332	0.0178999	58.0828742

rs6062496	20	62329099	A	G	0.578011	0.164997	0.018	5.47898E-20	0.566	0.022397	0.014372	0.119	84.02472225
rs6584283	10	101290301	C	T	0.510643	-0.180303	0.0169	1.70098E-26	0.534	-0.038332	0.01402	0.00625	113.823647
rs6826501	4	36076676	T	C	0.536078	-0.0927953	0.0169	4.12098E-08	0.501	0.01628	0.014076	0.247	30.14939149
rs6873866	5	96247810	C	T	0.528821	-0.106996	0.0176	1.085E-09	0.562	-0.02038	0.014958	0.173	36.95810956
rs6880778	5	40399096	G	A	0.620729	0.187801	0.0173	2.14388E-27	0.597	-0.018863	0.014247	0.186	117.842947
rs6911490	6	106522027	C	T	0.778565	-0.142801	0.0208	6.82025E-12	0.787	0.011457	0.017315	0.508	47.13416605
rs72798422	16	50866917	C	T	0.041718	0.2776	0.0431	1.18899E-10	0.0333	-0.050813	0.038737	0.19	41.48435893
rs7285952	22	39733096	G	T	0.157193	-0.176002	0.0235	7.60151E-14	0.15	0.023649	0.019242	0.219	56.0918135
rs744166	17	40514201	G	A	0.409963	-0.120703	0.0172	2.15675E-12	0.425	-0.014848	0.014037	0.29	49.24693824
rs7523335	1	8180210	A	G	0.173424	-0.140504	0.0225	4.15796E-10	0.204	0.001733	0.01767	0.922	38.9953067
rs8134436	21	45616497	G	C	0.603989	-0.144698	0.017	2.0179E-17	0.608	-0.044128	0.037812	0.243	72.44813565
rs9370774	6	14721897	C	T	0.20068	-0.130704	0.0219	2.536E-09	0.176	0.008515	0.018592	0.647	35.61964016
rs9934775	16	50383077	T	C	0.16068	-0.139595	0.0232	1.71498E-09	0.156	-0.055534	0.019632	0.00467003	36.20460022

Abbreviation: IBD= inflammatory bowel disease, T1DM= type 1 diabetes mellitus, SNPs= single-nucleotide polymorphisms, Chr= Chromosome, EA= Effect Allele, OA= Other

Allele, EAF= effect allele frequency, SE= standard error.

Table S5. Characteristics of the SNPs related to IBD and T2DM

SNP	Chr	Position	EA	OA	Exposure: IBD				Outcome: T2DM				F statistic
					EAF	β	SE	*P*	EAF	β	SE	*P*	
rs1003342	22	30570022	G	A	0.531334	-0.095001	0.0168	1.67101E-08	0.5439	0.0026	0.0064	0.6839	31.97700539
rs10045431	5	158814533	C	A	0.717524	0.177406	0.0189	6.58567E-21	0.7116	0.001	0.007	0.8866	88.10752453
rs10408351	19	33754044	A	G	0.238435	0.137804	0.0221	4.23204E-10	0.2259	0.0073	0.0079	0.3526	38.88114989
rs10737481	1	20171514	G	T	0.554896	0.141103	0.017	1.18713E-16	0.5292	0.0099	0.0064	0.1211	68.89292944
rs10761659	10	64445564	G	A	0.554011	0.161896	0.0172	4.07193E-21	0.5399	0.008	0.0064	0.2104	88.59625073
rs11209026	1	67705958	A	G	0.0541886	-0.72629	0.0422	1.76319E-66	0.0637	0.001	0.0131	0.9391	296.2069384
rs11236797	11	76299649	A	C	0.470011	0.155704	0.017	4.75007E-20	0.4425	-0.0117	0.0065	0.07149	83.88835853
rs112401990	2	61199327	A	G	0.375383	0.142202	0.0174	2.83923E-16	0.3442	0.0121	0.0067	0.07096	66.79022593
rs11548656	16	81916912	G	A	0.034026	-0.292796	0.0507	7.71792E-09	0.0356	0.0145	0.0178	0.4153	33.35142234
rs12764283	10	35530460	A	G	0.337409	0.126597	0.0179	1.5729E-12	0.3349	0.0099	0.0067	0.1396	50.01966358

rs12936409	17	38043649	T	C	0.476383	0.145701	0.0168	3.86812E-18	0.4703	-0.0079	0.0064	0.2161	75.2153536
rs13178036	5	40218529	C	G	0.341807	0.100904	0.0184	4.12895E-08	0.3358	0.0005	0.0068	0.9414	30.07330227
rs140892874	12	40824798	C	T	0.027461	0.409594	0.0512	1.2841E-15	0.0208	0.0411	0.023	0.07438	63.99812501
rs148844907	6	31628397	A	T	0.0103903	1.1375	0.0963	3.62577E-32	0.0137	0.0694	0.0313	0.02663	139.5244234
rs1551399	8	126539965	C	A	0.615807	0.101302	0.0173	5.01199E-09	0.6219	-0.0003	0.0068	0.9648	34.28813259
rs1736161	21	16833222	A	G	0.428732	-0.123298	0.0174	1.3369E-12	0.4361	0.0063	0.0065	0.3318	50.21269918
rs17800987	5	150323428	G	A	0.089179	0.2017	0.0305	3.70681E-11	0.0831	-0.0076	0.0118	0.5206	43.73328675
rs1887428	9	4984530	C	G	0.61936	-0.171595	0.0178	6.64814E-22	0.6179	0.0089	0.0066	0.1773	92.93284947
rs2076756	16	50756881	G	A	0.27064	0.187595	0.0186	5.59114E-24	0.2448	-0.011	0.0074	0.1389	101.7224073
rs2193041	12	68502110	G	A	0.388781	0.133703	0.0172	6.90876E-15	0.3844	-0.0097	0.0066	0.1414	60.42621758
rs2542147	18	12775851	T	G	0.837193	-0.1513	0.0227	2.78484E-11	0.8364	-0.016	0.0091	0.07901	44.42486755
rs254560	5	134443606	A	G	0.400063	0.0995014	0.0171	6.159E-09	0.414	-0.0095	0.0065	0.1433	33.858379
rs2836882	21	40466570	A	G	0.257617	-0.196295	0.0201	1.49005E-22	0.2724	0.0057	0.0072	0.4301	95.37320122

rs3024493	1	206943968	A	C	0.169666	0.212996	0.0222	8.47618E-22	0.1529	-0.0065	0.0089	0.4652	92.05278796
rs34190331	6	111840820	A	G	0.0853045	0.176899	0.0303	5.39399E-09	0.0661	-0.0243	0.013	0.06125	34.0851727
rs35260072	5	131630852	C	A	0.433153	0.142197	0.017	7.06643E-17	0.4139	-0.0089	0.0065	0.1704	69.96535228
rs35730213	1	200874229	C	G	0.262591	-0.151404	0.0194	6.90717E-15	0.2839	0.0033	0.0071	0.643	60.90756514
rs3850378	14	88417517	C	T	0.096461	0.155099	0.0282	3.80102E-08	0.0933	0.0154	0.0114	0.1772	30.24960993
rs4246905	9	117553249	C	T	0.729896	0.163001	0.0197	1.41514E-16	0.7197	-0.0005	0.0074	0.9464	68.46176403
rs444210	6	167390242	G	A	0.545781	0.109502	0.0168	7.39095E-11	0.5472	0.0155	0.0064	0.01523	42.48401362
rs45528737	4	123275555	T	C	0.111833	0.166802	0.03	2.65901E-08	0.119	-0.0048	0.0105	0.6466	30.91434134
rs4676408	2	241574401	A	G	0.515063	0.118103	0.0181	6.61912E-11	0.5232	-0.0046	0.0068	0.4991	42.5759855
rs4730272	7	107478227	G	A	0.512591	-0.134102	0.0178	4.50194E-14	0.52	-0.0167	0.0065	0.01009	56.75844718
rs4851586	2	103064264	C	T	0.75745	-0.122403	0.0193	2.32498E-10	0.7723	-0.0122	0.0076	0.1104	40.2225413
rs6584283	10	101290301	C	T	0.510643	-0.180303	0.0169	1.70098E-26	0.5313	0.0083	0.0064	0.1938	113.823647
rs6826501	4	36076676	T	C	0.536078	-0.0927953	0.0169	4.12098E-08	0.5163	-0.0033	0.0065	0.6112	30.14939149

rs6880778	5	40399096	G	A	0.620729	0.187801	0.0173	2.14388E-27	0.606	-0.0023	0.0065	0.7231	117.842947
rs6911490	6	106522027	C	T	0.778565	-0.142801	0.0208	6.82025E-12	0.7865	-0.0143	0.0079	0.0686	47.13416605
rs6927172	6	138002175	G	C	0.21432	0.110295	0.0202	4.64505E-08	0.2083	-0.0081	0.0079	0.3023	29.8132218
rs72798422	16	50866917	C	T	0.041718	0.2776	0.0431	1.18899E-10	0.0368	0.0064	0.0172	0.7094	41.48435893
rs7285952	22	39733096	G	T	0.157193	-0.176002	0.0235	7.60151E-14	0.1569	-0.0093	0.0088	0.2903	56.0918135
rs7523335	1	8180210	A	G	0.173424	-0.140504	0.0225	4.15796E-10	0.1935	-0.0204	0.0082	0.01249	38.9953067
rs8134436	21	45616497	G	C	0.603989	-0.144698	0.017	2.0179E-17	0.6101	-0.0056	0.007	0.4247	72.44813565
rs9370774	6	14721897	C	T	0.20068	-0.130704	0.0219	2.536E-09	0.1926	-0.0069	0.0083	0.4042	35.61964016
rs9934775	16	50383077	T	C	0.16068	-0.139595	0.0232	1.71498E-09	0.1644	-0.002	0.0086	0.8158	36.20460022

Abbreviation: IBD= inflammatory bowel disease, T2DM= type 2 diabetes mellitus, SNPs= single-nucleotide polymorphisms, Chr= Chromosome, EA= Effect Allele, OA= Other Allele, EAF= effect allele frequency, SE= standard error.

Table S6. Characteristics of the SNPs related to UC and T1DM

SNP	Chr	Position	EA	OA	Exposure: UC				Outcome: T1DM				F statistic
					EAF	β	SE	*P*	EAF	β	SE	*P*	
rs10182512	2	61189469	A	G	0.34989	0.1608	0.0223	5.19398E-13	0.345	0.033422	0.014627	0.0222998	51.99509341
rs10272963	7	107486902	T	C	0.426078	-0.171904	0.0216	1.69083E-15	0.439	0.007025	0.014089	0.617999	63.33801701
rs10737481	1	20171514	G	T	0.556463	0.250104	0.0216	4.36918E-31	0.531	0.017146	0.014016	0.221	134.0706679
rs10917545	1	20128177	A	G	0.882174	-0.185101	0.0335	3.29397E-08	0.889	-0.031078	0.02504	0.215	30.53007815
rs11209026	1	67705958	A	G	0.0589321	-0.561698	0.0517	1.58016E-27	0.0626	-0.045409	0.02865	0.113	118.0387682
rs114152040	5	40444986	A	G	0.0317179	0.339603	0.0623	4.94903E-08	0.0317	0.039804	0.039473	0.313	29.71439846
rs12612675	2	219133137	G	A	0.402826	0.122902	0.0219	1.97702E-08	0.382	-0.037724	0.014365	0.00863993	31.49413399
rs12817473	12	68497408	G	A	0.382176	0.190701	0.0217	1.70883E-18	0.374	-0.016892	0.014487	0.244	77.23007794
rs1359946	13	27536972	A	G	0.196096	0.158302	0.0269	3.83796E-09	0.178	0.004655	0.01818	0.798	34.63125607
rs137845	22	50439430	G	A	0.514874	0.118198	0.0212	2.37602E-08	0.51	-0.024477	0.014937	0.101	31.08483269

rs1801274	1	161479745	G	A	0.483094	-0.182896	0.0217	3.77833E-17	0.526	-0.006344	0.014081	0.652001	71.03770905
rs1887428	9	4984530	C	G	0.622856	-0.176701	0.0224	3.35506E-15	0.623	-0.026983	0.014613	0.0648007	62.22744619
rs254559	5	134444982	A	C	0.404112	0.124304	0.0215	7.62799E-09	0.404	-0.033004	0.014496	0.0227997	33.42668343
rs28383456	6	32609453	T	C	0.334505	-0.337307	0.0256	1.073E-39	0.0196	0.236976	0.136187	0.0817994	173.6084171
rs35730213	1	200874229	C	G	0.266634	-0.167	0.0245	8.81455E-12	0.277	-0.040134	0.015557	0.00989008	46.46230737
rs4574921	9	117538334	T	C	0.740842	0.150599	0.0256	4.23897E-09	0.75	0.040931	0.016033	0.0106999	34.60702332
rs4676410	2	241563739	A	G	0.197128	0.207802	0.0284	2.45923E-13	0.223	-0.030556	0.017165	0.0751	53.53807678
rs483905	11	96023427	A	G	0.294334	0.128903	0.0228	1.57199E-08	0.279	0.00032	0.015392	0.983	31.96364922
rs484356	11	114406639	G	C	0.32765	-0.134199	0.0228	3.95003E-09	0.343	0.030045	0.01471	0.0410998	34.64406664
rs56167332	5	158827769	A	C	0.34162	0.151596	0.0231	5.29907E-11	0.337	-0.000448	0.014978	0.976	43.06768467
rs6017342	20	43065028	C	A	0.537652	0.191306	0.024	1.38484E-15	0.524	0.009758	0.014303	0.495	63.53816951
rs6062496	20	62329099	A	G	0.572636	0.158498	0.0224	1.46791E-12	0.566	0.022397	0.014372	0.119	50.06699618
rs7282490	21	45615741	A	G	0.603618	-0.139699	0.0214	7.0762E-11	0.605	-0.04743	0.037742	0.209	42.61466198

rs7752873	6	106579332	T	C	0.13681	0.182297	0.0303	1.83299E-09	0.132	-0.028849	0.020571	0.161	36.19710073
rs989960	7	107445727	T	C	0.424904	-0.129096	0.0215	1.766E-09	0.441	-0.011328	0.014261	0.427	36.05360133
rs9977672	21	40463283	A	G	0.251316	-0.245006	0.0261	6.20583E-21	0.254	-0.010088	0.01624	0.534	88.11958139

Abbreviation: UC= ulcerative colitis, T1DM= type 1 diabetes mellitus, SNPs= single-nucleotide polymorphisms, Chr= Chromosome, EA= Effect Allele, OA= Other Allele, EAF= effect allele frequency, SE= standard error.

Table S7. Characteristics of the SNPs related to UC and T2DM

SNP	Chr	Position	EA	OA	Exposure: UC				Outcome: T2DM				F statistic
					EAF	β	SE	*P*	EAF	β	SE	*P*	
rs10182512	2	61189469	A	G	0.34989	0.1608	0.0223	5.19398E-13	0.3467	0.0094	0.0067	0.1607	51.99509341
rs10272963	7	107486902	T	C	0.426078	-0.171904	0.0216	1.69083E-15	0.442	-0.0103	0.0064	0.1068	63.33801701
rs10737481	1	20171514	G	T	0.556463	0.250104	0.0216	4.36918E-31	0.5292	0.0099	0.0064	0.1211	134.0706679
rs10917545	1	20128177	A	G	0.882174	-0.185101	0.0335	3.29397E-08	0.8951	-0.0042	0.0107	0.6941	30.53007815
rs11209026	1	67705958	A	G	0.0589321	-0.561698	0.0517	1.58016E-27	0.0637	0.001	0.0131	0.9391	118.0387682
rs114152040	5	40444986	A	G	0.0317179	0.339603	0.0623	4.94903E-08	0.034	-0.0006	0.0179	0.9733	29.71439846
rs12612675	2	219133137	G	A	0.402826	0.122902	0.0219	1.97702E-08	0.4008	0.0213	0.0069	0.002054	31.49413399
rs12817473	12	68497408	G	A	0.382176	0.190701	0.0217	1.70883E-18	0.3806	-0.01	0.0066	0.1295	77.23007794
rs1359946	13	27536972	A	G	0.196096	0.158302	0.0269	3.83796E-09	0.1778	0.0021	0.0085	0.8044	34.63125607
rs1801274	1	161479745	G	A	0.483094	-0.182896	0.0217	3.77833E-17	0.5152	0.0038	0.0065	0.5583	71.03770905

rs1887428	9	4984530	C	G	0.622856	-0.176701	0.0224	3.35506E-15	0.6179	0.0089	0.0066	0.1773	62.22744619
rs2212434	11	76281593	T	C	0.459604	0.141899	0.0213	2.4632E-11	0.4406	-0.0118	0.0066	0.07363	44.38124314
rs254559	5	134444982	A	C	0.404112	0.124304	0.0215	7.62799E-09	0.4111	-0.0093	0.0065	0.152	33.42668343
rs35730213	1	200874229	C	G	0.266634	-0.167	0.0245	8.81455E-12	0.2839	0.0033	0.0071	0.643	46.46230737
rs4574921	9	117538334	T	C	0.740842	0.150599	0.0256	4.23897E-09	0.745	-0.0009	0.0073	0.9023	34.60702332
rs4676410	2	241563739	A	G	0.197128	0.207802	0.0284	2.45923E-13	0.2113	-0.0078	0.0081	0.3333	53.53807678
rs483905	11	96023427	A	G	0.294334	0.128903	0.0228	1.57199E-08	0.2879	-0.0049	0.0071	0.4913	31.96364922
rs56167332	5	158827769	A	C	0.34162	0.151596	0.0231	5.29907E-11	0.3423	-0.0095	0.0068	0.1627	43.06768467
rs6017342	20	43065028	C	A	0.537652	0.191306	0.024	1.38484E-15	0.5246	-0.0043	0.0065	0.5077	63.53816951
rs6933404	6	137959235	C	T	0.21562	0.166799	0.0252	3.68468E-11	0.2091	-0.0089	0.0079	0.2571	43.81126606
rs7282490	21	45615741	A	G	0.603618	-0.139699	0.0214	7.0762E-11	0.6067	-0.0056	0.007	0.4247	42.61466198
rs7752873	6	106579332	T	C	0.13681	0.182297	0.0303	1.83299E-09	0.1346	0.0121	0.0094	0.1991	36.19710073
rs7911680	10	101293468	C	A	0.490094	-0.1718	0.0213	8.26609E-16	0.5143	0.0087	0.0064	0.1731	65.05596332

rs989960	7	107445727	T	C	0.424904	-0.129096	0.0215	1.766E-09	0.4397	0.0021	0.0064	0.7423	36.05360133
rs9977672	21	40463283	A	G	0.251316	-0.245006	0.0261	6.20583E-21	0.2643	0.0043	0.0073	0.5574	88.11958139

Abbreviation: UC= ulcerative colitis, T2DM= type 2 diabetes mellitus, SNPs= single-nucleotide polymorphisms, Chr= Chromosome, EA= Effect Allele, OA= Other Allele, EAF= effect allele frequency, SE= standard error.

Table S8. Characteristics of the SNPs related to CD and T1DM

SNP	Chr	Position	EA	OA	Exposure: CD				Outcome: T1DM				F statistic
					EAF	β	SE	*P*	EAF	β	SE	*P*	
rs1056441	20	62370349	C	T	0.697697	0.167	0.0255	5.44127E-11	0.689	0.02712	0.015244	0.0752004	42.88965782
rs10761659	10	64445564	G	A	0.55269	0.212006	0.0237	3.41979E-19	0.537	-0.01331	0.013942	0.34	80.02019626
rs11209026	1	67705958	A	G	0.0563213	-0.995199	0.0639	1.05196E-54	0.0626	-0.045409	0.02865	0.113	242.5594201
rs112401990	2	61199327	A	G	0.373412	0.132203	0.0237	2.35001E-08	0.341	0.033237	0.015573	0.0327997	31.11615519
rs11564236	12	40828306	T	A	0.034419	0.519093	0.0595	2.84512E-18	0.0186	0.043028	0.0527	0.414	76.11257472
rs12194825	6	20835260	A	T	0.18587	-0.171904	0.0298	0.000000008	0.196	-0.024643	0.017672	0.163	33.27663756
rs12717899	5	141482341	T	G	0.793845	0.159198	0.0289	3.59402E-08	0.794	0.058749	0.017218	0.000645	30.3444681
rs1297271	21	16823163	T	C	0.429877	-0.154901	0.0237	6.27769E-11	0.434	-0.025187	0.014197	0.0759994	42.71808257
rs1332099	10	101298451	C	T	0.515025	-0.211597	0.0231	4.35712E-20	0.537	-0.041984	0.014034	0.00277	83.90639308
rs140054334	6	31587042	T	C	0.038307	0.349797	0.0628	2.56602E-08	0.013	-0.115424	0.058671	0.0490998	31.02507739

rs1456896	7	50304461	T	C	0.697556	0.139301	0.0251	2.89701E-08	0.676	-0.010079	0.01484	0.497	30.8007311
rs147018773	5	150238521	T	C	0.0966978	0.321699	0.0375	8.88792E-18	0.0772	0.038014	0.025823	0.141	73.59306425
rs151314883	22	39735087	A	G	0.157585	-0.223994	0.0327	7.12361E-12	0.15	0.023074	0.019261	0.231	46.92208104
rs1887428	9	4984530	C	G	0.623303	-0.168099	0.0243	4.22377E-12	0.623	-0.026983	0.014613	0.0648007	47.8539413
rs1932990	13	44460242	T	C	0.253841	0.152901	0.0263	6.02102E-09	0.208	-0.029502	0.017029	0.0831994	33.79941274
rs2076756	16	50756881	G	A	0.283672	0.399806	0.0242	3.24489E-61	0.23	0.004466	0.016275	0.784	272.9404372
rs2129944	19	10516198	G	T	0.291299	-0.1562	0.0271	7.80998E-09	0.167	-0.035763	0.016427	0.0294999	33.22182432
rs2505640	10	35459497	G	A	0.643588	-0.145701	0.0237	7.61202E-10	0.648	-0.014556	0.014619	0.319	37.79447988
rs28701841	6	106530330	A	G	0.116563	0.224303	0.0373	1.85102E-09	0.099	-0.01224	0.023386	0.600999	36.16200491
rs3091315	17	32593665	G	A	0.265588	-0.179501	0.0263	9.52138E-12	0.288	-0.008053	0.015444	0.601999	46.58244156
rs3810936	9	117552885	C	T	0.698408	0.207799	0.0263	2.46207E-15	0.7	0.037055	0.015126	0.0143001	62.42742327
rs4077515	9	139266496	T	C	0.419834	0.215901	0.0235	4.37019E-20	0.427	-0.005208	0.014299	0.716	84.40605125
rs444210	6	167390242	G	A	0.546838	0.163402	0.0229	1.02494E-12	0.531	-0.021844	0.013969	0.118	50.91476822

rs4851586	2	103064264	C	T	0.759729	-0.168899	0.0261	9.93574E-11	0.783	0.029787	0.016815	0.0764997	41.87676664
rs4921497	5	158848253	G	C	0.329415	0.160298	0.0244	5.49414E-11	0.328	0.006401	0.014934	0.667999	43.15951492
rs56062135	15	67455630	T	C	0.234271	0.193097	0.0269	7.45247E-13	0.243	0.038671	0.016332	0.0178999	51.52838049
rs6588243	1	67603383	C	A	0.589982	0.131704	0.0234	1.77701E-08	0.56	-0.013949	0.01414	0.324	31.67861717
rs6704109	1	172857050	T	C	0.256123	0.202002	0.0256	2.77013E-15	0.251	0.007611	0.01618	0.638	62.26319581
rs6873866	5	96247810	C	T	0.534873	-0.168096	0.0239	2.0649E-12	0.562	-0.02038	0.014958	0.173	49.4673854
rs697693	1	7886424	A	G	0.201415	0.172296	0.0281	8.35507E-10	0.172	0.006156	0.018137	0.734	37.5956632
rs7276302	21	45615023	G	A	0.608306	-0.171598	0.0231	1.22603E-13	0.606	-0.054114	0.03775	0.152	55.18238714
rs72798422	16	50866917	C	T	0.047701	0.590392	0.0508	3.19301E-31	0.0333	-0.050813	0.038737	0.19	135.0683217
rs744166	17	40514201	G	A	0.408159	-0.129299	0.0233	2.92301E-08	0.425	-0.014848	0.014037	0.29	30.79487815
rs8137950	22	21969640	C	T	0.199556	0.173996	0.0286	1.167E-09	0.284	0.040899	0.019066	0.0318999	37.01233314
rs8178977	19	1106477	C	G	0.238841	0.1928	0.0274	2.05589E-12	0.237	0.007974	0.017274	0.644	49.51228089
rs921720	8	126534671	G	A	0.618841	0.162895	0.0237	6.39588E-12	0.645	0.000979	0.01445	0.946	47.24097104

Abbreviation: CD= Crohn’ s disease, T1DM= type 1 diabetes mellitus, SNPs= single-nucleotide polymorphisms, Chr= Chromosome, EA= Effect Allele, OA= Other Allele, EAF=

effect allele frequency, SE= standard error.

Table S9. Characteristics of the SNPs related to CD and T2DM

SNP	Chr	Position	EA	OA	Exposure: CD				Outcome: T2DM				F statistic
					EAF	β	SE	*P*	EAF	β	SE	*P*	
rs10761659	10	64445564	G	A	0.55269	0.212006	0.0237	3.41979E-19	0.5399	0.008	0.0064	0.2104	80.02019626
rs11209026	1	67705958	A	G	0.0563213	-0.995199	0.0639	1.05196E-54	0.0637	0.001	0.0131	0.9391	242.5594201
rs11236797	11	76299649	A	C	0.473264	0.181104	0.0231	4.854E-15	0.4425	-0.0117	0.0065	0.07149	61.4656
rs112401990	2	61199327	A	G	0.373412	0.132203	0.0237	2.35001E-08	0.3442	0.0121	0.0067	0.07096	31.11615519
rs11564236	12	40828306	T	A	0.034419	0.519093	0.0595	2.84512E-18	0.0208	0.0419	0.023	0.06891	76.11257472
rs12717899	5	141482341	T	G	0.793845	0.159198	0.0289	3.59402E-08	0.7862	-0.001	0.0079	0.8987	30.3444681
rs1297271	21	16823163	T	C	0.429877	-0.154901	0.0237	6.27769E-11	0.4368	0.0047	0.0064	0.4618	42.71808257
rs1332099	10	101298451	C	T	0.515025	-0.211597	0.0231	4.35712E-20	0.535	0.0095	0.0064	0.1369	83.90639308
rs140054334	6	31587042	T	C	0.038307	0.349797	0.0628	2.56602E-08	0.0223	-0.0067	0.0236	0.7761	31.02507739
rs1456896	7	50304461	T	C	0.697556	0.139301	0.0251	2.89701E-08	0.6784	0.0028	0.0068	0.6808	30.8007311

rs148844907	6	31628397	A	T	0.00828273	0.958001	0.1419	1.47096E-11	0.0137	0.0694	0.0313	0.02663	45.57924572
rs151314883	22	39735087	A	G	0.157585	-0.223994	0.0327	7.12361E-12	0.1565	-0.0096	0.0089	0.2807	46.92208104
rs1887428	9	4984530	C	G	0.623303	-0.168099	0.0243	4.22377E-12	0.6179	0.0089	0.0066	0.1773	47.8539413
rs1932990	13	44460242	T	C	0.253841	0.152901	0.0263	6.02102E-09	0.2207	0.0021	0.0076	0.7835	33.79941274
rs2076756	16	50756881	G	A	0.283672	0.399806	0.0242	3.24489E-61	0.2448	-0.011	0.0074	0.1389	272.9404372
rs2188962	5	131770805	T	C	0.439546	0.212398	0.0228	1.35988E-20	0.4102	-0.0075	0.0065	0.2479	86.78229918
rs2505640	10	35459497	G	A	0.643588	-0.145701	0.0237	7.61202E-10	0.6515	-0.0124	0.0067	0.06424	37.79447988
rs281379	19	49214274	A	G	0.489275	0.139797	0.0238	4.26403E-09	0.5058	-0.0068	0.0065	0.2949	34.50180285
rs28701841	6	106530330	A	G	0.116563	0.224303	0.0373	1.85102E-09	0.1063	0.0105	0.0107	0.3255	36.16200491
rs3091315	17	32593665	G	A	0.265588	-0.179501	0.0263	9.52138E-12	0.2843	0.0054	0.0071	0.4482	46.58244156
rs3810936	9	117552885	C	T	0.698408	0.207799	0.0263	2.46207E-15	0.6924	0.0026	0.007	0.7109	62.42742327
rs444210	6	167390242	G	A	0.546838	0.163402	0.0229	1.02494E-12	0.5472	0.0155	0.0064	0.01523	50.91476822
rs4851586	2	103064264	C	T	0.759729	-0.168899	0.0261	9.93574E-11	0.7723	-0.0122	0.0076	0.1104	41.87676664

rs4902642	14	69210199	A	G	0.409159	-0.129198	0.0236	4.339E-08	0.4298	0.0027	0.0065	0.6775	29.97005746
rs4921497	5	158848253	G	C	0.329415	0.160298	0.0244	5.49414E-11	0.3287	-0.0084	0.0068	0.2171	43.15951492
rs6588243	1	67603383	C	A	0.589982	0.131704	0.0234	1.77701E-08	0.5674	0.0071	0.0064	0.2663	31.67861717
rs6704109	1	172857050	T	C	0.256123	0.202002	0.0256	2.77013E-15	0.2408	-0.0054	0.0074	0.4676	62.26319581
rs697693	1	7886424	A	G	0.201415	0.172296	0.0281	8.35507E-10	0.1904	0.004	0.0082	0.6243	37.5956632
rs7276302	21	45614159	G	A	0.608306	-0.171598	0.0231	1.22603E-13	0.6134	-0.0051	0.007	0.4672	55.18238714
rs72798422	16	50866917	C	T	0.047701	0.590392	0.0508	3.19301E-31	0.0368	0.0064	0.0172	0.7094	135.0683217
rs7713270	5	40440063	T	C	0.623824	0.296602	0.0241	6.96627E-35	0.6065	-0.0036	0.0065	0.5792	151.4656194
rs80262450	18	12818922	A	G	0.112701	0.283102	0.0353	1.08193E-15	0.1232	0.0138	0.0098	0.1609	64.31858245
rs8178977	19	1106477	C	G	0.238841	0.1928	0.0274	2.05589E-12	0.2467	0.0052	0.0077	0.5021	49.51228089
rs907092	17	37922259	A	G	0.471412	0.130396	0.0228	1.011E-08	0.4747	-0.0091	0.0064	0.1542	32.70836568
rs921720	8	126534671	G	A	0.618841	0.162895	0.0237	6.39588E-12	0.6297	-0.0007	0.0066	0.9155	47.24097104

Abbreviation: CD= Crohn’s disease, T2DM= type 2 diabetes mellitus, SNPs= single-nucleotide polymorphisms, Chr= Chromosome, EA= Effect Allele, OA= Other Allele, EAF= effect allele frequency, SE= standard error.

Table S10. Characteristics of the SNPs related to T1DM and IBD

SNP	Chr	Position	EA	OA	Exposure: T1DM				Outcome: IBD				F statistic
					EAF	β	SE	*P*	EAF	β	SE	*P*	
rs10224046	7	20386338	G	T	0.324	0.085811	0.324	2.71E-08	0.314974	0.0300984	0.0179	0.0924592	30.90412034
rs10275896	7	26860134	C	T	0.232	-0.121178	0.232	2.60016E-13	0.246602	0.0377019	0.0193	0.0505406	53.48782826
rs1050979	6	410417	G	A	0.515	0.106196	0.515	5.64937E-14	0.506769	0.000100005	0.017	0.9947	56.49284772
rs10751776	1	25296743	C	A	0.51	0.078145	0.51	2.66999E-08	0.500769	-0.0339955	0.0168	0.0429705	30.93497309
rs10801128	1	192515849	G	A	0.717	0.096073	0.717	8.98008E-10	0.710256	0.00769957	0.0186	0.6783	37.53662269
rs10844597	12	9874899	A	G	0.506	-0.089844	0.506	1.2E-10	0.499654	-0.0393959	0.0168	0.0191201	41.46130523
rs11203203	21	43836186	A	G	0.349	0.143803	0.349	1.81009E-23	0.381231	0.0267977	0.0172	0.1199	99.65735753
rs112733823	6	30782661	T	C	0.142	0.382619	0.142	7.41993E-81	0.074256	-0.0392976	0.0477	0.4098	362.6492016
rs113374757	19	47256542	T	C	0.163	-0.171277	0.163	1.63005E-16	0.157885	-0.0388968	0.0236	0.099259	68.0090835
rs114378220	5	110566360	T	C	0.0703	0.177902	0.0703	5.10999E-09	0.0707052	-0.0331019	0.0388	0.3942	34.1474466

rs12128789	1	212852832	C	T	0.132	0.126969	0.132	3.73001E-09	0.134231	0.0740987	0.0246	0.00261999	34.7620713
rs12257077	10	6071545	T	C	0.0319	0.231245	0.0319	3.91003E-10	0.0411744	0.038201	0.043	0.3749	39.15599723
rs12464462	2	60636416	G	A	0.41	-0.087955	0.41	8.60994E-10	0.397256	-0.00960374	0.0171	0.574	37.61509771
rs12644686	4	185302902	G	C	0.194	-0.10775	0.194	2.44E-08	0.185	-0.0270995	0.0246	0.2703	31.10429151
rs12742756	1	38347417	G	A	0.428	-0.083107	0.428	3.53997E-08	0.429859	0.0222047	0.0176	0.2079	30.38402755
rs12927355	16	11194771	T	C	0.316	-0.203881	0.316	4.40961E-41	0.330769	-0.0248051	0.0178	0.1634	180.198982
rs13259300	8	120082941	C	A	0.598	-0.092191	0.598	3.27997E-10	0.632372	0.00729656	0.0178	0.6798	39.49808449
rs1350275	14	98489306	G	T	0.698	-0.093665	0.698	8.85992E-10	0.716398	-0.0219963	0.0185	0.2359	37.56102617
rs1574285	9	4283137	T	G	0.591	-0.126548	0.591	4.26973E-19	0.584115	0.0171029	0.0171	0.3171	79.74612252
rs1611236	6	29752020	A	G	0.299	-0.260162	0.299	1.83021E-50	0.318513	-0.0305971	0.0221	0.1651	223.1725458
rs17106304	14	69260511	G	C	0.656	0.115351	0.656	6.82967E-15	0.669769	-0.0361969	0.0179	0.0427199	60.64783764
rs17323934	7	26904330	G	C	0.223	-0.129663	0.223	1.26009E-14	0.241602	0.0408949	0.0196	0.0373801	59.44063426
rs17623914	1	198670555	C	T	0.0997	-0.134887	0.0997	7.97003E-09	0.112115	0.0411971	0.0268	0.1243	33.28235339

rs1808094	18	67526026	C	T	0.524	-0.113651	0.524	2.39994E-14	0.518911	-0.0509022	0.0171	0.00287303	58.18003604
rs1881146	2	12634794	T	A	0.311	-0.09517	0.311	4.57004E-08	0.323885	-0.0118989	0.0188	0.524901	29.8918146
rs1947178	8	59872177	G	A	0.792	-0.103267	0.792	1.67001E-09	0.761769	-0.0189984	0.0197	0.3341	36.32499774
rs202535	20	1666989	A	C	0.828	-0.141437	0.828	1.79019E-14	0.827628	-0.00700447	0.0222	0.7524	58.74786775
rs229527	22	37581485	A	C	0.416	0.104077	0.416	1.82012E-13	0.419372	-0.00130085	0.0171	0.9404	54.19179993
rs231972	16	28538336	C	A	0.118	0.170939	0.118	5.12035E-16	0.126885	-0.0330962	0.0263	0.2073	65.75062743
rs238265	13	42930659	G	T	0.695	-0.090825	0.695	2.07998E-09	0.706282	-0.0511968	0.0183	0.00508405	35.90269152
rs2493411	1	120506786	C	T	0.132	0.127063	0.132	1.28E-08	0.123141	-0.0659001	0.0262	0.0118201	32.36430582
rs2543537	22	37531117	T	C	0.46	-0.083441	0.46	5.58998E-09	0.423859	0.0246048	0.0175	0.1593	33.97157022
rs2611211	4	166575439	T	C	0.824	-0.143854	0.824	1.38995E-14	0.833885	-0.00399798	0.0225	0.8601	59.24773561
rs28648882	2	242278547	A	G	0.224	0.098731	0.224	3.02998E-08	0.237769	-0.027402	0.0197	0.1645	30.68636577
rs3087243	2	204738919	A	G	0.422	-0.19913	0.422	1.16011E-44	0.447513	-0.0129029	0.0168	0.441199	196.5958613
rs34593439	15	79234957	A	G	0.108	-0.218071	0.108	1.53993E-19	0.0846654	0.0182033	0.0334	0.5862	81.74801862

rs35327136	17	43512206	A	C	0.156	-0.119196	0.156	3.37E-10	0.182398	-0.0339955	0.0217	0.1167	39.44777191
rs3802214	8	141616183	C	T	0.799	-0.106609	0.799	2.95999E-08	0.815	0.00540458	0.0217	0.8034	30.73471362
rs4490209	2	100766711	G	C	0.36	-0.084522	0.36	4.54999E-08	0.367398	-0.0518047	0.0174	0.003004	29.90123431
rs4548024	6	138165744	C	T	0.234	-0.095737	0.234	9.94993E-09	0.210859	-0.00259663	0.021	0.9021	32.85266964
rs55993634	16	75236763	G	C	0.0849	0.219371	0.0849	2.28981E-19	0.086487	0.0638028	0.031	0.0395103	80.97046908
rs56994090	14	101306447	C	T	0.431	-0.134255	0.431	3.59998E-20	0.413744	-0.0010994	0.0181	0.9508	84.62764469
rs57209021	17	66164918	T	C	0.226	0.100693	0.226	3.73001E-08	0.235372	0.0082955	0.0201	0.679201	30.28578147
rs574384	1	36087661	A	C	0.895	-0.133602	0.895	2.19999E-08	0.895885	0.0190965	0.0283	0.4991	31.31142107
rs607703	11	128604174	T	C	0.484	0.092015	0.484	1.17001E-10	0.462602	0.0239021	0.0168	0.1546	41.52033473
rs61759532	17	7240391	T	C	0.235	0.118379	0.235	1.90999E-10	0.217885	0.0009995	0.0231	0.9639	40.56306244
rs6434435	2	191953864	A	G	0.16	-0.122856	0.16	1.22999E-10	0.179	-0.00399798	0.022	0.8553	41.42155385
rs7068821	10	90051035	T	G	0.251	-0.165103	0.251	5.06991E-24	0.247628	-0.00569619	0.0193	0.768	102.1828485
rs7130222	11	2090456	G	T	0.308	-0.091985	0.308	1.29999E-08	0.326026	-0.0250048	0.0185	0.1758	32.32441511

rs722988	10	33426147	C	T	0.353	0.082649	0.353	9.78003E-09	0.377231	0.015205	0.0172	0.3765	32.88718487
rs7668577	4	26123319	C	A	0.312	0.093652	0.312	7.26006E-10	0.307346	0.0532004	0.0188	0.00478597	37.94683177
rs7776597	7	51015582	G	A	0.959	0.244413	0.959	1.82012E-11	0.959481	0.0465048	0.0426	0.2745	45.15587255
rs7795896	7	117086613	T	C	0.692	-0.135435	0.692	1.58016E-16	0.701628	-0.00589736	0.0183	0.749	68.06551538
rs78325861	10	72378489	G	C	0.039	-0.282082	0.039	2.30994E-11	0.045	0.0197028	0.045	0.6611	44.6918946
rs8046043	16	80284024	C	G	0.392	-0.084587	0.392	2.49E-08	0.410398	-0.017604	0.0173	0.3076	31.07061469
rs855330	1	64113889	C	T	0.259	0.111208	0.259	4.8899E-11	0.234372	0.0031951	0.02	0.8711	43.21919841

Abbreviation: T1DM= type 1 diabetes mellitus, IBD= inflammatory bowel disease, SNPs= single-nucleotide polymorphisms, Chr= Chromosome, EA= Effect Allele, OA= Other Allele, EAF= effect allele frequency, SE= standard error.

Table S11. Characteristics of the SNPs related to T1DM and UC

SNP	Chr	Position	EA	OA	Exposure: T1DM				Outcome: UC				F statistic
					EAF	β	SE	*P*	EAF	β	SE	*P*	
rs10224046	7	20386338	G	T	0.324	0.085811	0.015436	2.71E-08	0.313286	0.028204	0.0226	0.2108	30.90412034
rs10275896	7	26860134	C	T	0.232	-0.121178	0.016569	2.60016E-13	0.243238	0.0239034	0.0244	0.3275	53.48782826
rs1050979	6	410417	G	A	0.515	0.106196	0.014129	5.64937E-14	0.505682	-0.00990083	0.0213	0.640801	56.49284772
rs10751776	1	25296743	C	A	0.51	0.078145	0.01405	2.66999E-08	0.50173	-0.0134985	0.0211	0.5231	30.93497309
rs10801128	1	192515849	G	A	0.717	0.096073	0.015681	8.98008E-10	0.713508	0.0198048	0.0236	0.4026	37.53662269
rs10844597	12	9874899	A	G	0.506	-0.089844	0.013953	1.2E-10	0.50273	0.0010994	0.0213	0.959	41.46130523
rs11203203	21	43836186	A	G	0.349	0.143803	0.014405	1.81009E-23	0.379508	0.0218006	0.0217	0.3155	99.65735753
rs112733823	6	30782661	T	C	0.142	0.382619	0.020092	7.41993E-81	0.073746	0.025205	0.0605	0.6775	362.6492016
rs113374757	19	47256542	T	C	0.163	-0.171277	0.020769	1.63005E-16	0.157222	-0.065798	0.0301	0.0288702	68.0090835
rs114378220	5	110566360	T	C	0.0703	0.177902	0.030444	5.10999E-09	0.0711888	-0.0418016	0.0486	0.39	34.1474466

rs12128789	1	212852832	C	T	0.132	0.126969	0.021535	3.73001E-09	0.13327	0.0719042	0.0309	0.0199301	34.7620713
rs12257077	10	6071545	T	C	0.0319	0.231245	0.036955	3.91003E-10	0.0407888	0	0.0552	0.9994	39.15599723
rs12464462	2	60636416	G	A	0.41	-0.087955	0.014341	8.60994E-10	0.398508	-0.00649884	0.0215	0.7625	37.61509771
rs12644686	4	185302902	G	C	0.194	-0.10775	0.01932	2.44E-08	0.183492	-0.0289955	0.0312	0.352	31.10429151
rs12742756	1	38347417	G	A	0.428	-0.083107	0.015077	3.53997E-08	0.428762	0.0213973	0.0222	0.335	30.38402755
rs12927355	16	11194771	T	C	0.316	-0.203881	0.015188	4.40961E-41	0.328952	-0.0209989	0.0226	0.3522	180.198982
rs13259300	8	120082941	C	A	0.598	-0.092191	0.014669	3.27997E-10	0.629984	0.0107981	0.0223	0.629001	39.49808449
rs1350275	14	98489306	G	T	0.698	-0.093665	0.015283	8.85992E-10	0.716952	-0.0349037	0.0233	0.1343	37.56102617
rs1574285	9	4283137	T	G	0.591	-0.126548	0.014171	4.26973E-19	0.583762	0.00679685	0.0216	0.7533	79.74612252
rs1611236	6	29752020	A	G	0.299	-0.260162	0.017415	1.83021E-50	0.317714	-0.0270021	0.0279	0.3329	223.1725458
rs17106304	14	69260511	G	C	0.656	0.115351	0.014812	6.82967E-15	0.671238	-0.000499875	0.0226	0.9825	60.64783764
rs17323934	7	26904330	G	C	0.223	-0.129663	0.016818	1.26009E-14	0.237984	0.0235962	0.0248	0.3414	59.44063426
rs17623914	1	198670555	C	T	0.0997	-0.134887	0.023381	7.97003E-09	0.109984	0.00690378	0.0341	0.8401	33.28235339

rs1808094	18	67526026	C	T	0.524	-0.113651	0.0149	2.39994E-14	0.515952	-0.0419957	0.0214	0.0493503	58.18003604
rs1881146	2	12634794	T	A	0.311	-0.09517	0.017407	4.57004E-08	0.325222	-0.0423025	0.0235	0.0721705	29.8918146
rs1947178	8	59872177	G	A	0.792	-0.103267	0.017134	1.67001E-09	0.765	-0.0077995	0.025	0.7548	36.32499774
rs202535	20	1666989	A	C	0.828	-0.141437	0.018453	1.79019E-14	0.828762	0.0227981	0.0281	0.4186	58.74786775
rs229527	22	37581485	A	C	0.416	0.104077	0.014138	1.82012E-13	0.416714	-0.0114958	0.0216	0.5925	54.19179993
rs231972	16	28538336	C	A	0.118	0.170939	0.021081	5.12035E-16	0.128746	-0.0057037	0.0327	0.8608	65.75062743
rs238265	13	42930659	G	T	0.695	-0.090825	0.015158	2.07998E-09	0.70673	-0.00960374	0.0231	0.6785	35.90269152
rs2493411	1	120506786	C	T	0.132	0.127063	0.022335	1.28E-08	0.124746	-0.0329995	0.0328	0.3137	32.36430582
rs2543537	22	37531117	T	C	0.46	-0.083441	0.014316	5.58998E-09	0.42454	0.0394995	0.0221	0.0731004	33.97157022
rs2611211	4	166575439	T	C	0.824	-0.143854	0.018689	1.38995E-14	0.833476	0.00129916	0.0283	0.9636	59.24773561
rs28648882	2	242278547	A	G	0.224	0.098731	0.017823	3.02998E-08	0.237222	-0.00130085	0.0249	0.9596	30.68636577
rs3087243	2	204738919	A	G	0.422	-0.19913	0.014202	1.16011E-44	0.444714	-0.005103	0.0212	0.8089	196.5958613
rs34593439	15	79234957	A	G	0.108	-0.218071	0.024119	1.53993E-19	0.0861444	0.0195967	0.0419	0.6406	81.74801862

rs35327136	17	43512206	A	C	0.156	-0.119196	0.018978	3.37E-10	0.181714	-0.0150955	0.0275	0.5829	39.44777191
rs3802214	8	141616183	C	T	0.799	-0.106609	0.01923	2.95999E-08	0.812476	-0.0117013	0.0271	0.666099	30.73471362
rs4548024	6	138165744	C	T	0.234	-0.095737	0.016703	9.94993E-09	0.208508	-0.0392015	0.0266	0.1397	32.85266964
rs4820827	22	30477589	C	T	0.621	-0.129657	0.014267	1.00995E-19	0.620572	0.0513038	0.0219	0.0190902	82.58983812
rs55993634	16	75236763	G	C	0.0849	0.219371	0.024379	2.28981E-19	0.08527	0.0846977	0.0387	0.0286299	80.97046908
rs56994090	14	101306447	C	T	0.431	-0.134255	0.014594	3.59998E-20	0.415286	0.0242005	0.0225	0.2821	84.62764469
rs57209021	17	66164918	T	C	0.226	0.100693	0.018297	3.73001E-08	0.235	0.018704	0.0255	0.4634	30.28578147
rs574384	1	36087661	A	C	0.895	-0.133602	0.023876	2.19999E-08	0.895984	-0.0180011	0.0356	0.6142	31.31142107
rs607703	11	128604174	T	C	0.484	0.092015	0.01428	1.17001E-10	0.464032	0.0171029	0.0211	0.4193	41.52033473
rs61759532	17	7240391	T	C	0.235	0.118379	0.018587	1.90999E-10	0.219238	-0.0142003	0.0289	0.6221	40.56306244
rs6434435	2	191953864	A	G	0.16	-0.122856	0.019089	1.22999E-10	0.179238	-0.00190181	0.0277	0.9457	41.42155385
rs663743	11	64107735	A	G	0.349	-0.099964	0.015092	3.50026E-11	0.333714	-0.0505041	0.0226	0.0256898	43.8726289
rs6908626	6	91005743	T	G	0.166	0.202923	0.01852	6.14045E-28	0.193984	-0.0476998	0.0294	0.1042	120.0550917

rs7068821	10	90051035	T	G	0.251	-0.165103	0.016333	5.06991E-24	0.247762	0.00560427	0.0242	0.8165	102.1828485
rs7130222	11	2090456	G	T	0.308	-0.091985	0.016179	1.29999E-08	0.32646	-0.0213015	0.0233	0.3595	32.32441511
rs722988	10	33426147	C	T	0.353	0.082649	0.014412	9.78003E-09	0.376508	0.00930314	0.0217	0.6688	32.88718487
rs7301381	12	9123932	C	T	0.464	-0.093588	0.014258	5.25049E-11	0.45181	0.0519988	0.0212	0.0140301	43.08470384
rs7668577	4	26123319	C	A	0.312	0.093652	0.015203	7.26006E-10	0.304524	0.0419998	0.0234	0.0726608	37.94683177
rs7776597	7	51015582	G	A	0.959	0.244413	0.036372	1.82012E-11	0.958598	0.0226035	0.0529	0.6694	45.15587255
rs7795896	7	117086613	T	C	0.692	-0.135435	0.016416	1.58016E-16	0.701746	0.00890027	0.0231	0.6983	68.06551538
rs78325861	10	72378489	G	C	0.039	-0.282082	0.042195	2.30994E-11	0.044508	0.0360003	0.056	0.5203	44.6918946
rs8046043	16	80284024	C	G	0.392	-0.084587	0.015175	2.49E-08	0.41146	-0.0163022	0.0218	0.4558	31.07061469
rs855330	1	64113889	C	T	0.259	0.111208	0.016916	4.8899E-11	0.233492	-0.0145043	0.0253	0.566301	43.21919841
rs9517712	13	100079833	C	T	0.741	-0.102063	0.015805	1.06001E-10	0.750698	-0.0575045	0.0244	0.0183202	41.70111421

Abbreviation: T1DM= type 1 diabetes mellitus, UC= ulcerative colitis, SNPs= single-nucleotide polymorphisms, Chr= Chromosome, EA= Effect Allele, OA= Other Allele, EAF= effect allele frequency, SE= standard error.

Table S12. Characteristics of the SNPs related to T1DM and CD

SNP	Chr	Position	EA	OA	Exposure: T1DM				Outcome: CD				F statistic
					EAF	β	SE	*P*	EAF	β	SE	*P*	
rs10224046	7	20386338	G	T	0.324	0.085811	0.015436	2.71E-08	0.313282	0.0383979	0.0243	0.1136	30.90412034
rs1050979	6	410417	G	A	0.515	0.106196	0.014129	5.64937E-14	0.510711	0.0316971	0.0231	0.1709	56.49284772
rs10751776	1	25296743	C	A	0.51	0.078145	0.01405	2.66999E-08	0.503578	-0.0603987	0.0229	0.00832703	30.93497309
rs10801128	1	192515849	G	A	0.717	0.096073	0.015681	8.98008E-10	0.70443	0.00290421	0.0253	0.9088	37.53662269
rs11203203	21	43836186	A	G	0.349	0.143803	0.014405	1.81009E-23	0.383996	0.0301023	0.0233	0.1973	99.65735753
rs112733823	6	30782661	T	C	0.142	0.382619	0.020092	7.41993E-81	0.075289	-0.0709989	0.0645	0.2713	362.6492016
rs113374757	19	47256542	T	C	0.163	-0.171277	0.020769	1.63005E-16	0.157856	-0.000500125	0.0318	0.9864	68.0090835
rs114378220	5	110566360	T	C	0.0703	0.177902	0.030444	5.10999E-09	0.0709715	-0.000700245	0.053	0.9892	34.1474466
rs12128789	1	212852832	C	T	0.132	0.126969	0.021535	3.73001E-09	0.132282	0.087095	0.0338	0.00991791	34.7620713
rs12257077	10	6071545	T	C	0.0319	0.231245	0.036955	3.91003E-10	0.0422986	0.0911013	0.0569	0.1091	39.15599723

rs12464462	2	60636416	G	A	0.41	-0.087955	0.014341	8.60994E-10	0.393144	0.00859685	0.0234	0.7134	37.61509771
rs12644686	4	185302902	G	C	0.194	-0.10775	0.01932	2.44E-08	0.19	-0.0243023	0.033	0.4622	31.10429151
rs12742756	1	38347417	G	A	0.428	-0.083107	0.015077	3.53997E-08	0.430996	0.0182964	0.0241	0.4475	30.38402755
rs12927355	16	11194771	T	C	0.316	-0.203881	0.015188	4.40961E-41	0.338144	-0.0130954	0.0241	0.5852	180.198982
rs13259300	8	120082941	C	A	0.598	-0.092191	0.014669	3.27997E-10	0.638141	0.00549507	0.0245	0.8227	39.49808449
rs1350275	14	98489306	G	T	0.698	-0.093665	0.015283	8.85992E-10	0.720144	-0.00750179	0.0253	0.766399	37.56102617
rs1574285	9	4283137	T	G	0.591	-0.126548	0.014171	4.26973E-19	0.582141	0.0356953	0.0233	0.1254	79.74612252
rs1611236	6	29752020	A	G	0.299	-0.260162	0.017415	1.83021E-50	0.32243	-0.0491044	0.03	0.1024	223.1725458
rs17623914	1	198670555	C	T	0.0997	-0.134887	0.023381	7.97003E-09	0.113852	0.0934978	0.0358	0.00902298	33.28235339
rs1808094	18	67526026	C	T	0.524	-0.113651	0.0149	2.39994E-14	0.526148	-0.0518996	0.0235	0.02688	58.18003604
rs1881146	2	12634794	T	A	0.311	-0.09517	0.017407	4.57004E-08	0.322856	0.0331949	0.0257	0.197	29.8918146
rs1947178	8	59872177	G	A	0.792	-0.103267	0.017134	1.67001E-09	0.756578	-0.0259991	0.0263	0.3239	36.32499774
rs202535	20	1666989	A	C	0.828	-0.141437	0.018453	1.79019E-14	0.824859	-0.0172988	0.0297	0.559899	58.74786775

rs229527	22	37581485	A	C	0.416	0.104077	0.014138	1.82012E-13	0.428141	0.0126003	0.023	0.5829	54.19179993
rs231972	16	28538336	C	A	0.118	0.170939	0.021081	5.12035E-16	0.125859	-0.0446961	0.0363	0.2177	65.75062743
rs2543537	22	37531117	T	C	0.46	-0.083441	0.014316	5.58998E-09	0.422	0.000099995	0.0237	0.9959	33.97157022
rs2611211	4	166575439	T	C	0.824	-0.143854	0.018689	1.38995E-14	0.835	0.0082955	0.0308	0.787501	59.24773561
rs28648882	2	242278547	A	G	0.224	0.098731	0.017823	3.02998E-08	0.241718	-0.0465048	0.0269	0.0840601	30.68636577
rs3087243	2	204738919	A	G	0.422	-0.19913	0.014202	1.16011E-44	0.456289	-0.0325025	0.0228	0.1535	196.5958613
rs34593439	15	79234957	A	G	0.108	-0.218071	0.024119	1.53993E-19	0.0838718	0.018704	0.0459	0.684101	81.74801862
rs35327136	17	43512206	A	C	0.156	-0.119196	0.018978	3.37E-10	0.187718	-0.0533038	0.0292	0.0680205	39.44777191
rs3802214	8	141616183	C	T	0.799	-0.106609	0.01923	2.95999E-08	0.818426	0.0236986	0.0297	0.425	30.73471362
rs4490209	2	100766711	G	C	0.36	-0.084522	0.015457	4.54999E-08	0.377711	-0.0028958	0.0234	0.9003	29.90123431
rs4548024	6	138165744	C	T	0.234	-0.095737	0.016703	9.94993E-09	0.209852	0.0449032	0.0285	0.1148	32.85266964
rs55993634	16	75236763	G	C	0.0849	0.219371	0.024379	2.28981E-19	0.087285	0.032203	0.0426	0.4494	80.97046908
rs56994090	14	101306447	C	T	0.431	-0.134255	0.014594	3.59998E-20	0.410859	-0.0251023	0.0251	0.3162	84.62764469

rs57209021	17	66164918	T	C	0.226	0.100693	0.018297	3.73001E-08	0.238715	-0.00589736	0.0272	0.8275	30.28578147
rs574384	1	36087661	A	C	0.895	-0.133602	0.023876	2.19999E-08	0.896	0.0635005	0.0386	0.0997011	31.31142107
rs607703	11	128604174	T	C	0.484	0.092015	0.01428	1.17001E-10	0.457282	0.0298986	0.0229	0.1927	41.52033473
rs61759532	17	7240391	T	C	0.235	0.118379	0.018587	1.90999E-10	0.21443	0.0184979	0.0318	0.560899	40.56306244
rs6434435	2	191953864	A	G	0.16	-0.122856	0.019089	1.22999E-10	0.18043	-0.0260976	0.0299	0.3835	41.42155385
rs7068821	10	90051035	T	G	0.251	-0.165103	0.016333	5.06991E-24	0.243715	-0.0037972	0.0265	0.8859	102.1828485
rs7130222	11	2090456	G	T	0.308	-0.091985	0.016179	1.29999E-08	0.327574	-0.0333961	0.025	0.1822	32.32441511
rs722988	10	33426147	C	T	0.353	0.082649	0.014412	9.78003E-09	0.376708	0.0453949	0.0233	0.0510305	32.88718487
rs7301381	12	9123932	C	T	0.464	-0.093588	0.014258	5.25049E-11	0.450563	0.0636003	0.0229	0.00549402	43.08470384
rs7511678	1	200832857	A	G	0.229	0.093508	0.01689	3.09001E-08	0.205852	0.0539009	0.0282	0.0555904	30.65054754
rs7776597	7	51015582	G	A	0.959	0.244413	0.036372	1.82012E-11	0.960798	0.100705	0.06	0.0930208	45.15587255
rs78325861	10	72378489	G	C	0.039	-0.282082	0.042195	2.30994E-11	0.044715	-0.0199987	0.0622	0.7475	44.6918946
rs8046043	16	80284024	C	G	0.392	-0.084587	0.015175	2.49E-08	0.411574	-0.013298	0.0235	0.5714	31.07061469

rs855330	1	64113889	C	T	0.259	0.111208	0.016916	4.8899E-11	0.235856	0.0289037	0.027	0.2844	43.21919841
rs9517712	13	100079833	C	T	0.741	-0.102063	0.015805	1.06001E-10	0.750437	-0.0795965	0.0264	0.00253099	41.70111421

Abbreviation: T1DM= type 1 diabetes mellitus, CD= Crohn’s disease, SNPs= single-nucleotide polymorphisms, Chr= Chromosome, EA= Effect Allele, OA= Other Allele, EAF= effect allele frequency, SE= standard error.

Table S13. Characteristics of the SNPs related to T2DM and IBD

SNP	Chr	Position	EA	OA	Exposure: T2DM				Outcome: IBD				F statistic
					EAF	β	SE	*P*	EAF	β	SE	*P*	
rs10033601	4	153252061	A	G	0.7138	0.0451	0.0071	2.381E-10	0.706141	0.00580313	0.0189	0.7573	40.34933545
rs1025216	3	168212861	A	G	0.1278	-0.0543	0.0096	1.729E-08	0.127	-0.00619918	0.0253	0.805	31.99316406
rs10260837	7	28181129	T	C	0.1111	0.0577	0.0102	1.337E-08	0.116115	0.045499	0.0262	0.0821297	32.00009612
rs1043246	17	3828086	C	G	0.8445	-0.0566	0.0096	4.206E-09	0.872513	-0.0329985	0.0326	0.311	34.76085069
rs10502791	18	40071369	A	T	0.7888	-0.0426	0.0077	3.837E-08	0.789256	-0.0212951	0.0204	0.2974	30.608197
rs10509406	10	80959517	T	C	0.8277	0.0564	0.0086	5.061E-11	0.822141	-0.0145047	0.0222	0.5126	43.00919416
rs10771260	12	26253557	A	C	0.6113	-0.0367	0.0066	2.64E-08	0.612372	-0.00559563	0.0173	0.743599	30.92033976
rs10821310	9	96928812	T	C	0.3193	0.0372	0.0068	4.602E-08	0.317115	0.0277027	0.0184	0.1318	29.92733564
rs10848958	12	4031104	T	C	0.1966	-0.0451	0.0083	4.968E-08	0.189744	0.0223973	0.0214	0.2968	29.5254754
rs10853047	17	62199136	T	C	0.8627	-0.0519	0.0094	3.636E-08	0.854372	0.0108015	0.024	0.653899	30.48449525

rs10965199	9	21954653	T	C	0.0361	-0.1139	0.019	1.853E-09	0.0496602	0.0446004	0.0417	0.2851	35.93686981
rs11049086	12	27818770	A	G	0.1948	-0.046	0.0082	1.775E-08	0.191372	0.00499749	0.0213	0.816	31.46936347
rs1105291	15	91502383	T	C	0.1429	0.0613	0.0091	1.702E-11	0.137487	0.0275957	0.0249	0.2683	45.37724912
rs11063029	12	4301301	T	C	0.0581	0.0858	0.0138	5.364E-10	0.0581859	0.0465973	0.0368	0.2055	38.65595463
rs11108086	12	95914758	T	C	0.9326	-0.0697	0.0128	4.855E-08	0.934744	0.0194987	0.0341	0.5665	29.65142822
rs11122800	2	121305604	A	G	0.8534	0.0571	0.0091	3.647E-10	0.855372	0.0166014	0.0241	0.4923	39.37217727
rs111620997	12	66162603	A	G	0.9513	-0.0857	0.0157	4.85E-08	0.958	0.00639948	0.0522	0.9021	29.79630005
rs111686785	5	14738965	A	G	0.9694	-0.1169	0.0194	1.588E-09	0.975628	-0.0366017	0.0694	0.597901	36.30994261
rs11235566	11	72403388	T	C	0.5456	0.0353	0.0064	3.258E-08	0.544256	-0.00300451	0.0168	0.8605	30.42211914
rs112538930	7	14859137	T	C	0.1843	0.0605	0.0083	2.588E-13	0.171859	0.0274011	0.0229	0.2315	53.13180433
rs11514705	7	15016108	T	C	0.3309	0.0377	0.0069	4.881E-08	0.330628	-0.00979784	0.0201	0.6255	29.85276202
rs11564714	11	2190418	C	G	0.0855	0.0715	0.0121	3.934E-09	0.0866282	-0.0239034	0.0375	0.5247	34.91735537
rs11655029	17	17649172	T	C	0.3207	0.0463	0.0069	2.083E-11	0.328115	0.00850374	0.0179	0.634	45.02604495

rs11680058	2	16574669	A	G	0.8648	0.0581	0.0104	2.081E-08	0.863256	-0.0104039	0.0304	0.732	31.20941198
rs116859590	10	114752410	T	C	0.0263	0.229	0.021	1.415E-27	0.0311565	-0.0716034	0.0587	0.2225	118.9138322
rs116964396	8	41505849	A	C	0.0339	0.107	0.0188	1.367E-08	0.0308271	-0.157403	0.0517	0.00230802	32.39305115
rs117316450	11	14518419	C	G	0.9803	-0.1274	0.0232	4.229E-08	0.978372	0.0443996	0.0599	0.4584	30.15524673
rs117465240	11	69444235	A	G	0.0319	-0.1053	0.0193	4.608E-08	0.0400372	0.0680044	0.0523	0.193	29.76748369
rs117753849	10	114603431	A	G	0.9832	-0.1924	0.0252	2.443E-14	0.982372	0.049999	0.0664	0.4511	58.2920131
rs117987174	10	114756459	A	G	0.0135	-0.1723	0.0305	1.558E-08	0.00685	-0.0460021	0.1257	0.7147	31.91323838
rs11964747	6	20485898	T	C	0.1861	-0.0577	0.0083	3.042E-12	0.184602	0.0489997	0.0225	0.0292698	48.32762375
rs12154627	7	130422934	T	C	0.5045	0.0469	0.0065	5.019E-13	0.522885	-0.0292023	0.0172	0.0890697	52.06177515
rs12222793	11	92667047	A	G	0.5465	-0.0434	0.0066	4.72E-11	0.536256	-0.00920221	0.0186	0.6226	43.2405877
rs12419690	11	45858584	A	G	0.5524	-0.0351	0.0064	3.892E-08	0.568346	0.0463014	0.017	0.00664293	30.07836914
rs12523853	6	20429804	T	C	0.0401	0.0973	0.0169	7.831E-09	0.0388576	0.0239997	0.0482	0.6193	33.14761388
rs12625671	20	42994812	T	C	0.8849	-0.0757	0.0101	5.025E-14	0.881256	-0.00540458	0.0268	0.8408	56.17576708

rs12640250	4	17792869	A	C	0.2834	-0.04	0.0071	1.93E-08	0.276398	-0.028204	0.019	0.1376	31.73973418
rs12680202	8	12617942	A	T	0.3206	0.0411	0.0071	7.803E-09	0.309372	-0.0127003	0.0195	0.5149	33.50942273
rs12703101	7	150504840	T	C	0.714	-0.04	0.007	1.184E-08	0.700282	-0.0239034	0.0186	0.1971	32.65306122
rs12974635	19	4947056	T	C	0.2016	0.0452	0.0081	2.065E-08	0.199744	-0.0031951	0.0216	0.8803	31.13915562
rs13099586	3	23294322	T	C	0.0904	-0.0634	0.0113	2.062E-08	0.0825282	-0.017604	0.0307	0.565501	31.47905083
rs13293121	9	81881830	A	G	0.9316	0.0864	0.0134	1.142E-10	0.942513	-0.0421041	0.0371	0.2571	41.57362442
rs138960826	3	185530177	A	G	0.0479	0.0877	0.0154	1.212E-08	0.0460859	-0.00749804	0.0443	0.8655	32.43080621
rs1436955	15	62404382	T	C	0.2718	-0.0394	0.0072	4.933E-08	0.263744	-0.00520351	0.019	0.783201	29.94521605
rs144102357	11	127999754	A	G	0.1361	-0.0529	0.0094	1.979E-08	0.149859	0.0314018	0.0237	0.1845	31.67055229
rs144801310	15	57469927	A	C	0.9661	-0.1006	0.0183	4.01E-08	0.965885	-0.0957953	0.0457	0.03596	30.21995282
rs145220772	10	114578143	T	C	0.0213	0.1315	0.0237	2.74E-08	0.0218974	0.0686021	0.0656	0.2953	30.78610978
rs145268310	3	12310773	C	G	0.1171	0.0606	0.0098	7.401E-10	0.114885	-0.0259026	0.0261	0.3219	38.23781758
rs146240813	10	93566126	T	C	0.0737	0.0763	0.0129	3.13E-09	0.0826464	-0.0942008	0.0331	0.004432	34.98401538

rs148407308	17	40565200	T	C	0.0472	0.0872	0.0151	7.312E-09	0.0406885	-0.0374008	0.0442	0.3971	33.34871278
rs151212	11	2690293	A	C	0.6422	-0.0481	0.0068	1.576E-12	0.663628	0.00459941	0.0183	0.802	50.03481834
rs1512993	12	71401019	A	G	0.4361	0.0359	0.0065	3.197E-08	0.436628	-0.0122042	0.0171	0.4745	30.5043787
rs17106184	1	50909985	A	G	0.0933	-0.062	0.0111	2.319E-08	0.0961205	0.00669752	0.0283	0.8129	31.19876633
rs17122772	14	23288935	C	G	0.7736	-0.0428	0.0077	3.313E-08	0.789974	0.0399032	0.0232	0.0862005	30.89627256
rs17179392	2	158309480	T	C	0.9562	0.0873	0.0159	4.124E-08	0.96	0.00019998	0.0427	0.9965	30.14631541
rs17712208	1	214150445	A	T	0.0345	0.1854	0.0185	1.462E-23	0.0302424	-0.0798986	0.0556	0.1511	100.4328999
rs17772814	8	128711742	A	G	0.0857	-0.0746	0.0125	2.133E-09	0.0705	-0.0041988	0.0415	0.9192	35.617024
rs180863361	10	114596377	T	C	0.9875	0.2313	0.0351	4.27E-11	0.991628	-0.0774968	0.1003	0.4392	43.42472058
rs189966089	10	114886341	T	C	0.029	-0.1284	0.0221	6.173E-09	0.0198398	-0.0786969	0.0719	0.2741	33.75557421
rs190054319	11	32595598	T	C	0.0554	-0.0815	0.0147	2.698E-08	0.056836	-0.0697038	0.0371	0.0600897	30.73834976
rs2005705	17	36096300	A	G	0.4416	0.0532	0.0066	7.312E-16	0.453231	0.0291024	0.0187	0.12	64.97337006
rs2010825	7	44188220	T	C	0.5097	-0.0382	0.0065	3.991E-09	0.512282	-0.045102	0.0173	0.00902007	34.53822485

rs2201103	3	63781484	T	C	0.1848	-0.0492	0.0084	4.259E-09	0.186487	0.0454035	0.022	0.0388803	34.30612245
rs223481	4	103688017	T	C	0.5186	0.035	0.0064	4.253E-08	0.508769	-0.0160989	0.0167	0.3343	29.90722656
rs2248082	10	71457566	C	G	0.401	-0.0401	0.0066	1.208E-09	0.394885	-0.0120017	0.0177	0.4985	36.91483012
rs2272662	8	145639726	T	C	0.4123	-0.0402	0.0072	2.63E-08	0.439513	-0.0154995	0.0216	0.473301	31.17361111
rs2456530	15	53091553	T	C	0.126	0.054	0.0096	2.071E-08	0.130256	-0.0247026	0.0253	0.3286	31.640625
rs2464592	8	118183551	A	G	0.7122	-0.0496	0.0071	3.249E-12	0.703	0.00880116	0.0183	0.631401	48.80301527
rs2506125	9	84293453	A	G	0.4134	-0.0609	0.0066	2.643E-20	0.410372	0.00410158	0.018	0.8187	85.14256198
rs2747567	20	32381337	A	G	0.4263	-0.0375	0.0067	2.191E-08	0.425026	-0.00670241	0.018	0.7113	31.32657607
rs2783019	6	107422239	C	G	0.3403	-0.0383	0.0068	1.827E-08	0.313141	-0.0255024	0.02	0.2008	31.72339965
rs2793823	1	120437718	A	G	0.1301	0.0697	0.0094	1.397E-13	0.126141	-0.0598986	0.0259	0.0205102	54.98064735
rs28663084	3	152382187	A	G	0.673	-0.0376	0.0068	3.298E-08	0.678769	-0.0297996	0.0179	0.0965295	30.57439446
rs28681372	22	50351977	A	G	0.5908	-0.0396	0.0069	1.001E-08	0.604282	-0.060504	0.0235	0.00994901	32.93761815
rs28819812	4	157652753	A	C	0.3218	-0.0396	0.0072	4.22E-08	0.339628	-0.0220002	0.0177	0.2135	30.25

rs2898655	17	36053105	T	C	0.084	-0.0663	0.0119	2.783E-08	0.0872758	-0.0426988	0.0319	0.1808	31.04081633
rs2925979	16	81534790	T	C	0.2987	0.0546	0.007	7.068E-15	0.293885	-0.0227979	0.0186	0.2201	60.84
rs297050	10	71306424	A	G	0.6441	0.0402	0.0067	1.984E-09	0.659372	0.00610135	0.0184	0.740099	36
rs3094515	17	36043653	T	C	0.3699	-0.0464	0.0072	1.339E-10	0.368513	-0.0267958	0.0194	0.1663	41.5308642
rs316623	15	41801081	T	C	0.4463	0.0392	0.0065	1.554E-09	0.443654	-0.0326988	0.0174	0.0597902	36.37017751
rs320369	1	118143517	A	G	0.3232	0.0372	0.0068	4.602E-08	0.307885	-0.00920221	0.0182	0.611999	29.92733564
rs325	8	19819328	T	C	0.9011	0.0677	0.0108	3.438E-10	0.898115	0.0394995	0.0279	0.1565	39.29432442
rs34238147	13	26776255	A	G	0.2287	-0.0419	0.0076	4.207E-08	0.228744	0.00980181	0.0201	0.625299	30.39490997
rs348330	1	229672955	A	G	0.6399	-0.0492	0.0067	2.1E-13	0.633231	0.045002	0.0179	0.01187	53.92381377
rs35339173	10	80904919	T	C	0.2421	0.045	0.0077	6.324E-09	0.252256	-0.0166986	0.0197	0.3957	34.15415753
rs3808636	8	41386465	T	G	0.0921	-0.0699	0.0115	1.286E-09	0.0938833	0.0251023	0.0325	0.4393	36.9452552
rs3809547	15	75628841	C	G	0.2409	-0.0417	0.0076	4.876E-08	0.266	-0.00569619	0.0194	0.7712	30.10543629
rs3816046	19	46118127	T	C	0.3238	0.0384	0.0069	2.746E-08	0.327487	0.0194987	0.0195	0.3195	30.97164461

rs3820981	2	165566877	A	G	0.5632	0.0402	0.0065	5.915E-10	0.569141	-0.0239956	0.0171	0.1603	38.24946746
rs3850071	13	80603592	A	C	0.6186	0.0457	0.0067	9.109E-12	0.621487	-0.00439966	0.0188	0.8134	46.52461573
rs3851525	6	7014062	T	G	0.4094	0.0356	0.0065	4.157E-08	0.411	-0.00559563	0.0171	0.743599	29.99668639
rs3862948	1	205099959	A	C	0.235	0.0414	0.0075	3.98E-08	0.231487	0.0142973	0.0198	0.4708	30.4704
rs40270	5	55804552	A	C	0.2366	-0.0683	0.0075	1.304E-19	0.228859	0.0101978	0.0201	0.611999	82.93137778
rs4238013	12	4376089	T	C	0.7896	-0.0566	0.008	1.136E-12	0.808487	0.0246048	0.0237	0.2979	50.055625
rs4422335	3	23187407	A	G	0.9	0.0707	0.0108	5.535E-11	0.899769	-0.0601004	0.0282	0.0331497	42.8539952
rs4457053	5	76424949	A	G	0.6983	-0.0591	0.007	3.616E-17	0.687628	-0.016597	0.0196	0.3962	71.28183673
rs4463188	5	44642670	T	C	0.6023	-0.0376	0.0065	6.949E-09	0.591769	-0.0278029	0.0175	0.1116	33.46177515
rs4466139	5	78430058	T	C	0.636	0.04	0.0066	1.327E-09	0.639487	0.0305001	0.0175	0.0810009	36.73094582
rs4659326	1	117525810	T	C	0.2366	-0.045	0.0075	2.383E-09	0.228372	0.0285	0.0201	0.1572	36
rs4679370	3	124919777	T	C	0.4605	-0.0365	0.0064	1.098E-08	0.465513	-0.0270013	0.0171	0.1146	32.52563477
rs4689381	4	6257188	T	C	0.4437	0.038	0.0066	8.369E-09	0.453744	0.00459941	0.0167	0.784899	33.1496786

rs4813428	20	21451848	T	C	0.096	0.0599	0.0109	3.778E-08	0.091836	-0.0446	0.0293	0.1276	30.19956233
rs4964654	12	108594043	A	T	0.3079	-0.0396	0.0069	1.001E-08	0.296654	-0.0513038	0.0194	0.00817805	32.93761815
rs520711	10	94220645	A	T	0.1909	0.0525	0.0082	1.289E-10	0.189282	-0.0393959	0.0216	0.0680597	40.99122546
rs55752756	4	185672745	A	G	0.1699	-0.0485	0.0086	1.614E-08	0.172628	-0.00240288	0.0228	0.9167	31.8043537
rs55883527	15	77854352	T	G	0.3209	0.0439	0.0068	1.115E-10	0.314487	0.0246048	0.0182	0.1773	41.67841696
rs55938323	10	114773887	T	C	0.9891	-0.2268	0.0374	1.298E-09	0.988	0.105899	0.0877	0.2271	36.77417141
rs56218834	2	25520857	A	G	0.4251	-0.0354	0.0065	4.946E-08	0.444461	0.0430978	0.017	0.0112499	29.66059172
rs5758223	22	41489920	A	G	0.7134	0.0401	0.0071	1.779E-08	0.721487	0.0133998	0.0187	0.4732	31.89863122
rs6015379	20	57386639	A	G	0.4528	-0.0411	0.0064	1.234E-10	0.471398	-0.0187955	0.0168	0.2631	41.24047852
rs6020373	20	48837791	T	C	0.6137	-0.036	0.0066	4.825E-08	0.622513	-0.0259026	0.0181	0.1525	29.75206612
rs61875108	10	114725079	A	G	0.0488	-0.093	0.0155	1.954E-09	0.0496974	0.0148985	0.0456	0.744	36
rs62048489	16	53454855	T	G	0.0893	-0.0711	0.0124	8.674E-09	0.0835769	0.00410158	0.0358	0.9084	32.87727627
rs62066054	17	9782028	A	G	0.3221	0.0379	0.0068	2.564E-08	0.303372	0.0217028	0.0182	0.2342	31.0642301

rs62290256	3	185256242	A	G	0.9364	-0.0768	0.0134	1.001E-08	0.948256	-0.0798986	0.0424	0.0593103	32.84829583
rs62294592	3	186635584	T	C	0.1236	-0.054	0.0098	4.096E-08	0.112	0.00870203	0.0271	0.7473	30.36234902
rs62451124	7	27968345	A	G	0.0573	-0.0801	0.0141	1.454E-08	0.0496167	0.00509699	0.0392	0.8965	32.27206881
rs6459730	7	156916130	A	T	0.133	-0.0595	0.0097	9.931E-10	0.128487	0.0428008	0.0279	0.1252	37.62620895
rs6483215	11	92792115	A	G	0.233	0.0412	0.0074	2.987E-08	0.228115	0.0285	0.02	0.1542	30.99780862
rs6540807	1	214097482	T	C	0.5704	0.0349	0.0064	4.646E-08	0.557769	-0.00339576	0.017	0.8419	29.73657227
rs66930764	6	164103243	A	G	0.1288	-0.0532	0.0096	3.334E-08	0.131744	0.0332994	0.025	0.1819	30.71006944
rs672271	9	3273781	T	C	0.9062	-0.06	0.011	4.825E-08	0.904628	-0.00160128	0.0291	0.9561	29.75206612
rs67721004	5	53290571	T	C	0.741	-0.0484	0.0072	2.092E-11	0.740141	-0.0332983	0.0192	0.0827409	45.1882716
rs6829631	4	1781686	T	C	0.491	-0.0525	0.0066	1.733E-15	0.494602	0.045002	0.021	0.0326498	63.27479339
rs687621	9	136137065	A	G	0.6533	-0.0442	0.0071	5.364E-10	0.653424	-0.039604	0.0176	0.0242198	38.75500893
rs6918311	6	137287702	A	G	0.532	0.0445	0.0065	7.128E-12	0.52	0.00469894	0.0171	0.7826	46.86982249
rs7110293	11	34778351	A	C	0.1353	0.0509	0.0093	4.702E-08	0.134628	-0.0323992	0.0245	0.1864	29.9550237

rs7119618	11	128226431	C	G	0.7173	-0.0386	0.007	3.746E-08	0.721487	0.0232965	0.0186	0.2094	30.40734694
rs7146002	14	91871672	T	G	0.5729	-0.0358	0.0065	3.491E-08	0.584372	0.0107025	0.0171	0.5316	30.33467456
rs7234998	18	7068724	T	C	0.3736	0.0364	0.0066	3.423E-08	0.367628	-0.00940408	0.0178	0.5949	30.41689624
rs7250869	19	33887405	T	C	0.3129	0.0379	0.0069	4.146E-08	0.319	-0.00549507	0.0182	0.759799	30.17034237
rs72655474	9	22139684	C	G	0.0217	-0.1908	0.025	2.446E-14	0.0207026	-0.0187038	0.0749	0.8028	58.247424
rs72802340	16	75232528	T	G	0.0418	-0.1307	0.017	1.303E-14	0.0327115	0.0366019	0.0579	0.5278	59.10896194
rs72940580	18	52848102	A	G	0.9529	-0.0842	0.0154	4.484E-08	0.962	0.00019998	0.045	0.996	29.89391128
rs72999033	19	19366632	T	C	0.0659	0.0837	0.013	1.142E-10	0.0638487	-0.002002	0.0348	0.9553	41.45378698
rs7313918	12	118394008	T	C	0.8635	-0.0559	0.0094	2.99E-09	0.867628	-0.00260339	0.026	0.92	35.36453146
rs73390986	22	30135928	T	C	0.9088	0.0764	0.0113	1.415E-11	0.915487	0.0171029	0.0304	0.575	45.71195865
rs7433808	3	64727086	A	T	0.7617	0.0505	0.0075	2.1E-11	0.738885	-0.000900405	0.0194	0.9639	45.33777778
rs7568063	2	226840831	T	C	0.8392	0.064	0.009	1.181E-12	0.840744	-0.00329542	0.0268	0.9025	50.56790123
rs76074250	11	2073182	A	G	0.7971	-0.0463	0.0081	9.305E-09	0.795372	-0.0041988	0.0214	0.8434	32.67322055

rs76079449	12	121425452	T	C	0.0226	0.1194	0.0218	4.192E-08	0.0248346	0.0698991	0.0565	0.2159	29.99823247
rs7615486	3	185308495	A	C	0.9655	-0.0989	0.0177	2.281E-08	0.924372	-0.0113036	0.0385	0.7694	31.22094545
rs7615580	3	12027240	T	C	0.9344	0.1052	0.0131	9.13E-16	0.937372	0.0238044	0.0346	0.491501	64.48948196
rs76367336	2	227228251	T	C	0.8863	0.0584	0.0103	1.259E-08	0.874141	-0.0208968	0.026	0.4225	32.14779904
rs7646490	3	122936084	A	G	0.2113	-0.054	0.008	1.152E-11	0.206513	-0.023197	0.021	0.2692	45.5625
rs77460585	5	101123995	A	G	0.0486	0.0878	0.0152	7.328E-09	0.0423706	-0.0382004	0.0432	0.3757	33.3658241
rs77655131	7	102086552	T	C	0.1301	0.0554	0.0097	1.274E-08	0.109885	-0.034105	0.0299	0.2532	32.61940695
rs7775748	6	126623947	T	C	0.4487	0.0399	0.0065	7.923E-10	0.450256	-0.0230025	0.0173	0.1841	37.68071006
rs78011501	12	121097000	A	G	0.0449	0.0913	0.0155	3.819E-09	0.0439807	0.0254046	0.0418	0.5441	34.6959001
rs78683917	2	65562302	T	C	0.9729	0.1115	0.0203	4.035E-08	0.97	0.00320486	0.0497	0.9493	30.16877381
rs7897943	10	12239456	A	G	0.7531	-0.0478	0.0074	1.276E-10	0.760372	0.0119977	0.0198	0.5433	41.72461651
rs8030349	15	90362275	A	G	0.3523	0.0412	0.0069	2.491E-09	0.345654	-0.0383979	0.0202	0.0566996	35.65301407
rs8032939	15	38834033	T	C	0.7529	-0.0418	0.0074	1.878E-08	0.740654	-0.0334017	0.0192	0.0823304	31.90723156

rs9309324	2	60546132	T	G	0.4857	0.0414	0.0064	9.047E-11	0.498256	-0.0164953	0.017	0.3323	41.84472656
rs9316500	13	51094114	T	G	0.7121	0.0387	0.007	3.454E-08	0.696	-0.00359646	0.0182	0.8451	30.56510204
rs9379084	6	7231843	A	G	0.1121	-0.0994	0.0106	5.478E-21	0.137256	-0.0128016	0.0274	0.639401	87.93485226
rs9520799	13	108781439	A	T	0.859	-0.0509	0.0092	3.308E-08	0.850282	-0.050704	0.0237	0.0320398	30.60975898

Abbreviation: T2DM= type 2 diabetes mellitus, IBD= inflammatory bowel disease, SNPs= single-nucleotide polymorphisms, Chr= Chromosome, EA= Effect Allele, OA= Other Allele, EAF= effect allele frequency, SE= standard error.

Table S14. Characteristics of the SNPs related to T2DM and UC

SNP	Chr	Position	EA	OA	Exposure: T2DM				Outcome: UC				F statistic
					EAF	β	SE	*P*	EAF	β	SE	*P*	
rs10033601	4	153252061	A	G	0.7138	0.0451	0.0071	2.381E-10	0.706222	0.00669752	0.0239	0.779701	40.34933545
rs1025216	3	168212861	A	G	0.1278	-0.0543	0.0096	1.729E-08	0.126746	-0.00190181	0.0319	0.9525	31.99316406
rs10260837	7	28181129	T	C	0.1111	0.0577	0.0102	1.337E-08	0.114492	-0.015205	0.0333	0.6467	32.00009612
rs1043246	17	3828086	C	G	0.8445	-0.0566	0.0096	4.206E-09	0.874492	-0.0275048	0.0413	0.5052	34.76085069
rs10502791	18	40071369	A	T	0.7888	-0.0426	0.0077	3.837E-08	0.78873	-0.0458031	0.0257	0.0742096	30.608197
rs10509406	10	80959517	T	C	0.8277	0.0564	0.0086	5.061E-11	0.825492	-0.0136021	0.0284	0.633	43.00919416
rs10771260	12	26253557	A	C	0.6113	-0.0367	0.0066	2.64E-08	0.612508	0.00509699	0.0218	0.8137	30.92033976
rs10821310	9	96928812	T	C	0.3193	0.0372	0.0068	4.602E-08	0.317762	0.0263011	0.0231	0.2553	29.92733564
rs10848958	12	4031104	T	C	0.1966	-0.0451	0.0083	4.968E-08	0.189	0.0077995	0.0269	0.7724	29.5254754
rs10853047	17	62199136	T	C	0.8627	-0.0519	0.0094	3.636E-08	0.854778	0.0109993	0.0304	0.716401	30.48449525

rs10965199	9	21954653	T	C	0.0361	-0.1139	0.019	1.853E-09	0.0499222	0.0262037	0.0522	0.616	35.93686981
rs11049086	12	27818770	A	G	0.1948	-0.046	0.0082	1.775E-08	0.192524	0.0244975	0.0269	0.3617	31.46936347
rs1105291	15	91502383	T	C	0.1429	0.0613	0.0091	1.702E-11	0.136254	-0.00849599	0.0316	0.788199	45.37724912
rs11063029	12	4301301	T	C	0.0581	0.0858	0.0138	5.364E-10	0.0580302	0.0515008	0.0462	0.2648	38.65595463
rs11108086	12	95914758	T	C	0.9326	-0.0697	0.0128	4.855E-08	0.934254	0.0114047	0.043	0.7905	29.65142822
rs11122800	2	121305604	A	G	0.8534	0.0571	0.0091	3.647E-10	0.854238	-0.0037972	0.0301	0.8991	39.37217727
rs111620997	12	66162603	A	G	0.9513	-0.0857	0.0157	4.85E-08	0.957508	-0.00210221	0.0657	0.9746	29.79630005
rs111686785	5	14738965	A	G	0.9694	-0.1169	0.0194	1.588E-09	0.975	0.00229736	0.0874	0.9788	36.30994261
rs11235566	11	72403388	T	C	0.5456	0.0353	0.0064	3.258E-08	0.544746	-0.00450011	0.0213	0.8321	30.42211914
rs112538930	7	14859137	T	C	0.1843	0.0605	0.0083	2.588E-13	0.171286	0.0700017	0.0284	0.01378	53.13180433
rs11514705	7	15016108	T	C	0.3309	0.0377	0.0069	4.881E-08	0.331	0.00149888	0.0252	0.9537	29.85276202
rs11564714	11	2190418	C	G	0.0855	0.0715	0.0121	3.934E-09	0.0873348	-0.0806029	0.0474	0.089041	34.91735537
rs11655029	17	17649172	T	C	0.3207	0.0463	0.0069	2.083E-11	0.326	0.0155977	0.0226	0.492	45.02604495

rs11680058	2	16574669	A	G	0.8648	0.0581	0.0104	2.081E-08	0.863254	0.0034042	0.0372	0.9281	31.20941198
rs116859590	10	114752410	T	C	0.0263	0.229	0.021	1.415E-27	0.0306602	-0.114099	0.0765	0.1359	118.9138322
rs116964396	8	41505849	A	C	0.0339	0.107	0.0188	1.367E-08	0.0318856	-0.142105	0.0643	0.02713	32.39305115
rs117316450	11	14518419	C	G	0.9803	-0.1274	0.0232	4.229E-08	0.978254	-0.0123965	0.0746	0.8677	30.15524673
rs117465240	11	69444235	A	G	0.0319	-0.1053	0.0193	4.608E-08	0.039773	0.0940002	0.0645	0.145	29.76748369
rs117753849	10	114603431	A	G	0.9832	-0.1924	0.0252	2.443E-14	0.982508	0.116102	0.084	0.1668	58.2920131
rs117987174	10	114756459	A	G	0.0135	-0.1723	0.0305	1.558E-08	0.00696698	-0.00540458	0.1537	0.9719	31.91323838
rs11964747	6	20485898	T	C	0.1861	-0.0577	0.0083	3.042E-12	0.184286	0.0580991	0.0283	0.0400904	48.32762375
rs12154627	7	130422934	T	C	0.5045	0.0469	0.0065	5.019E-13	0.521222	-0.0270013	0.0216	0.2115	52.06177515
rs12222793	11	92667047	A	G	0.5465	-0.0434	0.0066	4.72E-11	0.537746	-0.00920221	0.0229	0.6877	43.2405877
rs12419690	11	45858584	A	G	0.5524	-0.0351	0.0064	3.892E-08	0.567286	0.0560967	0.0215	0.00894994	30.07836914
rs12523853	6	20429804	T	C	0.0401	0.0973	0.0169	7.831E-09	0.0382476	-0.0156011	0.0614	0.7999	33.14761388
rs12625671	20	42994812	T	C	0.8849	-0.0757	0.0101	5.025E-14	0.882746	-0.00270365	0.0337	0.936	56.17576708

rs12640250	4	17792869	A	C	0.2834	-0.04	0.0071	1.93E-08	0.276746	-0.0226035	0.0238	0.3423	31.73973418
rs12680202	8	12617942	A	T	0.3206	0.0411	0.0071	7.803E-09	0.310762	-0.0122042	0.0245	0.619901	33.50942273
rs12703101	7	150504840	T	C	0.714	-0.04	0.007	1.184E-08	0.702476	-0.0123965	0.0232	0.5927	32.65306122
rs12974635	19	4947056	T	C	0.2016	0.0452	0.0081	2.065E-08	0.197016	0.0173978	0.0269	0.5191	31.13915562
rs13099586	3	23294322	T	C	0.0904	-0.0634	0.0113	2.062E-08	0.0830746	-0.0329985	0.0389	0.3969	31.47905083
rs13267392	8	145245647	T	C	0.2773	0.047	0.0077	1.311E-09	0.253016	0.0369007	0.0304	0.2248	37.25754765
rs13293121	9	81881830	A	G	0.9316	0.0864	0.0134	1.142E-10	0.942222	-0.0329985	0.046	0.4738	41.57362442
rs138960826	3	185530177	A	G	0.0479	0.0877	0.0154	1.212E-08	0.0451158	-0.0570043	0.0571	0.318	32.43080621
rs1436955	15	62404382	T	C	0.2718	-0.0394	0.0072	4.933E-08	0.263016	-0.00690378	0.024	0.7738	29.94521605
rs144102357	11	127999754	A	G	0.1361	-0.0529	0.0094	1.979E-08	0.14727	0.0509972	0.0301	0.0904795	31.67055229
rs144801310	15	57469927	A	C	0.9661	-0.1006	0.0183	4.01E-08	0.966238	-0.104306	0.0572	0.0684006	30.21995282
rs145220772	10	114578143	T	C	0.0213	0.1315	0.0237	2.74E-08	0.0224064	0.0526968	0.0791	0.5051	30.78610978
rs145268310	3	12310773	C	G	0.1171	0.0606	0.0098	7.401E-10	0.116	-0.00429923	0.0327	0.8961	38.23781758

rs146240813	10	93566126	T	C	0.0737	0.0763	0.0129	3.13E-09	0.0828583	-0.112195	0.042	0.007465	34.98401538
rs148407308	17	40565200	T	C	0.0472	0.0872	0.0151	7.312E-09	0.0407238	-0.0750038	0.0561	0.1814	33.34871278
rs151212	11	2690293	A	C	0.6422	-0.0481	0.0068	1.576E-12	0.661492	0.0199007	0.0229	0.3852	50.03481834
rs1512993	12	71401019	A	G	0.4361	0.0359	0.0065	3.197E-08	0.435746	-0.0133994	0.0215	0.5337	30.5043787
rs17106184	1	50909985	A	G	0.0933	-0.062	0.0111	2.319E-08	0.095219	-0.0176956	0.036	0.622401	31.19876633
rs17122772	14	23288935	C	G	0.7736	-0.0428	0.0077	3.313E-08	0.787524	0.00689617	0.0292	0.8142	30.89627256
rs17179392	2	158309480	T	C	0.9562	0.0873	0.0159	4.124E-08	0.959238	-0.0444013	0.0527	0.4001	30.14631541
rs17712208	1	214150445	A	T	0.0345	0.1854	0.0185	1.462E-23	0.0307476	-0.0559039	0.0665	0.4007	100.4328999
rs17772814	8	128711742	A	G	0.0857	-0.0746	0.0125	2.133E-09	0.0711826	0.018596	0.0517	0.719399	35.617024
rs180863361	10	114596377	T	C	0.9875	0.2313	0.0351	4.27E-11	0.992	-0.115905	0.125	0.354	43.42472058
rs189966089	10	114886341	T	C	0.029	-0.1284	0.0221	6.173E-09	0.0201206	-0.130599	0.0919	0.1553	33.75557421
rs190054319	11	32595598	T	C	0.0554	-0.0815	0.0147	2.698E-08	0.0577254	-0.0425005	0.0464	0.3598	30.73834976
rs2005705	17	36096300	A	G	0.4416	0.0532	0.0066	7.312E-16	0.451508	0.0338022	0.0231	0.1423	64.97337006

rs2010825	7	44188220	T	C	0.5097	-0.0382	0.0065	3.991E-09	0.514476	-0.034695	0.0219	0.1137	34.53822485
rs2201103	3	63781484	T	C	0.1848	-0.0492	0.0084	4.259E-09	0.187286	0.0874979	0.0277	0.001563	34.30612245
rs223481	4	103688017	T	C	0.5186	0.035	0.0064	4.253E-08	0.506936	-0.054604	0.0211	0.00967409	29.90722656
rs2248082	10	71457566	C	G	0.401	-0.0401	0.0066	1.208E-09	0.397762	-0.00640044	0.0222	0.772601	36.91483012
rs2272662	8	145639726	T	C	0.4123	-0.0402	0.0072	2.63E-08	0.438984	-0.0154995	0.0273	0.5699	31.17361111
rs2456530	15	53091553	T	C	0.126	0.054	0.0096	2.071E-08	0.129	-0.022399	0.032	0.4845	31.640625
rs2464592	8	118183551	A	G	0.7122	-0.0496	0.0071	3.249E-12	0.704778	0.0184979	0.0231	0.4241	48.80301527
rs2506125	9	84293453	A	G	0.4134	-0.0609	0.0066	2.643E-20	0.411508	-0.022399	0.0226	0.3217	85.14256198
rs2747567	20	32381337	A	G	0.4263	-0.0375	0.0067	2.191E-08	0.423698	-0.0313049	0.0227	0.1669	31.32657607
rs2783019	6	107422239	C	G	0.3403	-0.0383	0.0068	1.827E-08	0.315508	0.00750179	0.0249	0.7619	31.72339965
rs2793823	1	120437718	A	G	0.1301	0.0697	0.0094	1.397E-13	0.127	-0.0307995	0.0323	0.3408	54.98064735
rs28663084	3	152382187	A	G	0.673	-0.0376	0.0068	3.298E-08	0.67846	-0.0331019	0.0226	0.1422	30.57439446
rs28681372	22	50351977	A	G	0.5908	-0.0396	0.0069	1.001E-08	0.604952	-0.0781021	0.0296	0.00819804	32.93761815

rs28819812	4	157652753	A	C	0.3218	-0.0396	0.0072	4.22E-08	0.337476	-0.034695	0.0224	0.1208	30.25
rs2898655	17	36053105	T	C	0.084	-0.0663	0.0119	2.783E-08	0.0886332	-0.023801	0.04	0.5524	31.04081633
rs2925979	16	81534790	T	C	0.2987	0.0546	0.007	7.068E-15	0.296492	-0.025205	0.0232	0.2779	60.84
rs297050	10	71306424	A	G	0.6441	0.0402	0.0067	1.984E-09	0.658492	0.00850374	0.0232	0.7133	36
rs3094515	17	36043653	T	C	0.3699	-0.0464	0.0072	1.339E-10	0.373	-0.0212032	0.0251	0.3979	41.5308642
rs316623	15	41801081	T	C	0.4463	0.0392	0.0065	1.554E-09	0.44319	-0.0505988	0.0217	0.01992	36.37017751
rs320369	1	118143517	A	G	0.3232	0.0372	0.0068	4.602E-08	0.309508	-0.014596	0.0229	0.5242	29.92733564
rs325	8	19819328	T	C	0.9011	0.0677	0.0108	3.438E-10	0.89827	0.0353961	0.0355	0.3183	39.29432442
rs34238147	13	26776255	A	G	0.2287	-0.0419	0.0076	4.207E-08	0.230794	0.0385955	0.0252	0.1261	30.39490997
rs348330	1	229672955	A	G	0.6399	-0.0492	0.0067	2.1E-13	0.633794	0.0416984	0.0228	0.06663	53.92381377
rs35339173	10	80904919	T	C	0.2421	0.045	0.0077	6.324E-09	0.249968	-0.0281012	0.0248	0.2573	34.15415753
rs3808636	8	41386465	T	G	0.0921	-0.0699	0.0115	1.286E-09	0.0933206	0.00269636	0.041	0.9474	36.9452552
rs3809547	15	75628841	C	G	0.2409	-0.0417	0.0076	4.876E-08	0.261206	-0.0135007	0.0247	0.583399	30.10543629

rs3816046	19	46118127	T	C	0.3238	0.0384	0.0069	2.746E-08	0.329746	0.00229736	0.0248	0.9273	30.97164461
rs3820981	2	165566877	A	G	0.5632	0.0402	0.0065	5.915E-10	0.56973	-0.0122953	0.0215	0.5659	38.24946746
rs3850071	13	80603592	A	C	0.6186	0.0457	0.0067	9.109E-12	0.62127	0.00919757	0.0231	0.6909	46.52461573
rs3851525	6	7014062	T	G	0.4094	0.0356	0.0065	4.157E-08	0.410762	0.0168964	0.0215	0.4311	29.99668639
rs3862948	1	205099959	A	C	0.235	0.0414	0.0075	3.98E-08	0.230762	0.0223973	0.025	0.3718	30.4704
rs40270	5	55804552	A	C	0.2366	-0.0683	0.0075	1.304E-19	0.229032	0.0176041	0.0252	0.4861	82.93137778
rs4238013	12	4376089	T	C	0.7896	-0.0566	0.008	1.136E-12	0.80827	0.0274984	0.0297	0.3531	50.055625
rs4422335	3	23187407	A	G	0.9	0.0707	0.0108	5.535E-11	0.901238	-0.0203048	0.0355	0.5687	42.8539952
rs4457053	5	76424949	A	G	0.6983	-0.0591	0.007	3.616E-17	0.689508	-0.0377953	0.0249	0.129	71.28183673
rs4463188	5	44642670	T	C	0.6023	-0.0376	0.0065	6.949E-09	0.592746	-0.0175023	0.0218	0.4211	33.46177515
rs4466139	5	78430058	T	C	0.636	0.04	0.0066	1.327E-09	0.63854	0.0565032	0.022	0.0102	36.73094582
rs4659326	1	117525810	T	C	0.2366	-0.045	0.0075	2.383E-09	0.230778	0.0485044	0.0251	0.0532304	36
rs4679370	3	124919777	T	C	0.4605	-0.0365	0.0064	1.098E-08	0.46446	-0.0293979	0.0213	0.1675	32.52563477

rs4689381	4	6257188	T	C	0.4437	0.038	0.0066	8.369E-09	0.453	0.00330453	0.0211	0.876	33.1496786
rs4813428	20	21451848	T	C	0.096	0.0599	0.0109	3.778E-08	0.0928254	-0.00450011	0.0367	0.9028	30.19956233
rs4964654	12	108594043	A	T	0.3079	-0.0396	0.0069	1.001E-08	0.298222	-0.0369026	0.0245	0.1327	32.93761815
rs520711	10	94220645	A	T	0.1909	0.0525	0.0082	1.289E-10	0.189206	-0.0364047	0.0272	0.181	40.99122546
rs55752756	4	185672745	A	G	0.1699	-0.0485	0.0086	1.614E-08	0.171984	-0.0138961	0.0285	0.625101	31.8043537
rs55883527	15	77854352	T	G	0.3209	0.0439	0.0068	1.115E-10	0.314762	0.0153023	0.0229	0.505	41.67841696
rs55938323	10	114773887	T	C	0.9891	-0.2268	0.0374	1.298E-09	0.987746	0.181496	0.1114	0.1031	36.77417141
rs56218834	2	25520857	A	G	0.4251	-0.0354	0.0065	4.946E-08	0.44354	0.0556996	0.0213	0.00888301	29.66059172
rs5758223	22	41489920	A	G	0.7134	0.0401	0.0071	1.779E-08	0.719254	0.0107025	0.0235	0.6505	31.89863122
rs6015379	20	57386639	A	G	0.4528	-0.0411	0.0064	1.234E-10	0.469444	-0.0236986	0.0211	0.2602	41.24047852
rs6020373	20	48837791	T	C	0.6137	-0.036	0.0066	4.825E-08	0.623508	0.0141001	0.0227	0.5324	29.75206612
rs60980157	9	139235415	T	C	0.2459	-0.0727	0.0082	5.489E-19	0.236064	0.103801	0.0314	0.000958606	78.60336109
rs61875108	10	114725079	A	G	0.0488	-0.093	0.0155	1.954E-09	0.0496254	-0.00559563	0.0574	0.9224	36

rs62048489	16	53454855	T	G	0.0893	-0.0711	0.0124	8.674E-09	0.0834492	0.0154008	0.0447	0.730601	32.87727627
rs62066054	17	9782028	A	G	0.3221	0.0379	0.0068	2.564E-08	0.305794	0.0491996	0.0228	0.0309899	31.0642301
rs62290256	3	185256242	A	G	0.9364	-0.0768	0.0134	1.001E-08	0.949	-0.0312018	0.0538	0.5613	32.84829583
rs62294592	3	186635584	T	C	0.1236	-0.054	0.0098	4.096E-08	0.113016	0.0111971	0.0339	0.7411	30.36234902
rs62451124	7	27968345	A	G	0.0573	-0.0801	0.0141	1.454E-08	0.0510016	0.0171029	0.0486	0.724399	32.27206881
rs6459730	7	156916130	A	T	0.133	-0.0595	0.0097	9.931E-10	0.127762	0.0469027	0.0349	0.1787	37.62620895
rs6483215	11	92792115	A	G	0.233	0.0412	0.0074	2.987E-08	0.22827	0.0195967	0.0251	0.436	30.99780862
rs6540807	1	214097482	T	C	0.5704	0.0349	0.0064	4.646E-08	0.561	0.0124028	0.0215	0.565599	29.73657227
rs66930764	6	164103243	A	G	0.1288	-0.0532	0.0096	3.334E-08	0.131508	0.0371994	0.0313	0.2354	30.71006944
rs672271	9	3273781	T	C	0.9062	-0.06	0.011	4.825E-08	0.904	-0.0203048	0.0365	0.5777	29.75206612
rs67721004	5	53290571	T	C	0.741	-0.0484	0.0072	2.092E-11	0.741222	-0.0433982	0.024	0.0713806	45.1882716
rs6829631	4	1781686	T	C	0.491	-0.0525	0.0066	1.733E-15	0.495778	0.0301994	0.0265	0.2541	63.27479339
rs687621	9	136137065	A	G	0.6533	-0.0442	0.0071	5.364E-10	0.655206	-0.0144032	0.0221	0.5146	38.75500893

rs6918311	6	137287702	A	G	0.532	0.0445	0.0065	7.128E-12	0.522794	0.0315956	0.0215	0.1426	46.86982249
rs7110293	11	34778351	A	C	0.1353	0.0509	0.0093	4.702E-08	0.135508	-0.0166986	0.0309	0.5884	29.9550237
rs7119618	11	128226431	C	G	0.7173	-0.0386	0.007	3.746E-08	0.720254	0.0117013	0.0234	0.617	30.40734694
rs7146002	14	91871672	T	G	0.5729	-0.0358	0.0065	3.491E-08	0.583746	0.0057037	0.0215	0.791099	30.33467456
rs7234998	18	7068724	T	C	0.3736	0.0364	0.0066	3.423E-08	0.370016	0.0010994	0.0223	0.9595	30.41689624
rs7250869	19	33887405	T	C	0.3129	0.0379	0.0069	4.146E-08	0.32027	0.0247999	0.0228	0.277	30.17034237
rs72655474	9	22139684	C	G	0.0217	-0.1908	0.025	2.446E-14	0.020592	-0.0546991	0.0944	0.5623	58.247424
rs72802340	16	75232528	T	G	0.0418	-0.1307	0.017	1.303E-14	0.0325016	0.0195967	0.0712	0.782899	59.10896194
rs72940580	18	52848102	A	G	0.9529	-0.0842	0.0154	4.484E-08	0.960238	-0.00389759	0.0554	0.944	29.89391128
rs72999033	19	19366632	T	C	0.0659	0.0837	0.013	1.142E-10	0.0650636	-0.0289964	0.0438	0.5079	41.45378698
rs7313918	12	118394008	T	C	0.8635	-0.0559	0.0094	2.99E-09	0.868254	0.0246048	0.0327	0.4515	35.36453146
rs73390986	22	30135928	T	C	0.9088	0.0764	0.0113	1.415E-11	0.914524	0.0489997	0.0384	0.2019	45.71195865
rs7433808	3	64727086	A	T	0.7617	0.0505	0.0075	2.1E-11	0.740508	-0.0199986	0.0245	0.4149	45.33777778

rs7568063	2	226840831	T	C	0.8392	0.064	0.009	1.181E-12	0.841	0.00350385	0.0342	0.9196	50.56790123
rs76074250	11	2073182	A	G	0.7971	-0.0463	0.0081	9.305E-09	0.795508	-0.0145047	0.0268	0.5881	32.67322055
rs76079449	12	121425452	T	C	0.0226	0.1194	0.0218	4.192E-08	0.025689	0.1684	0.0684	0.0138401	29.99823247
rs7615486	3	185308495	A	C	0.9655	-0.0989	0.0177	2.281E-08	0.923508	-0.0456042	0.0481	0.3432	31.22094545
rs7615580	3	12027240	T	C	0.9344	0.1052	0.0131	9.13E-16	0.936254	0.0227004	0.0436	0.6023	64.48948196
rs76367336	2	227228251	T	C	0.8863	0.0584	0.0103	1.259E-08	0.875492	-0.0232993	0.0329	0.4775	32.14779904
rs7646490	3	122936084	A	G	0.2113	-0.054	0.008	1.152E-11	0.208746	-0.0144032	0.0263	0.582599	45.5625
rs77460585	5	101123995	A	G	0.0486	0.0878	0.0152	7.328E-09	0.0421332	-0.111602	0.0548	0.0417398	33.3658241
rs77655131	7	102086552	T	C	0.1301	0.0554	0.0097	1.274E-08	0.111508	-0.0127003	0.0378	0.7373	32.61940695
rs7775748	6	126623947	T	C	0.4487	0.0399	0.0065	7.923E-10	0.449984	-0.0144032	0.0218	0.5083	37.68071006
rs78011501	12	121097000	A	G	0.0449	0.0913	0.0155	3.819E-09	0.0441382	0.0603046	0.0517	0.2428	34.6959001
rs78683917	2	65562302	T	C	0.9729	0.1115	0.0203	4.035E-08	0.970746	-0.0608015	0.0623	0.329	30.16877381
rs7897943	10	12239456	A	G	0.7531	-0.0478	0.0074	1.276E-10	0.760762	0.0285	0.0248	0.2506	41.72461651

rs8030349	15	90362275	A	G	0.3523	0.0412	0.0069	2.491E-09	0.347222	-0.0423962	0.0259	0.101	35.65301407
rs8032939	15	38834033	T	C	0.7529	-0.0418	0.0074	1.878E-08	0.744254	0.0145043	0.0244	0.5514	31.90723156
rs9309324	2	60546132	T	G	0.4857	0.0414	0.0064	9.047E-11	0.499476	-0.0263953	0.0214	0.2181	41.84472656
rs9316500	13	51094114	T	G	0.7121	0.0387	0.007	3.454E-08	0.697794	0.0172995	0.023	0.4523	30.56510204
rs9379084	6	7231843	A	G	0.1121	-0.0994	0.0106	5.478E-21	0.13673	-0.010697	0.0346	0.756901	87.93485226
rs9520799	13	108781439	A	T	0.859	-0.0509	0.0092	3.308E-08	0.852984	-0.0408012	0.03	0.1742	30.60975898

Abbreviation: T2DM= type 2 diabetes mellitus, UC= ulcerative colitis, SNPs= single-nucleotide polymorphisms, Chr= Chromosome, EA= Effect Allele, OA= Other Allele, EAF= effect allele frequency, SE= standard error.

Table S15. Characteristics of the SNPs related to T2DM and CD

SNP	Chr	Position	EA	OA	Exposure: T2DM				Outcome: CD				F statistic
					EAF	β	SE	*P*	EAF	β	SE	*P*	
rs10033601	4	153252061	A	G	0.7138	0.0451	0.0071	2.381E-10	0.706859	0.00479847	0.0256	0.8497	40.34933545
rs1025216	3	168212861	A	G	0.1278	-0.0543	0.0096	1.729E-08	0.129	0.000699755	0.0342	0.9844	31.99316406
rs10260837	7	28181129	T	C	0.1111	0.0577	0.0102	1.337E-08	0.118282	0.0762015	0.035	0.0294802	32.00009612
rs1043246	17	3828086	C	G	0.8445	-0.0566	0.0096	4.206E-09	0.871289	-0.0660971	0.044	0.1334	34.76085069
rs10502791	18	40071369	A	T	0.7888	-0.0426	0.0077	3.837E-08	0.788856	0.00479847	0.0278	0.864	30.608197
rs10509406	10	80959517	T	C	0.8277	0.0564	0.0086	5.061E-11	0.819004	-0.0305004	0.0296	0.3028	43.00919416
rs10771260	12	26253557	A	C	0.6113	-0.0367	0.0066	2.64E-08	0.613715	-0.0158957	0.0235	0.499001	30.92033976
rs10821310	9	96928812	T	C	0.3193	0.0372	0.0068	4.602E-08	0.31757	0.0140015	0.025	0.5751	29.92733564
rs10848958	12	4031104	T	C	0.1966	-0.0451	0.0083	4.968E-08	0.19057	0.0239021	0.0295	0.4183	29.5254754
rs10853047	17	62199136	T	C	0.8627	-0.0519	0.0094	3.636E-08	0.850715	0.0177024	0.0325	0.5855	30.48449525

rs10965199	9	21954653	T	C	0.0361	-0.1139	0.019	1.853E-09	0.0490127	0.116404	0.057	0.0411102	35.93686981
rs11049086	12	27818770	A	G	0.1948	-0.046	0.0082	1.775E-08	0.188574	-0.0204987	0.0295	0.4865	31.46936347
rs1105291	15	91502383	T	C	0.1429	0.0613	0.0091	1.702E-11	0.137282	0.0903981	0.0337	0.00731493	45.37724912
rs11063029	12	4301301	T	C	0.0581	0.0858	0.0138	5.364E-10	0.0572708	0.0594003	0.0502	0.2369	38.65595463
rs11108086	12	95914758	T	C	0.9326	-0.0697	0.0128	4.855E-08	0.935285	-0.00260339	0.0465	0.955	29.65142822
rs11122800	2	121305604	A	G	0.8534	0.0571	0.0091	3.647E-10	0.857426	0.0463014	0.0334	0.1656	39.37217727
rs111620997	12	66162603	A	G	0.9513	-0.0857	0.0157	4.85E-08	0.957285	0.0384993	0.0707	0.5857	29.79630005
rs111686785	5	14738965	A	G	0.9694	-0.1169	0.0194	1.588E-09	0.97543	-0.109	0.0944	0.2481	36.30994261
rs11235566	11	72403388	T	C	0.5456	0.0353	0.0064	3.258E-08	0.54343	-0.00889948	0.0228	0.6959	30.42211914
rs112538930	7	14859137	T	C	0.1843	0.0605	0.0083	2.588E-13	0.171285	-0.00680309	0.0318	0.8309	53.13180433
rs11514705	7	15016108	T	C	0.3309	0.0377	0.0069	4.881E-08	0.33143	-0.0249999	0.0276	0.3648	29.85276202
rs11564714	11	2190418	C	G	0.0855	0.0715	0.0121	3.934E-09	0.0879285	0.00960374	0.0506	0.849	34.91735537
rs11655029	17	17649172	T	C	0.3207	0.0463	0.0069	2.083E-11	0.331856	0.00479847	0.0244	0.843	45.02604495

rs11680058	2	16574669	A	G	0.8648	0.0581	0.0104	2.081E-08	0.864574	-0.0303974	0.043	0.4789	31.20941198
rs116859590	10	114752410	T	C	0.0263	0.229	0.021	1.415E-27	0.032543	0.00039992	0.0773	0.9963	118.9138322
rs116964396	8	41505849	A	C	0.0339	0.107	0.0188	1.367E-08	0.0300454	-0.181906	0.0723	0.0119001	32.39305115
rs117316450	11	14518419	C	G	0.9803	-0.1274	0.0232	4.229E-08	0.97757	0.0943006	0.083	0.2562	30.15524673
rs117465240	11	69444235	A	G	0.0319	-0.1053	0.0193	4.608E-08	0.0417292	0.0264959	0.0727	0.715899	29.76748369
rs117753849	10	114603431	A	G	0.9832	-0.1924	0.0252	2.443E-14	0.982	0.0311982	0.0905	0.7298	58.2920131
rs117987174	10	114756459	A	G	0.0135	-0.1723	0.0305	1.558E-08	0.00723014	-0.179605	0.1716	0.2952	31.91323838
rs11964747	6	20485898	T	C	0.1861	-0.0577	0.0083	3.042E-12	0.183285	0.00259663	0.0307	0.9331	48.32762375
rs12154627	7	130422934	T	C	0.5045	0.0469	0.0065	5.019E-13	0.52743	-0.0231049	0.0234	0.3222	52.06177515
rs12222793	11	92667047	A	G	0.5465	-0.0434	0.0066	4.72E-11	0.533715	-0.011597	0.0261	0.6562	43.2405877
rs12419690	11	45858584	A	G	0.5524	-0.0351	0.0064	3.892E-08	0.568996	0.0374981	0.0233	0.1082	30.07836914
rs12523853	6	20429804	T	C	0.0401	0.0973	0.0169	7.831E-09	0.0393834	0.115799	0.0639	0.0696707	33.14761388
rs12625671	20	42994812	T	C	0.8849	-0.0757	0.0101	5.025E-14	0.878715	0.0020978	0.0365	0.9531	56.17576708

rs12640250	4	17792869	A	C	0.2834	-0.04	0.0071	1.93E-08	0.274004	-0.0259026	0.026	0.3191	31.73973418
rs12680202	8	12617942	A	T	0.3206	0.0411	0.0071	7.803E-09	0.307	-0.014799	0.0266	0.5782	33.50942273
rs12703101	7	150504840	T	C	0.714	-0.04	0.007	1.184E-08	0.698578	-0.0364047	0.0254	0.152	32.65306122
rs12974635	19	4947056	T	C	0.2016	0.0452	0.0081	2.065E-08	0.202	-0.0154995	0.0297	0.6032	31.13915562
rs13099586	3	23294322	T	C	0.0904	-0.0634	0.0113	2.062E-08	0.0815437	-0.00589736	0.0416	0.887	31.47905083
rs13293121	9	81881830	A	G	0.9316	0.0864	0.0134	1.142E-10	0.94643	-0.0659048	0.0527	0.211	41.57362442
rs138960826	3	185530177	A	G	0.0479	0.0877	0.0154	1.212E-08	0.0472123	0.0614995	0.0579	0.2878	32.43080621
rs1436955	15	62404382	T	C	0.2718	-0.0394	0.0072	4.933E-08	0.262141	0.0240973	0.0259	0.3507	29.94521605
rs144102357	11	127999754	A	G	0.1361	-0.0529	0.0094	1.979E-08	0.152141	-0.00240288	0.0319	0.9405	31.67055229
rs144801310	15	57469927	A	C	0.9661	-0.1006	0.0183	4.01E-08	0.967144	-0.135098	0.0624	0.0303697	30.21995282
rs145220772	10	114578143	T	C	0.0213	0.1315	0.0237	2.74E-08	0.0202563	0.175297	0.095	0.06501	30.78610978
rs145268310	3	12310773	C	G	0.1171	0.0606	0.0098	7.401E-10	0.114574	-0.0403013	0.0359	0.2615	38.23781758
rs146240813	10	93566126	T	C	0.0737	0.0763	0.0129	3.13E-09	0.0853877	-0.0499048	0.0447	0.2648	34.98401538

rs148407308	17	40565200	T	C	0.0472	0.0872	0.0151	7.312E-09	0.0402141	-0.0110003	0.0595	0.8529	33.34871278
rs151212	11	2690293	A	C	0.6422	-0.0481	0.0068	1.576E-12	0.667715	-0.00439966	0.0251	0.8605	50.03481834
rs1512993	12	71401019	A	G	0.4361	0.0359	0.0065	3.197E-08	0.436285	-0.0010005	0.0232	0.9668	30.5043787
rs17106184	1	50909985	A	G	0.0933	-0.062	0.0111	2.319E-08	0.0979271	0.0275957	0.0378	0.4657	31.19876633
rs17122772	14	23288935	C	G	0.7736	-0.0428	0.0077	3.313E-08	0.789708	0.0822986	0.0319	0.00989601	30.89627256
rs17179392	2	158309480	T	C	0.9562	0.0873	0.0159	4.124E-08	0.961141	0.0744022	0.0602	0.2172	30.14631541
rs17712208	1	214150445	A	T	0.0345	0.1854	0.0185	1.462E-23	0.0290437	-0.149998	0.0838	0.0735699	100.4328999
rs17772814	8	128711742	A	G	0.0857	-0.0746	0.0125	2.133E-09	0.0703011	-0.0286979	0.0571	0.614601	35.617024
rs180863361	10	114596377	T	C	0.9875	0.2313	0.0351	4.27E-11	0.991715	-0.130496	0.1374	0.3425	43.42472058
rs189966089	10	114886341	T	C	0.029	-0.1284	0.0221	6.173E-09	0.0195	-0.0170037	0.0978	0.8619	33.75557421
rs190054319	11	32595598	T	C	0.0554	-0.0815	0.0147	2.698E-08	0.0546025	-0.0966981	0.0522	0.0639102	30.73834976
rs2005705	17	36096300	A	G	0.4416	0.0532	0.0066	7.312E-16	0.455996	0.0304031	0.0264	0.2501	64.97337006
rs2010825	7	44188220	T	C	0.5097	-0.0382	0.0065	3.991E-09	0.511437	-0.0659048	0.0232	0.00447703	34.53822485

rs2201103	3	63781484	T	C	0.1848	-0.0492	0.0084	4.259E-09	0.183715	-0.00470103	0.0303	0.8769	34.30612245
rs223481	4	103688017	T	C	0.5186	0.035	0.0064	4.253E-08	0.512141	0.0341985	0.0229	0.1345	29.90722656
rs2248082	10	71457566	C	G	0.401	-0.0401	0.0066	1.208E-09	0.390718	-0.0292949	0.0244	0.2307	36.91483012
rs2272662	8	145639726	T	C	0.4123	-0.0402	0.0072	2.63E-08	0.438	-0.0138961	0.0295	0.6383	31.17361111
rs2456530	15	53091553	T	C	0.126	0.054	0.0096	2.071E-08	0.131	-0.0248974	0.0342	0.4668	31.640625
rs2464592	8	118183551	A	G	0.7122	-0.0496	0.0071	3.249E-12	0.698574	-0.00999983	0.0246	0.6849	48.80301527
rs2506125	9	84293453	A	G	0.4134	-0.0609	0.0066	2.643E-20	0.404856	0.0311013	0.0247	0.2076	85.14256198
rs2747567	20	32381337	A	G	0.4263	-0.0375	0.0067	2.191E-08	0.425	0.0288012	0.0246	0.2409	31.32657607
rs2783019	6	107422239	C	G	0.3403	-0.0383	0.0068	1.827E-08	0.309863	-0.0789025	0.0278	0.00447899	31.72339965
rs28663084	3	152382187	A	G	0.673	-0.0376	0.0068	3.298E-08	0.683144	-0.0176956	0.0245	0.4705	30.57439446
rs28681372	22	50351977	A	G	0.5908	-0.0396	0.0069	1.001E-08	0.603859	-0.0331019	0.0319	0.2994	32.93761815
rs28819812	4	157652753	A	C	0.3218	-0.0396	0.0072	4.22E-08	0.341711	-0.000300045	0.0239	0.9889	30.25
rs2898655	17	36053105	T	C	0.084	-0.0663	0.0119	2.783E-08	0.0881314	-0.0814053	0.0439	0.0638999	31.04081633

rs2925979	16	81534790	T	C	0.2987	0.0546	0.007	7.068E-15	0.28943	-0.00409839	0.0255	0.8718	60.84
rs297050	10	71306424	A	G	0.6441	0.0402	0.0067	1.984E-09	0.660141	0.0194987	0.0251	0.4377	36
rs3094515	17	36043653	T	C	0.3699	-0.0464	0.0072	1.339E-10	0.362718	-0.0492007	0.0255	0.0537205	41.5308642
rs316623	15	41801081	T	C	0.4463	0.0392	0.0065	1.554E-09	0.448289	-0.0168003	0.0239	0.4828	36.37017751
rs320369	1	118143517	A	G	0.3232	0.0372	0.0068	4.602E-08	0.302574	0.00410158	0.0248	0.8701	29.92733564
rs325	8	19819328	T	C	0.9011	0.0677	0.0108	3.438E-10	0.894141	0.0481995	0.0377	0.2004	39.29432442
rs34238147	13	26776255	A	G	0.2287	-0.0419	0.0076	4.207E-08	0.225004	-0.0249999	0.0276	0.3645	30.39490997
rs348330	1	229672955	A	G	0.6399	-0.0492	0.0067	2.1E-13	0.629856	0.0320993	0.0243	0.1868	53.92381377
rs35339173	10	80904919	T	C	0.2421	0.045	0.0077	6.324E-09	0.259	-0.0138049	0.0269	0.6084	34.15415753
rs3808636	8	41386465	T	G	0.0921	-0.0699	0.0115	1.286E-09	0.0949701	0.0344014	0.0441	0.4353	36.9452552
rs3809547	15	75628841	C	G	0.2409	-0.0417	0.0076	4.876E-08	0.274711	0.0114047	0.0261	0.662599	30.10543629
rs3816046	19	46118127	T	C	0.3238	0.0384	0.0069	2.746E-08	0.325282	0.0366983	0.0262	0.1612	30.97164461
rs3820981	2	165566877	A	G	0.5632	0.0402	0.0065	5.915E-10	0.570289	-0.0256974	0.0233	0.2696	38.24946746

rs3850071	13	80603592	A	C	0.6186	0.0457	0.0067	9.109E-12	0.623715	-0.039604	0.0265	0.1354	46.52461573
rs3851525	6	7014062	T	G	0.4094	0.0356	0.0065	4.157E-08	0.412144	-0.0328952	0.0234	0.1584	29.99668639
rs3862948	1	205099959	A	C	0.235	0.0414	0.0075	3.98E-08	0.233285	-0.0112025	0.0267	0.673501	30.4704
rs40270	5	55804552	A	C	0.2366	-0.0683	0.0075	1.304E-19	0.226856	0.0126003	0.0275	0.6468	82.93137778
rs4238013	12	4376089	T	C	0.7896	-0.0566	0.008	1.136E-12	0.80957	0.0247999	0.0326	0.4463	50.055625
rs4457053	5	76424949	A	G	0.6983	-0.0591	0.007	3.616E-17	0.685859	-0.0127003	0.0262	0.6296	71.28183673
rs4463188	5	44642670	T	C	0.6023	-0.0376	0.0065	6.949E-09	0.587574	-0.0313978	0.0241	0.1933	33.46177515
rs4466139	5	78430058	T	C	0.636	0.04	0.0066	1.327E-09	0.637285	0.000099995	0.0238	0.9957	36.73094582
rs4659326	1	117525810	T	C	0.2366	-0.045	0.0075	2.383E-09	0.223574	-0.0130042	0.0278	0.640301	36
rs4679370	3	124919777	T	C	0.4605	-0.0365	0.0064	1.098E-08	0.47	-0.0183982	0.0237	0.4373	32.52563477
rs4689381	4	6257188	T	C	0.4437	0.038	0.0066	8.369E-09	0.457715	-0.00569619	0.0228	0.8041	33.1496786
rs4813428	20	21451848	T	C	0.096	0.0599	0.0109	3.778E-08	0.0913465	-0.0855034	0.0406	0.0352403	30.19956233
rs4964654	12	108594043	A	T	0.3079	-0.0396	0.0069	1.001E-08	0.298722	-0.0730009	0.0263	0.00558805	32.93761815

rs520711	10	94220645	A	T	0.1909	0.0525	0.0082	1.289E-10	0.190718	-0.0286979	0.0295	0.3306	40.99122546
rs55752756	4	185672745	A	G	0.1699	-0.0485	0.0086	1.614E-08	0.175	0.00320486	0.0311	0.9178	31.8043537
rs55883527	15	77854352	T	G	0.3209	0.0439	0.0068	1.115E-10	0.313426	0.0199007	0.0249	0.4237	41.67841696
rs55938323	10	114773887	T	C	0.9891	-0.2268	0.0374	1.298E-09	0.988715	0.0723021	0.1193	0.5443	36.77417141
rs56218834	2	25520857	A	G	0.4251	-0.0354	0.0065	4.946E-08	0.448567	0.0235017	0.0234	0.3151	29.66059172
rs5758223	22	41489920	A	G	0.7134	0.0401	0.0071	1.779E-08	0.724711	0.0239997	0.0255	0.3468	31.89863122
rs6015379	20	57386639	A	G	0.4528	-0.0411	0.0064	1.234E-10	0.476715	-0.0159973	0.0229	0.4849	41.24047852
rs6020373	20	48837791	T	C	0.6137	-0.036	0.0066	4.825E-08	0.623578	-0.0593997	0.0248	0.0167699	29.75206612
rs60980157	9	139235415	T	C	0.2459	-0.0727	0.0082	5.489E-19	0.244996	0.0802981	0.0332	0.01557	78.60336109
rs61875108	10	114725079	A	G	0.0488	-0.093	0.0155	1.954E-09	0.0490989	0.0768036	0.0619	0.2153	36
rs62048489	16	53454855	T	G	0.0893	-0.0711	0.0124	8.674E-09	0.0834004	-0.0325025	0.0492	0.5085	32.87727627
rs62066054	17	9782028	A	G	0.3221	0.0379	0.0068	2.564E-08	0.298718	-0.0162005	0.0251	0.5177	31.0642301
rs62290256	3	185256242	A	G	0.9364	-0.0768	0.0134	1.001E-08	0.947859	-0.1268	0.0567	0.0253	32.84829583

rs62294592	3	186635584	T	C	0.1236	-0.054	0.0098	4.096E-08	0.10843	-0.00359646	0.0374	0.924	30.36234902
rs62451124	7	27968345	A	G	0.0573	-0.0801	0.0141	1.454E-08	0.0482873	-0.0299954	0.0548	0.584201	32.27206881
rs6459730	7	156916130	A	T	0.133	-0.0595	0.0097	9.931E-10	0.130141	0.0320993	0.0383	0.4024	37.62620895
rs6483215	11	92792115	A	G	0.233	0.0412	0.0074	2.987E-08	0.226141	0.0330962	0.0273	0.2248	30.99780862
rs6540807	1	214097482	T	C	0.5704	0.0349	0.0064	4.646E-08	0.552718	-0.0149005	0.023	0.5181	29.73657227
rs66930764	6	164103243	A	G	0.1288	-0.0532	0.0096	3.334E-08	0.132	0.0145043	0.0342	0.672699	30.71006944
rs672271	9	3273781	T	C	0.9062	-0.06	0.011	4.825E-08	0.903285	0.0406044	0.0398	0.3071	29.75206612
rs67721004	5	53290571	T	C	0.741	-0.0484	0.0072	2.092E-11	0.742574	-0.0369026	0.0262	0.1598	45.1882716
rs6829631	4	1781686	T	C	0.491	-0.0525	0.0066	1.733E-15	0.490996	0.0509022	0.0287	0.0755092	63.27479339
rs687621	9	136137065	A	G	0.6533	-0.0442	0.0071	5.364E-10	0.652437	-0.0473956	0.024	0.0478498	38.75500893
rs6918311	6	137287702	A	G	0.532	0.0445	0.0065	7.128E-12	0.512433	-0.0326988	0.0233	0.1598	46.86982249
rs7110293	11	34778351	A	C	0.1353	0.0509	0.0093	4.702E-08	0.133859	-0.0509986	0.0336	0.1293	29.9550237
rs7119618	11	128226431	C	G	0.7173	-0.0386	0.007	3.746E-08	0.723426	0.0301994	0.0254	0.2349	30.40734694

rs7146002	14	91871672	T	G	0.5729	-0.0358	0.0065	3.491E-08	0.58557	0.00039992	0.0233	0.9866	30.33467456
rs7234998	18	7068724	T	C	0.3736	0.0364	0.0066	3.423E-08	0.363289	-0.021898	0.0243	0.3677	30.41689624
rs7250869	19	33887405	T	C	0.3129	0.0379	0.0069	4.146E-08	0.319004	-0.0383043	0.0248	0.1222	30.17034237
rs72655474	9	22139684	C	G	0.0217	-0.1908	0.025	2.446E-14	0.021157	-0.00879859	0.1013	0.9311	58.247424
rs72802340	16	75232528	T	G	0.0418	-0.1307	0.017	1.303E-14	0.0333574	0.0625991	0.0817	0.4439	59.10896194
rs72940580	18	52848102	A	G	0.9529	-0.0842	0.0154	4.484E-08	0.964285	0.0247024	0.0636	0.6979	29.89391128
rs72999033	19	19366632	T	C	0.0659	0.0837	0.013	1.142E-10	0.0613011	0.00249688	0.0482	0.9584	41.45378698
rs7313918	12	118394008	T	C	0.8635	-0.0559	0.0094	2.99E-09	0.867144	-0.0213973	0.0353	0.543501	35.36453146
rs73390986	22	30135928	T	C	0.9088	0.0764	0.0113	1.415E-11	0.917715	-0.0398017	0.0415	0.3382	45.71195865
rs7433808	3	64727086	A	T	0.7617	0.0505	0.0075	2.1E-11	0.73	0.0384031	0.0263	0.1435	45.33777778
rs7568063	2	226840831	T	C	0.8392	0.064	0.009	1.181E-12	0.84257	-0.00749804	0.0361	0.8363	50.56790123
rs76074250	11	2073182	A	G	0.7971	-0.0463	0.0081	9.305E-09	0.794	0.0188021	0.0293	0.5203	32.67322055
rs76079449	12	121425452	T	C	0.0226	0.1194	0.0218	4.192E-08	0.023344	-0.0785996	0.0826	0.3414	29.99823247

rs7615486	3	185308495	A	C	0.9655	-0.0989	0.0177	2.281E-08	0.923856	0.0349037	0.053	0.5098	31.22094545
rs7615580	3	12027240	T	C	0.9344	0.1052	0.0131	9.13E-16	0.937856	0.0146029	0.0475	0.758501	64.48948196
rs76367336	2	227228251	T	C	0.8863	0.0584	0.0103	1.259E-08	0.871574	-0.0231049	0.0351	0.5105	32.14779904
rs7646490	3	122936084	A	G	0.2113	-0.054	0.008	1.152E-11	0.206718	-0.0340016	0.0289	0.2392	45.5625
rs77460585	5	101123995	A	G	0.0486	0.0878	0.0152	7.328E-09	0.0418563	0.101301	0.0586	0.0839595	33.3658241
rs77655131	7	102086552	T	C	0.1301	0.0554	0.0097	1.274E-08	0.107004	-0.0850025	0.0408	0.0370502	32.61940695
rs7775748	6	126623947	T	C	0.4487	0.0399	0.0065	7.923E-10	0.45343	-0.010697	0.0235	0.6495	37.68071006
rs78011501	12	121097000	A	G	0.0449	0.0913	0.0155	3.819E-09	0.0428993	0.00239712	0.058	0.9672	34.6959001
rs78683917	2	65562302	T	C	0.9729	0.1115	0.0203	4.035E-08	0.96957	0.0627964	0.0674	0.3512	30.16877381
rs7897943	10	12239456	A	G	0.7531	-0.0478	0.0074	1.276E-10	0.75943	-0.0138049	0.027	0.6077	41.72461651
rs8030349	15	90362275	A	G	0.3523	0.0412	0.0069	2.491E-09	0.344148	-0.040697	0.0271	0.133	35.65301407
rs9309324	2	60546132	T	G	0.4857	0.0414	0.0064	9.047E-11	0.497285	-0.00670241	0.0231	0.7728	41.84472656
rs9316500	13	51094114	T	G	0.7121	0.0387	0.007	3.454E-08	0.690433	-0.0416036	0.0245	0.08916	30.56510204

rs9379084	6	7231843	A	G	0.1121	-0.0994	0.0106	5.478E-21	0.140715	-0.0144032	0.0368	0.696299	87.93485226
rs9520799	13	108781439	A	T	0.859	-0.0509	0.0092	3.308E-08	0.849437	-0.0805053	0.0318	0.0112899	30.60975898

Abbreviation: T2DM= type 2 diabetes mellitus, CD= Crohn's disease, SNPs= single-nucleotide polymorphisms, Chr= Chromosome, EA= Effect Allele, OA= Other Allele, EAF= effect allele frequency, SE= standard error.

Table S16. Characteristics of 44 AMPK variants

SNP	EA	OA	EAF	β	SE	*P*-value
rs11239944	A	G	0.825	-0.018	0.0061	1.88E-04
rs2059409	T	C	0.045	-0.011	0.0047	1.16E-02
rs1365964	G	A	0.801	-0.0057	0.0029	6.91E-03
rs11884246	A	C	0.522	-0.0033	0.0017	2.61E-02
rs6726126	A	G	0.518	-0.0038	0.0018	3.38E-07
rs16858808	A	G	0.04	-0.025	0.012	2.36E-03
rs17572109	A	G	0.208	-0.0055	0.002	1.54E-05
rs7596500	G	T	0.982	-0.016	0.0074	8.87E-03
rs3816560	C	T	0.221	-0.004	0.0019	2.07E-05
rs10230736	G	A	0.862	-0.011	0.0038	4.13E-04
rs1808593	T	G	0.827	-0.0049	0.0024	3.83E-03

rs1563636	C	T	0.196	-0.0057	0.0025	1.94E-03
rs7806203	C	T	0.345	-0.0039	0.002	6.75E-03
rs7780461	T	C	0.118	-0.0063	0.0032	1.16E-02
rs10259821	A	G	0.31	-0.0037	0.0019	1.20E-03
rs1635527	C	G	0.525	-0.0038	0.0019	4.35E-36
rs1859444	C	T	0.808	-0.0048	0.0024	1.42E-22
rs1859443	G	A	0.856	-0.0058	0.0025	6.27E-13
rs11168355	G	A	0.792	-0.0055	0.0022	2.63E-43
rs11168359	A	G	0.119	-0.015	0.0032	6.94E-91
rs12297820	A	G	0.119	-0.017	0.0029	1.09E-43
rs10492081	A	G	0.81	-0.0071	0.0024	9.20E-21
rs17614932	A	C	0.975	-0.012	0.0051	2.70E-11
rs10875764	C	T	0.584	-0.0077	0.0038	1.32E-13

rs7134565	C	T	0.469	-0.0035	0.0018	3.79E-37
rs2732480	A	C	0.425	-0.012	0.002	1.07E-142
rs1489107	A	G	0.031	-0.014	0.0058	2.16E-22
rs2932091	A	C	0.325	-0.0043	0.0021	1.25E-03
rs10875801	C	T	0.825	-0.0089	0.0023	5.95E-30
rs4760702	A	T	0.967	-0.011	0.0048	1.16E-09
rs7959684	A	G	0.226	-0.005	0.0021	1.30E-50
rs11168547	T	C	0.943	-0.0079	0.0026	1.06E-26
rs12582586	T	C	0.212	-0.005	0.0024	1.92E-04
rs10875814	G	A	0.733	-0.0051	0.0024	1.34E-09
rs12582811	G	T	0.128	-0.0089	0.0033	1.96E-06
rs11168643	A	G	0.034	-0.018	0.0061	3.84E-08
rs10875843	A	G	0.033	-0.0093	0.0045	4.79E-08

rs7975821	A	G	0.724	-0.0041	0.0021	2.81E-07
rs17834622	A	G	0.346	-0.0064	0.0019	1.10E-17
rs12830014	G	A	0.914	-0.0074	0.0037	8.62E-04
rs10783277	C	T	0.674	-0.0045	0.0019	4.35E-05
rs12322783	A	G	0.189	-0.0045	0.0023	1.80E-08
rs17197593	T	C	0.053	-0.014	0.0062	5.50E-05
rs1050187	C	T	0.704	-0.0042	0.0019	1.71E-03

Abbreviation: AMPK= AMP-activated protein kinase, SNPs= single-nucleotide polymorphisms, EA= Effect Allele, OA= Other Allele, EAF= effect allele frequency, SE= standard error.
